# Tall adhesions overcome cell surface steric interference and rescue antibody-dependent phagocytosis in macrophages

**DOI:** 10.1038/s41467-026-78095-7

**Published:** 2026-09-28

**Authors:** Aymeric Chorlay, Aaron M. Joffe, Jared Huzar, Jaffar Hasnain, Caitlin E. Cornell, Phillip L. Geissler, Daniel A. Fletcher

**Affiliations:** 1 Department of Bioengineering, University of California Berkeley, Berkeley, CA USA; 2 UC Berkeley/UC San Francisco Graduate Group in Bioengineering, Berkeley, CA USA; 3 Biophysics Graduate Group, University of California, Berkeley, Berkeley, CA USA; 4 Department of Chemistry, University of California, Berkeley, Berkeley, CA USA; 5 Chemical Sciences Division, Lawrence Berkeley National Laboratory, Berkeley, CA USA; 6 Biological Systems and Engineering Division, Lawrence Berkeley National Laboratory, Berkeley, CA USA; 7 Chan Zuckerberg Biohub, San Francisco, CA USA

**Keywords:** Thermodynamics, Immune evasion, Biological physics, Phagocytes

## Abstract

Macrophages phagocytose IgG-opsonized target cells by forming phagocytic synapses upon binding of IgG to Fcγ receptors (FcγR) expressed on the macrophage surface. However, binding requires accessibility of both IgG and FcγR within densely packed glycocalyces that decorate the macrophage and target cell surfaces. Here, we use cell-like target particles to show that non-binding ‘bystander’ proteins not directly involved in phagocytic signaling can sterically interfere with FcγR engagement, ultimately inhibiting synapse formation and phagocytosis. Furthermore, we demonstrate that adding a tall binding protein to the target particle overcomes bystander-mediated inhibition and substantially recovers phagocytosis in a process we call kinetic enhancement. We confirm that tall binders are sufficient to drive short binder engagement in vitro and introduce theory and simulations to explain the nonmonotonic dependence of phagocytosis on tall binder surface density. These findings show that kinetic enhancement is a general and tunable feature of interface formation, providing a strategy for re-engineering transition states to promote short receptor engagement at cell-cell contacts.

## Abstract (web summary)

Antibody-dependent cellular phagocytosis relies on binding the Fc region of IgG antibodies to Fcγ receptors (FcγR) expressed by macrophages. However, tall neighboring bystander proteins can sterically hinder FcγR engagement. Here, the authors combine model cell-based experiments, cell-free systems, and in silico modeling to show that adding tall binding proteins promotes close contacts, supports the formation of a stable phagocytosis synapse, and restores phagocytosis.

## Introduction

Cell surfaces are crowded environments consisting of thousands of membrane-bound molecules per square micron that extend beyond the plasma membrane by tens to hundreds of nanometers, or more^1^. At sufficient densities and glycosylation levels, these molecules create a steric barrier that can inhibit close contact and subsequent receptor binding between cells, especially for short binders. Many cells, such as macrophages, rely on close contact to carry out their functions. During an immune response, macrophages are critical actors that recognize and clear pathogens and foreign objects by phagocytosis, while also initiating broad-scale immune responses by secreting inflammatory cytokines and presenting antigens to adaptive immune cells^2–4^. Although macrophages recognize many classical pathogens using a diverse set of pattern recognition receptors on their surface, detection of novel targets, including highly mutagenic pathogens and host-derived cancer cells, often relies on antibody recognition via Fc receptors. In one version of this detection strategy, IgG antibodies serve as adaptor molecules that bind to unique antigen epitopes on the pathogen surface via their Fab regions and make the target susceptible to recognition by Fcγ receptors (FcγR) through binding to the Fc region of IgG. This mechanism of phagocytosis, known as antibody-dependent cellular phagocytosis (ADCP), has been harnessed to target and clear cancer cells during treatment with antibody-based immunotherapies^5,6^.

Therapeutic strategies that maximize activation and minimize inhibition of macrophages are being pursued through multiple avenues. The most direct routes are developing specific targeting antibodies that enhance activation signals or high-affinity blocking antibodies that disrupt inhibitory checkpoints^7^. In previous work, we studied the effect of antigen height on ADCP and found that antibodies targeting antigens with short extracellular domains (<10 nm) were more efficient at triggering phagocytosis due to the requirement for size-dependent segregation of CD45 phosphatase molecules from the phagocytic interface^8^. We also showed that adding CD47 to the IgG-coated surface inhibited phagocytosis in a ratiometric way^9^. In these studies, we used reconstituted cell-like target particles with only the proteins of interest—activating IgG-bound antigens and/or inhibitory CD47—that allowed us to isolate the role of specific molecular players in phagocytosis. However, it remains unclear how the complex surface of a real target cell, which contains many molecules not directly involved in phagocytic signaling, might affect synapse formation and FcγR engagement.

Short antigens typically targeted by therapeutic antibodies on tumor cells constitute only a small subset of all surface proteins and are surrounded by a dense array of tall non-antigenic proteins^10,11^, including tall, heavily glycosylated proteins such as MUC1, P-selectin, and CEACAM5. One might expect that the presence of these non-antigenic proteins, which we refer to as ‘bystander’ proteins, could act as a steric barrier to IgG-FcγR binding^12,13^, as has been suggested for T-cell activation^14^. This idea is consistent with previous simulations^15^ and the use of poly(ethyleneglycol) (PEG) polymer brushes to block molecular adsorption to surfaces^16,17^. Steric barriers to phagocytosis would be most extreme in the case of IgG bound to short antigens on the surface of cancer cells, especially given that cancer cells have been shown to upregulate the expression of tall glycoproteins^18,19^. Indeed, cell surface crowding has been demonstrated to impose an energy penalty on the scale of 1-3k_B_T for an IgG in solution binding to buried receptors^12^. In contrast to better studied inhibitory checkpoints such as CD47-SIRPα^9^, steric barriers created by bystander proteins represent a potentially potent and ubiquitous inhibitory mechanism that is poorly understood and for which therapeutic strategies to overcome it are lacking.

In this work, we use cell-like reconstituted target particles to explore the effect of bystander protein height and surface density on ADCP. Using live cell imaging, we quantify bystander-mediated inhibition of phagocytosis as a function of bystander protein height and abundance. Counterintuitively, we find that adding a long macrophage-binding protein (P-selectin) to target surfaces restores efficient phagocytosis. We call this effect ‘kinetic enhancement’ to highlight the increased rate of close-contact formation (FcγR–IgG binding) in the presence of long binding proteins compared to their absence. To test whether kinetic enhancement requires active cellular processes, we reconstitute membrane interface formation using giant unilamellar vesicles, DNA binders, and purified bystander proteins. We observe results similar to those obtained with macrophages, suggesting that steric inhibition of interface formation by bystander proteins and subsequent recovery with long binders is a general phenomenon that does not require active cellular processes. To clarify the microscopic mechanisms underlying kinetic enhancement, we use theory and computer simulations to show that bystander-mediated energetic barriers to phagocytosis can be crossed in a reasonable time only if the corresponding transition state involves strong local deformation in membrane shape in the form of a “dimple” of IgG–FcγR binding that progressively offsets the entropic cost of excluding bystanders from the nascent synapse. Tall adhesion proteins stabilize a distinct but similarly dimpled state, with the potential to greatly increase the rate of close-contact formation. Their binding, however, also establishes an undimpled intermediate state that becomes deeply metastable at high density (or strong binding) of the tall adhesion proteins, with the potential to greatly impede close contact.

These findings add to our understanding of phagocytic synapse formation and FcγR engagement, showing that while targeting IgG antibodies to short antigens most efficiently drives phagocytic synapse formation and subsequent phagocytosis, tall, non-binding proteins around those short antigens sterically impede phagocytic synapse formation in the first place. We demonstrate that the energetic barriers introduced by bystander proteins can be overcome with additional tall binders to facilitate barrier crossing, an approach that cells may already use to promote short ligand–receptor pair engagement in physiological contexts and one that can potentially be co-opted for therapeutic purposes.

## Results

### Non-binding bystander proteins can inhibit antibody-dependent phagocytosis

**Fig. 1:**
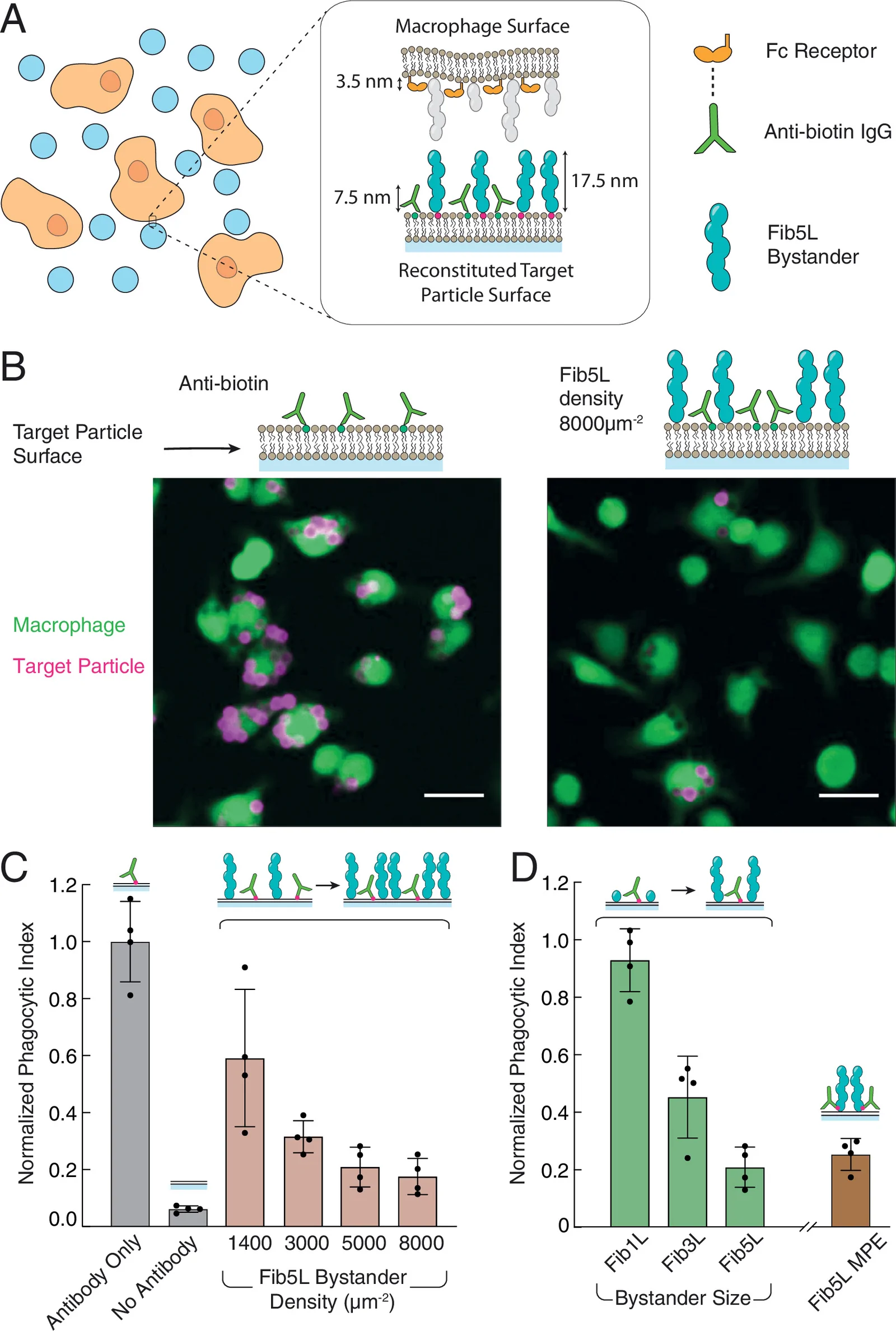
Bystander proteins on target membranes can inhibit antibody-mediated cellular phagocytosis. **A** Macrophages are incubated with reconstituted phagocytic targets to assay phagocytosis. Important proteins involved in phagocytic interface formation are illustrated along with their respective sizes. **B** Fluorescence images showing macrophages incubated with reconstituted targets containing anti-biotin IgG, with or without the addition of the Fib5L bystander protein. Excess beads were washed out before imaging. Similar results were obtained with *N* = 4 independent biological replicates. Scale bars: 20 µm. **C** Phagocytic index of reconstituted targets displaying both anti-biotin IgG targeting biotin lipids and Fib5L bystander protein. The first two bars represent a positive control (targets displaying only anti-biotin IgG) and a negative control (targets displaying no anti-biotin IgG), respectively. Phagocytic index decrease over Fib5L density *p* < 0.0001 by two-sided t- test on the slope for a simple linear model against a constant model. Welch’s two-sided t-test comparing the antibody-only condition to different Fib5L densities showed significant differences at 1400, 3000, 5000, and 8000 µm⁻² (*p* = 0.034, 0.0009, 0.0003, and 0.0004, respectively). **D** Left: Phagocytic index of reconstituted targets opsonized with anti-biotin IgG bound to biotinylated lipids with Fib5L, Fib3L, or Fib1L bystander proteins (8000 µm⁻²). Right: Phagocytic index of reconstituted targets bearing Fib5L converted into a membrane-proximal epitope (MPE) by C-terminal biotinylation and opsonized with anti-biotin IgG (~8000 molecules µm⁻²). Welch’s two-sided t-test yielded *p*-values of 0.0022 (Fib1L vs. Fib3L), 0.033 (Fib3L vs. Fib5L), and 0.36 (Fib5L vs. Fib5L MPE). For all graphs, bars represent the mean value; error bars represent the standard deviation for *N* = 4 independent biological replicates.

To determine whether bystander proteins can inhibit macrophage antibody-dependent phagocytosis, we created reconstituted target particles to model antibody-opsonized cells and added bystander proteins with controlled densities and heights using a previously described method^20^. Briefly, each particle consists of a glass bead coated with a fluid lipid bilayer to model the fluid plasma membrane of a target cell, with His-tagged proteins of interest directly coupled to Ni-NTA lipids in the bilayer and monoclonal IgG antibodies bound to specific antigens (Fig. 1A). In our experiments, biotinylated lipids were incorporated into the lipid bilayer so that anti-biotin IgG could be bound directly to the particle surface. This configuration models an antibody bound to a short antigen, which we previously found to efficiently trigger phagocytic signaling due to size-based segregation of the CD45 phosphatase from the phagocytic interface^8^. We used mouse IgG1 that engages FcγRIII and FcγRIIB in RAW 264.7 cells to drive uptake of opsonized targets^21^. All experimental conditions used a fixed surface density of anti-biotin IgG1 of 200 µm^−2^ as described in ref. ^8^.

To test how bystander protein size and surface density affect ADCP, we used synthetic proteins made of repeated stable consensus FN-III domains (Fibcon)^22^ that lack the RGD integrin-binding motif. We conjugated these proteins to Ni-NTA lipids in the supported bilayer via C terminal histidine tags. We chose Fibcons as bystanders based on prior work showing that they do not induce phagocytosis alone^8^. Consistent with this, incubating soluble Fibcons with our model cells (RAW 264.7) produced no detectable binding at the cell surface (Supplementary Fig. 2), suggesting no interaction with macrophage integrins.

The Fibcon proteins used in our experiments contained 1, 3, or 5 domain repeats (Fib1L, Fib3L, and Fib5L, respectively), corresponding to fully extended protein heights of 3.5 nm, 10.5 nm, and 17.5 nm. Their actual heights on membranes are influenced by their flexibility, which we have previously measured^23^, and their density on the surface. The density of bystander proteins displayed on the surface of target particles was controlled by changing the amount of Ni-NTA lipids in the bilayer. Absolute densities of Fibcon proteins were measured with flow cytometry using fluorescently-labeled versions of the proteins calibrated to beads with known fluorophore numbers (Supplementary Fig. 1).

As a first test of bystander-mediated inhibition, we compared phagocytosis of target particles displaying only anti-biotin antibodies with target particles displaying both anti-biotin antibodies and bystander proteins. We began with Fib5L as the bystander because its fully-extended height of 17.5 nm is closest to the average cell surface protein height of 15 nm^23^. The Fib5L bystander proteins were displayed at a density of 8000  µm^−2^, which is below reported bulk cellular surface densities of approximately 20,000 µm^−2^^24^. The target particles were incubated in a 96-well plate with RAW 264.7 macrophage-like cells for 30 min at 37 °C, followed by washing of excess particles from the well. Fluorescent imaging of the macrophages after incubation revealed dramatically less phagocytosis for conditions in which bystander proteins were present compared to those without bystander proteins (Fig. 1B).

We next quantified phagocytosis across a range of bystander densities and heights, alongside a negative control lacking anti-biotin IgG and a positive control containing only anti-biotin IgG with no bystander proteins. Defining the average value of internalized target fluorescence per macrophage as the ‘phagocytic index’, we observed a steady decrease in phagocytosis with increasing density of Fib5L bystanders (Fig. 1C). To determine the effect of bystander protein height on phagocytosis, we then fixed the density of bystander proteins to be 5000 µm^−2^ but varied the height by using Fib1L, Fib3L, or Fib5L. Quantification of phagocytic indices for these conditions showed that phagocytosis decreased with increasing bystander height (Fig. 1D).

Because short antigens more efficiently induce phagocytosis, we asked whether tall antigens—which typically elicit poor phagocytosis—could be rendered equally effective by directing antibodies to membrane-proximal epitopes (MPE). To test this, we converted Fib5L into an antigen by biotinylating the membrane-proximal C-terminus of Fib5L, added anti-biotin IgG to Fib5L at a surface density of 8000  µm^−2^, and quantified phagocytosis. Even though antibody density is significantly higher than for the case of biotinylated lipids, we found that phagocytosis was still blocked (Fig. 1D). This data suggests that targeting the membrane-proximal epitope of a tall antigen will not enhance phagocytosis since the tall antigen effectively blocks FcγR engagement, similar to a bystander protein at high surface densities.

When antibodies are attached to a membrane-distal epitope of antigens with increasing height (biotinylated Fib1L, Fib3L, and Fib5L) and also surrounded by tall bystander proteins (Fib5L), we found that phagocytosis modestly recovers only for biotinylated Fib3L, the tallest antigen that is still capable of creating a membrane gap, when bound to FcγR, that excludes CD45 (Supplementary Fig. 3). Taken together, these results indicate that bystander proteins can sterically inhibit phagocytosis of antibody-opsonized particles.

### Bystander proteins impede FcγR engagement and dynamics at phagocytic interfaces

**Fig. 2:**
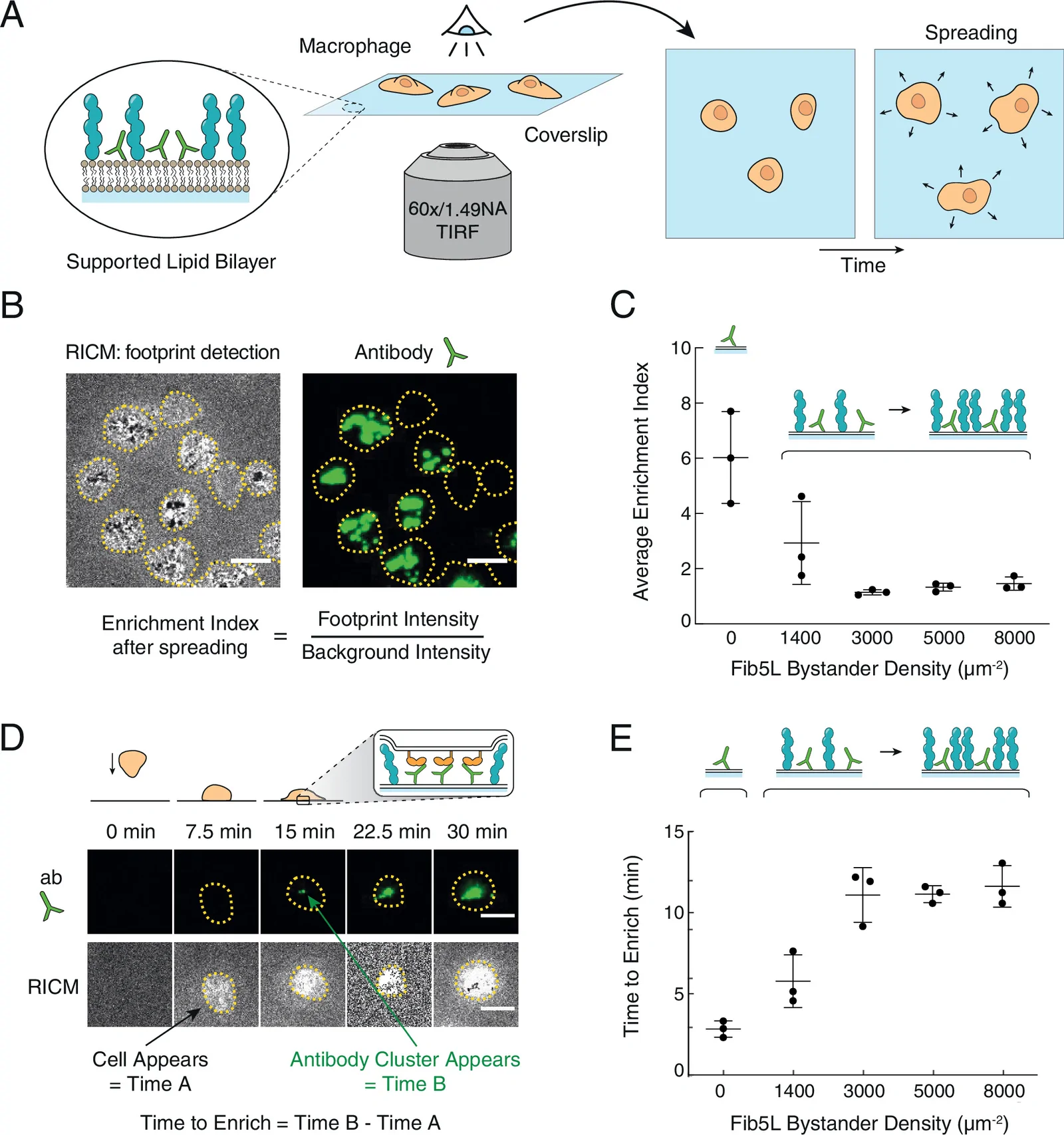
Bystander proteins sterically block antibody engagement with macrophage Fcγ receptors. **A** Schematic view of TIRF live-cell imaging experiments to visualize the phagocytic interface. **B** TIRF images of fluorescently labeled anti-biotin IgG were captured alongside RICM images of macrophages. The enrichment index was quantified by measuring the fluorescence intensity within each macrophage interface (masked in the RICM channel) and dividing by the background fluorescence intensity. Images were captured after a 30-minute incubation to represent the equilibrium state. Similar results were obtained with *N* = 3 independent biological replicates. Scale bars: 10 µm. **C** Enrichment indices of IgG on SLBs by macrophages as a function of Fib5L bystander protein density. Enrichment significantly decreases with increasing Fib5L density (two-sided t- test on the slope, linear vs constant model; *p* = 0.0061). **D** TIRF images of fluorescently labeled anti-biotin IgG were captured alongside RICM images of macrophages every 30 seconds for a total of 30 min. The time to enrich was measured by subtracting the time at which a macrophage first appeared in the RICM channel from the time at which anti-biotin IgG clustering was first observed in the TIRF channel. Scale bars: 10 µm. **E** Time to IgG enrichment for macrophages on SLBs displaying different densities of the Fib5L bystander protein. Enrichment time increased significantly with Fib5L density (two-sided t- test on the slope comparing linear vs constant model, *p* = 0.0002). For all graphs, bars represent the mean value; error bars represent the standard deviation for *N* = 3 independent biological replicates with *n* > 20 cells.

To confirm that bystander proteins are inhibiting phagocytosis by interfering with phagocytic interface formation and IgG-FcγR binding, we directly imaged interface formation using total internal reflection fluorescence (TIRF) microscopy. To do this, we created supported lipid bilayers (SLBs) on glass coverslips using the same IgG and bystander protein compositions used to test phagocytosis with glass beads. We then dropped macrophages onto the SLBs coated with IgG and bystander proteins and tracked fluorescent anti-biotin IgG dynamics at the interface after engagement with macrophage FcγRs (Fig. 2A). We also captured images using Reflection Interference Contrast Microscopy (RICM) to quantify the contact area of each macrophage, regardless of its ability to bind and cluster antibody.

First, we characterized the amount of anti-biotin IgG bound to FcγR at the interface between each macrophage and the SLB after 30 min of incubation, which is the point at which interfaces reached a quasi-steady state. To do this, we used RICM to identify each macrophage in the field of view and created a mask around its area. Next, we quantified an ‘enrichment index’ (EI), which quantifies antibody enrichment at the phagocytic interface, by measuring the average IgG fluorescence intensity within each macrophage mask and dividing it by the average IgG intensity outside of the mask (Fig. 2B).

We observed a significant decrease in EI for cells as the density of bystander Fib5L was increased, dropping from an average value of 6 down to 1.1 in the presence of bystander proteins at a surface density of 3000 µm^−2^ ± 500 µm^−2^ (Fig. 2C). This observation is consistent with bystander proteins inhibiting phagocytosis by blocking phagocytic interface formation. Under high crowding bystander conditions (8000  µm^−2^), treating macrophages with latrunculin A (LatA) did not significantly alter antibody enrichment at the interface (Supplementary Fig. 4), indicating that blocking is independent of actin.

To further characterize the effect of bystander proteins on IgG-FcγR binding, we used time-lapse imaging to observe the dynamics of interface formation. Using the same conditions as in the enrichment index measurements, we captured images of macrophages interacting with the SLB every 30 sec for 30 min. For each macrophage that formed antibody clusters within that timeframe, we measured the time to enrich (*t*_*enrich*_) as the difference between the time at which the first IgG cluster appeared in TIRF and the time at which the macrophage first settled on the surface in RICM (Fig. 2D). The average value for *t*_*enrich*_ was 2.8 min for macrophages binding to SLBs displaying only IgG. However, when bystander Fib5L was added at a surface density of 1400 ± 200 µm^−2^, this value rose to 5.8 min. For bystander Fib5L surface densities of 3000 ± 500 µm^−2^ and above, *t*_*enrich*_ increased to 11 min (Fig. 2E).

This observation indicates that bystander proteins not only lessen the equilibrium number of IgG-FcγR complexes formed at the phagocytic interface at long times but also decrease the rate at which clusters form. Considering that average speeds of tissue-resident macrophages have been measured to be on the order of 1 µm/minute^25^, the average contact time between a macrophage and a potential target cell would last less than approximately 11 min (assuming a stationary target cell with a diameter of 10 µm). The measured shift in *t*_*enrich*_ from 2.8 min to 11 min due to the increased surface density of bystander proteins therefore has the potential to significantly alter the likelihood of phagocytic synapse formation and signaling over physiologically relevant timescales.

### P-selectin, a tall binding protein, can partially rescue phagocytosis by overcoming bystander-mediated inhibition

Our results indicate that the presence of bystander proteins can substantially inhibit the engagement of short binding partners at the macrophage-target interface. In addition to these non-adhesive bystander proteins, natural cell-cell interfaces are also populated by a variety of long adhesion proteins. Like bystanders, these species must be excluded from an interface to permit close contact and IgG-FcγR binding, carrying an even steeper thermodynamic cost due to the disruption of favorable contacts or the exclusion of bound pairs of binders. However, unlike bystanders, tall binders can stabilize the initial cell–cell contact. From this stabilized attachment, a phagocytic synapse could form with minimal reorganization—namely, membrane deformation that enables short-binder engagement and excludes both the tall binder and bystander proteins. We hypothesized that such tall binders could, under some circumstances, alleviate bystander-mediated inhibition of phagocytic synapse formation and restore phagocytosis.

We experimentally tested this idea by adding P-selectin to target particles, along with Fib5L bystander proteins and IgG bound to biotinylated lipids. P-selectin is typically expressed by activated platelets and endothelial cells and is known to bind to its ligand PSGL-1 on leukocytes and leukocyte-derived cells, including macrophages^26^. It is a relatively rigid protein with a measured height of 38 nm^27,28^ that binds to PSGL-1 at its distal end. We chose P-selectin because (i) it is longer than the Fibcon-based bystander proteins, enabling easy binding, (ii) it binds to the macrophage without directly activating or inhibiting phagocytic signaling, and (iii) its affinity for PSGL-1 is low^29^, so that binding of the FcγR to IgG^30^ is still the favored state at equilibrium. Interestingly, P-selectin is known to be critical for leukocyte transmigration, which involves leukocyte rolling along the endothelial lining, arrest, and ultimately the formation of stable integrin binding events that initiate transmigratory signaling^31,32^. We wondered if P-selectin present on the surface of a phagocytic target could—potentially mirroring its role in transmigration—yield kinetic enhancement of IgG-FcγR binding.

**Fig. 3:**
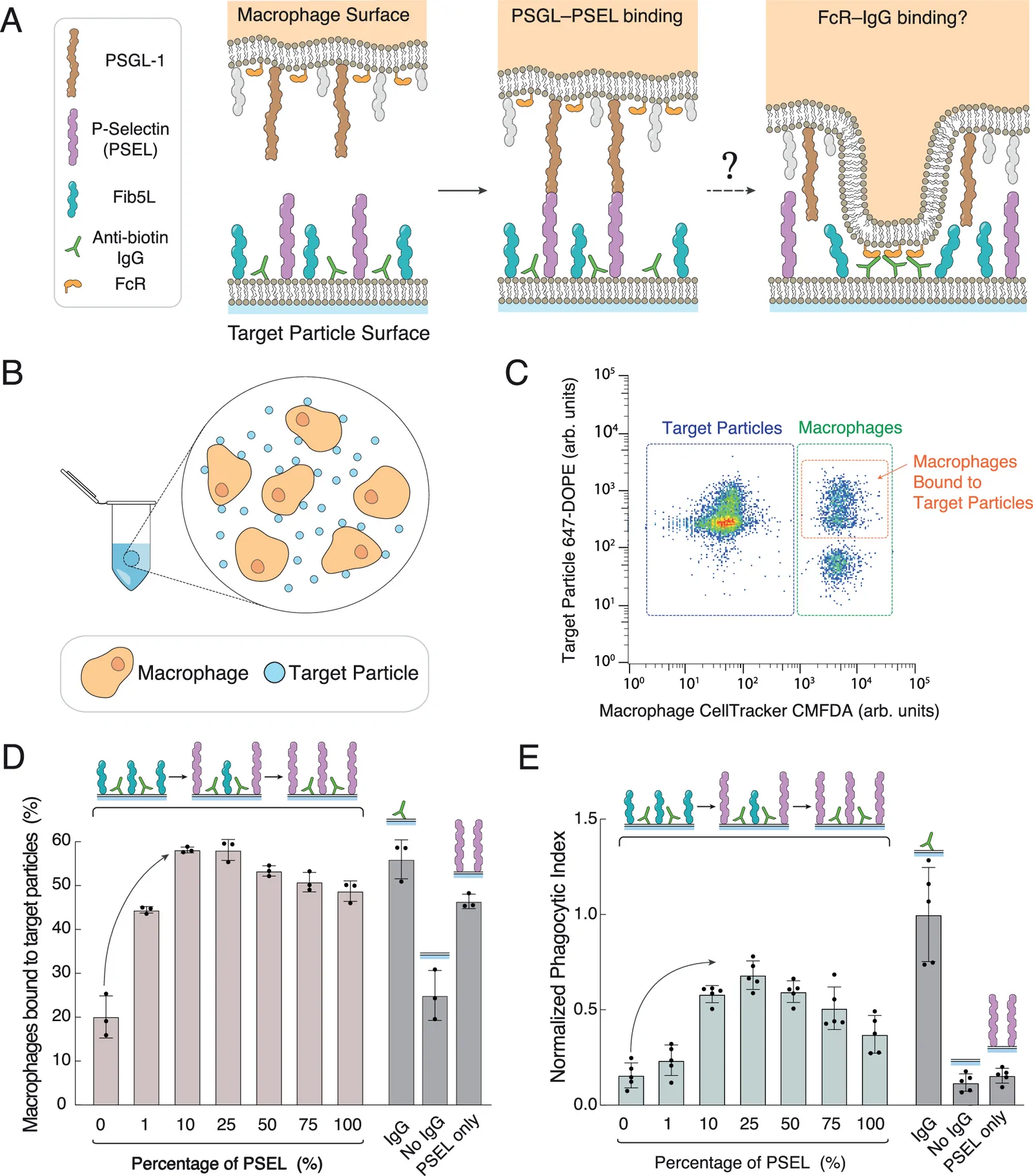
Addition of tall binding proteins to the target membrane can rescue phagocytosis of bystander-blocked particles. **A** Illustration of a reconstituted target particle surface containing anti-biotin IgG, Fib5L bystander proteins, and tall binding protein P-selectin (PSEL). Upon binding of P-selectin to its ligand PSGL-1 on the macrophage surface, the interface can remain in the metastable state, with PSEL bound to PSGL-1, or form a phagocytic interface through engagement of IgG with the macrophage FcγR, leading to phagocytosis. **B** To assay interface formation, macrophages are incubated with reconstituted target particles at 4 °C, which inhibits phagocytosis. **C** Flow cytometry shows distinct populations of target particles, macrophages, and macrophages bound to target particles. Macrophage binding to target particles is quantified by gating macrophages that display target particle fluorescence (647-DOPE signal; red dashed box) and normalizing to the total macrophage population (green dashed box; gated on CellTracker Green CMFDA signal). **D** Quantification of interface formation for target particles from flow cytometry data displaying varying amounts of anti-biotin IgG, Fib5L bystander protein, and PSEL. The first two bars represent positive and negative controls, respectively, and define the dynamic range of the measurement. The addition of PSEL on target particles leads to an increase in interface formation above levels containing only Fib5L bystander (indicated by arrow). A two-sided t-test yielded *p*-values of 0.0113 (0% PSEL vs. 1% PSEL), <0.0001 (1% PSEL vs. 10% PSEL), 0.998 (10% PSEL vs. 25% PSEL), 0.035 (25% PSEL vs. 50% PSEL), 0.152 (50% PSEL vs. 75% PSEL), 0.32 (75% PSEL vs. 100% PSEL), 0.324 (0% PSEL vs. No IgG), 0.0007 (0% PSEL vs. IgG only), and 0.0033 (PSEL only vs. No IgG). **E** Quantification of phagocytosis of target particles displaying the same surface molecules as in D. The addition of PSEL on the target surface leads to an increase in phagocytosis above levels containing only Fib5L bystander (indicated by arrow). A two-sided t-test yielded *p*-values of 0.13 (0% PSEL vs. 1% PSEL), <0.0001 (1% PSEL vs. 10% PSEL), 0.034 (10% PSEL vs. 25% PSEL), 0.073 (25% PSEL vs. 50% PSEL), 0.16 (50% PSEL vs. 75% PSEL), 0.32 (75% PSEL vs. 100% PSEL), 0.321 (0% PSEL vs. No IgG), 0.001 (0% PSEL vs. IgG only), and 0.22 (PSEL only vs. No IgG). For all graphs, bars represent the mean value; error bars represent the standard deviation for *N* = 3 and 5 independent biological replicates for D and E, respectively.

We first investigated whether P-selectin displayed on a target particle’s surface would increase macrophage-target binding. We created target particles with IgG antibody, Fib5L bystander protein, and purified P-selectin containing a C-terminal His-tag that was conjugated to Ni-NTA lipids in the target particle bilayer (Fig. 3A). As in all previous experiments, the anti-biotin IgG density was fixed at a surface density of 200 µm^-2^. The total combined surface density of Fib5L bystander plus P-selectin was set at 8000  µm^-2^ by fixing the total concentration of proteins added to the target particles, while the relative molar percent of P-selectin was varied from 0% to 100%.

To determine if interface formation occurs in the absence of any phagocytosis, we first incubated fluorescently labeled macrophages and targets in tubes under constant rotation at 4 °C, a temperature at which macrophages can bind phagocytic targets but are unable to phagocytose them (Fig. 3B). We ran the contents of the tube through a flow cytometer and plotted fluorescence intensities from the target particles and macrophages on orthogonal axes (Fig. 3C). Using this target binding assay, the macrophage population could be easily distinguished from the target particle population, and the macrophages showed two distinct sub-populations: those with target fluorescence and those with no target fluorescence. Because the targets could not be internalized by the macrophages, any macrophages showing target particle fluorescence must have had targets bound to their surface.

To compare target binding under different conditions, we calculated the percentage of macrophages that contained targets bound to their surface. We first measured binding of targets displaying only anti-biotin IgG on their surface and those without IgG, which represent our positive and negative controls and show the dynamic range of this measurement (Fig. 3D). We next tested target particles displaying only P-selectin and confirmed that it does indeed lead to macrophage binding (Fig. 3D). Finally, we measured binding to targets containing anti-biotin IgG, Fib5L bystander, and P-selectin over a range of concentrations. The results showed that the addition of P-selectin increased target binding to similar levels as the positive control condition. The maximum binding levels were reached with P-selectin at around 10% to 25%, which corresponds to a P-selectin surface density of 800–2000 µm^-2^. For context, these densities are on the same order of magnitude as estimated surface densities of P-selectin on activated platelets (∼450–750 µm^-2^), based on P-selectin surface expression^33–35^ and mean platelet diameter^36^.

After confirming that P-selectin increases adhesion between macrophages and target particles, we asked whether the presence of P-selectin also increased phagocytosis in the presence of bystander proteins by performing phagocytosis assays for each of the same conditions tested in the binding assay. Remarkably, we observed a significant increase in phagocytosis for conditions containing P-selectin alongside Fib5L bystander proteins (Fig. 3E). Maximum phagocytosis occurred when P-selectin accounted for 25% of the bystander proteins on the surface, which is consistent with maximum macrophage-particle adhesion in our binding assay. We observed phagocytosis reaching 70% of positive control levels—a 4-fold increase in phagocytosis compared to phagocytosis of targets containing anti-biotin IgG and Fib5L bystander proteins but no P-selectin. Phagocytosis sharply dropped off for P-selectin levels above 25%, presumably due to the added steric barrier of the long adhesions.

Importantly, coating target beads with P-selectin substantially increases cell binding compared with bare targets, nearly matching antibody-mediated binding (Fig. 3D, far right condition). Yet this ligand alone does not induce phagocytosis in our experiments (Fig. 3E, far right conditions). Phagocytosis is promoted only when an antibody is also present, which we consider to be kinetic enhancement.

To test whether these findings are true in primary macrophages, we reproduced bystander inhibition and phagocytic rescue using P-selectin in bone marrow–derived macrophages. As expected, primary-cell measurements were noisier than in RAW 264.7 cells, but the key effects held: Fib5L reduced uptake of opsonized beads by the bone marrow-derived macrophages, while adding 25% P-selectin restored phagocytosis (Supplementary Fig. 5). As with the RAW 264.7 cells, uptake of beads with only P-selectin did not differ significantly from bare beads, indicating no phagocytic signaling by P-selectin.

#### Tall binders enable short binders to overcome bystander-mediated inhibition in a minimal synthetic system

**Fig. 4:**
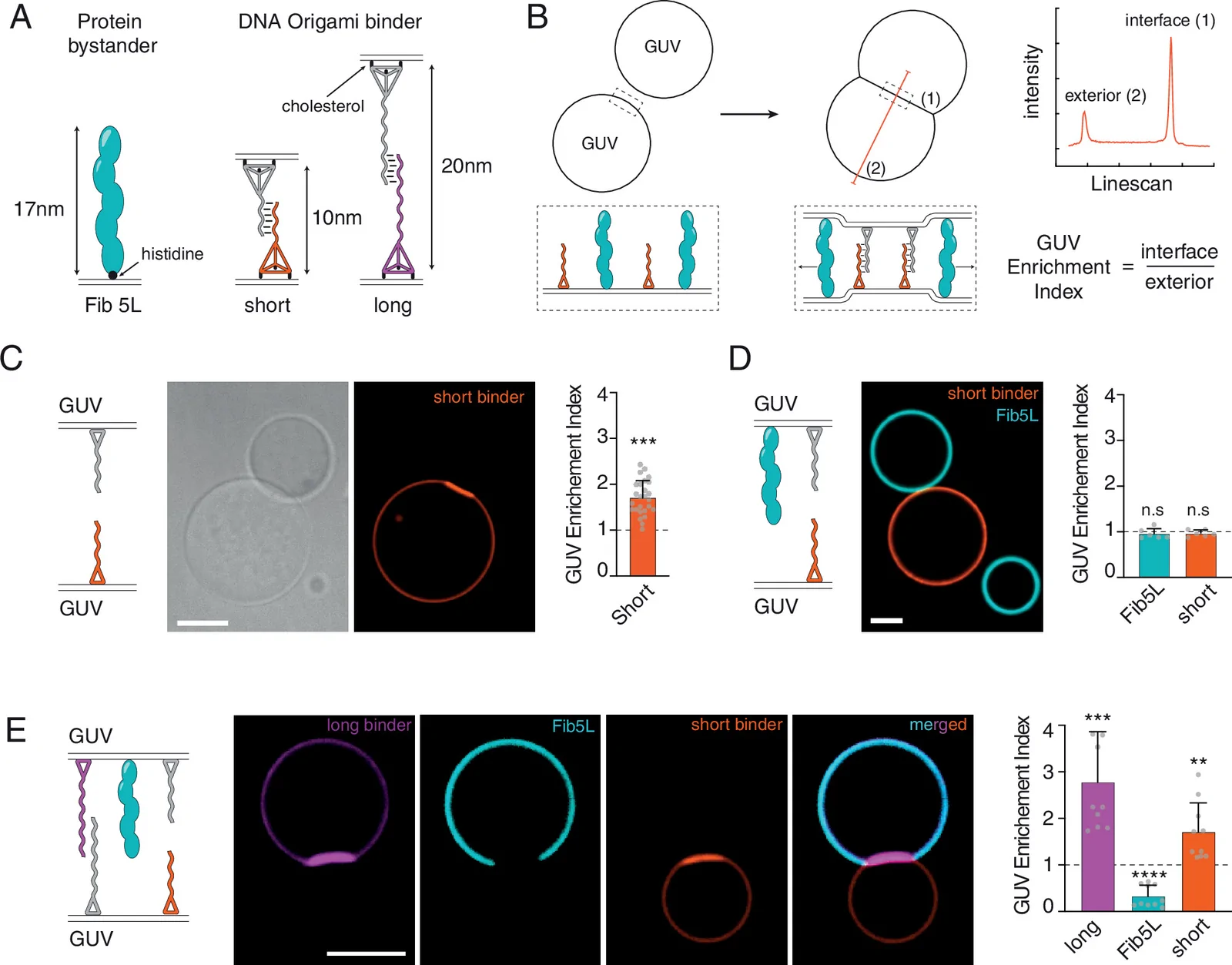
Tall binders help short binders overcome bystander barriers in reconstituted adhesion systems. **A** Purified bystander proteins (Fib5L, ~17 nm) and a complementary DNA origami binding pair (short binder pair, ~10 nm; long binder pair, ~20 nm) are used to test whether tall binders help short binders overcome bystander screening during membrane interface formation. **B** When GUVs containing DNA binders and bystander proteins interact, membrane interfaces form through DNA origami dimerization. Fib5L proteins attach to GUV membranes via His tags on DGS-NTA(Ni), while DNA binders attach through three cholesterol moieties at their base. Fluorescently labeled DNA binders and protein segregation or enrichment are visualized using fluorescence microscopy. Fluorescence intensity is measured from GUV contact line scans at both the interface (1) and the membrane exterior (2). The GUV enrichment index (GUV-EI) at the interface is calculated as the ratio of interface to exterior fluorescence. **C** GUV adhesion mediated by the short origami binder pair shows increased fluorescence at the interface. The enrichment index confirms short-binder enrichment (GUV-EI > 1; one-sample two-sided t-test vs the hypothetical value of 1, *p* < 0.0001), indicating successful binding. Scale bar: 10 µm. Bar: mean; Error bars: SD; *N* = 26 vesicle pairs. **D** With the short binder pair + Fib5L, no adhesion patches were observed, and both signals remained uniform (GUV-EI ≈ 1 for short binder and Fib5L; one-sample two-sided t-test vs 1: *p* = 0.32 and *p* = 0.41, respectively). Scale bar: 10 µm. Bar: mean; Error bars: SD; *N* = 6 vesicle pairs. **E** With Fib5L bystander present, GUVs containing both long and short binders formed adhesion patches with strong origami fluorescence at the interface. Both long and short binders were enriched (GUV-EI > 1; one-sample two-sided t-test vs 1: *p* = 0.0055 and *p* = 0.0006, respectively), while Fib5L was excluded (GUV-EI < 1; two-sided one-sample t-test vs 1: *p* < 0.0001), confirming successful overcoming of the bystander barrier. Scale bar: 10 µm. Bar: mean; Error bars: SD; *N* = 10 vesicle pairs.

Our results indicate that a tall binder, P-selectin, can play a crucial role in restoring phagocytosis in the presence of bystander proteins. To test whether this phenomenon results from equilibrium formation of a membrane interface in the absence of active cellular processes, we reconstituted a minimal membrane system that mimics the interaction between macrophages and target particles. Using giant unilamellar vesicles (GUV) functionalized with high-affinity short ligands (representing antibody-FcγR complexes), low-affinity tall ligands (mimicking P-selectin-PSGL1 complexes), and bystander proteins (Fig. 4A), we investigated whether bystander proteins could be excluded and close membrane contacts formed by the right combination of short and tall binders.

The bystanders consisted of the same Fib 5 L proteins that were fluorescently labeled at the N-terminus and anchored to vesicles via C-terminal His tags that associate with membrane Ni-NTA lipids. The short and long binders consisted of complementary DNA strands of two different lengths, mimicking ligand-receptor interaction through hybridization. These binders are anchored to GUVs via tetrahedral DNA origami, with three vertices carrying cholesterol anchors that insert into the bilayer, allowing stable anchoring of the binders on the vesicles. Each pair of binders contains a fluorescent marker for imaging. The long binder pairs have a maximum extension of approximately 20 nm, and the short ones approximately 10 nm^37^.

To quantify the distribution of molecules at interfaces, we measure fluorescence intensity across GUV pairs and calculate a GUV Enrichment Index (GUV-EI). This index is the ratio between the intensity at the interface (1) and the intensity at the free surface of vesicles (2) (Fig. 4B). A GUV-EI > 1 indicates enrichment of molecules at the interface, reflecting engagement of binders, a GUV-EI < 1 indicates their exclusion, and a GUV-EI ≈ 1 corresponds to a homogeneous distribution. When only short binder pairs decorate the surface of GUVs, we observe an increase in fluorescence at the interface (GUV-EI = 1.7 ± 0.4) (Fig. 4C), confirming effective engagement of complementary short binders.

However, when we add the bystander protein to the surface of one of the vesicles, we no longer observe enrichment of short binders at the contact point between vesicle pairs (Fig. 4D), resulting in an enrichment index close to 1 (GUV-EI = 0.97 ± 0.07). The Fib5L bystander proteins also remain homogeneously distributed (GUV-EI = 0.96 ± 0.11), with neither enrichment nor depletion at the contact point. This demonstrates the blocking role of Fib5L, consistent with its height (~17 nm), which is too large to allow engagement of the short binders.

We then tested whether adding long binders to the system (~20 nm) would overcome the Fib5L bystander barrier and promote short binder engagement, as we had observed in the case of macrophages and target particles decorated with P-selectin (Fig. 3). Consistent with the macrophage data, we observe a recovery of adhesion between vesicle pairs in the presence of long DNA binders, with enrichment of the short binders at the contact (GUV-EI = 1.4 ± 0.6) and exclusion of the bystanders (GUV-EI = 0.3 ± 0.2) (Fig. 4E). We found that the long binders are also enriched at the interface (GUV-EI = 2.7 ± 1), which may be due to the highly flexible single-stranded DNA region of the long binder that experiences a reduced entropic penalty compared to a tall binding protein and could remain at the membrane interface (Fig. 4A). This observation of bystander protein exclusion and short binder engagement in our simplified, cell-free GUV membrane interface with synthetic binders demonstrates that basic kinetic enhancement can arise without active cellular machinery. In living cells, additional processes such as actin-based protrusions could modify the energy landscape at cell-cell contacts.

### Theoretical modeling shows that short binder engagement in the presence of bystander proteins requires large membrane fluctuations, slowing synapse formation

To better understand how bystanders and tall adhesive proteins influence the formation of close contacts sustained by short binders like FcγR-IgG, we developed microscopic models that capture their fluctuating local concentrations and the varying shapes of their bound membranes. For short binders to engage, taller bystander molecules must be excluded from the contact zone. If the macrophage membrane were rigid, this would require complete removal of bystanders from the cell-target contact region ($A=1\,\mu {m}^{2}$); an unrealistic scenario given the prohibitively high activation energy barrier (${F}^{{{\ddagger}} }=200\,{k}_{B}T$) for even a low surface density of bystanders (${\rho }_{{bystander}}$ = $200\,\mu {m}^{-2}$).

**Fig. 5:**
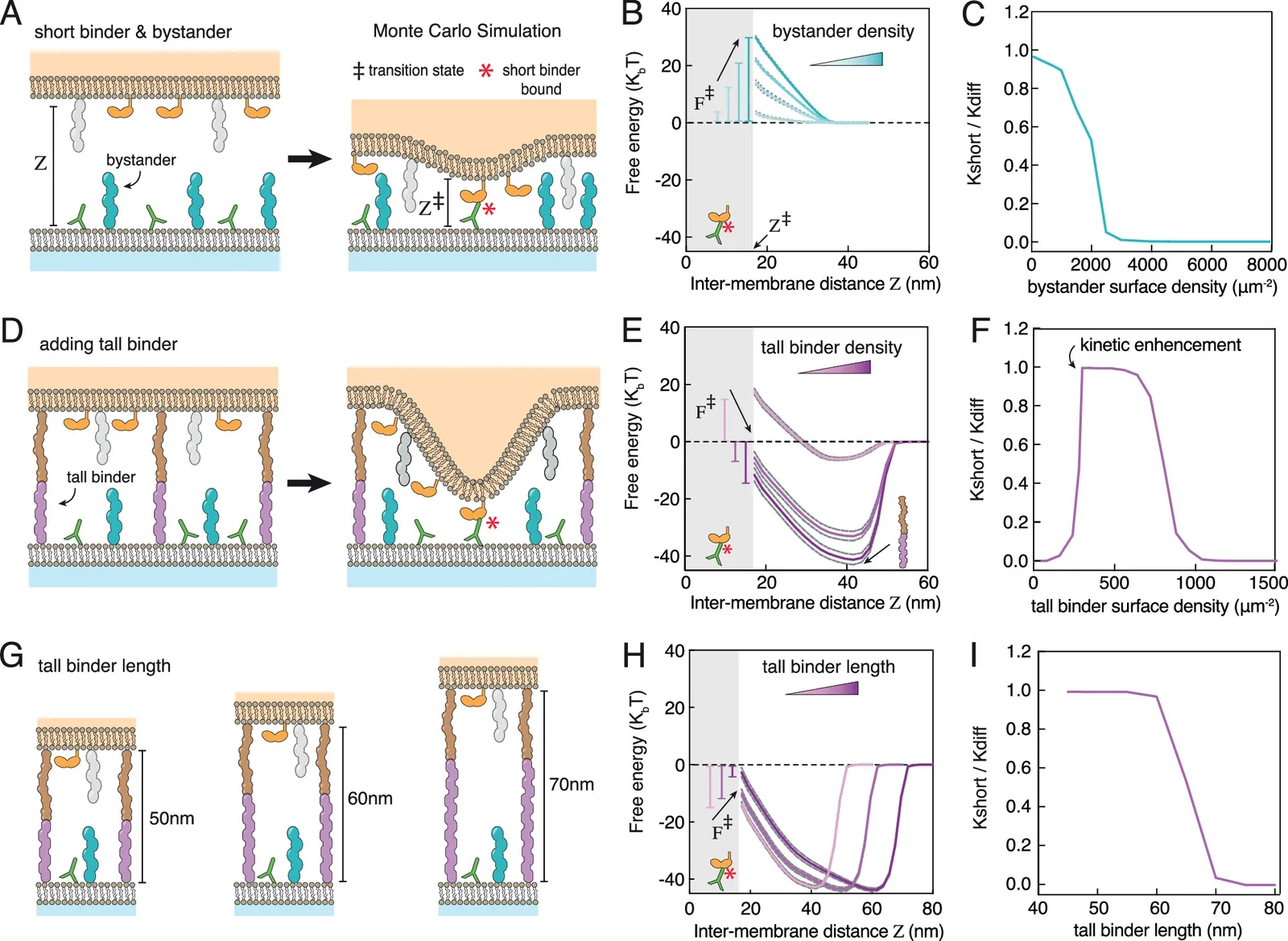
Monte Carlo simulations show blocking of short binder engagement by bystander molecules and recovery of short binder engagement with tall binders. **A** Proposed mechanism shows how short proteins bind between a macrophage and target surface by displacing taller bystander proteins from the contact zone thanks to membrane deformation. The transition state exhibits a dimple-like distortion that brings the two membranes locally within the reach *Z*^‡^ of the short binder, reducing the thermodynamic cost of bystander exclusion. **B** Monte Carlo (MC) simulation of the interface enables calculation of the system’s free energy and confirms that increasing bystander molecule concentration raises the transition state free energy (*F*^‡^), impeding the engagement of short binders. Solid lines show the mean of *N* = 3 independent simulations with different initial seeds. Gray dashed lines indicate the SEM; if they are not visible, the SEM is smaller than the line width. **C** Increasing the bystander density raises the energetic barrier to short binder engagement, as shown by reduced kinetic rate of short binder engagement (*K*_*short*_) compared to the diffusion-limited rate constant in bystander-free conditions (*K*_*diff*_). **D** Tall binders that favorably bind across the membrane gap alter this pathway by stabilizing an intermediate membrane separation state. A larger dimple is required for short binder engagement and excluding both bystanders and tall proteins. **E** MC simulations of the interface reveal that the presence of tall binders (tall binder densities 80 µm^−2^, 320 µm^−2^ and 400 µm^−2^) creates an intermediate binding state that promote short binder binder engagement. Solid lines show the mean of *N* = 3 independent simulations with different initial seeds. Gray dashed lines indicate the SEM; if they are not visible, the SEM is smaller than the line width. F Beyond a critical concentration of tall binder, the engagement of short binders is kinetically enhanced, approaching diffusion-limited rates (*K*_*diff*_). However, if the tall binder concentration is too high, excessive stabilization of the intermediate binding state blocks synapse formation kinetics. **G** Longer tall binders increase the inter-membrane distance of the intermediate state. **H** This raises the transition-state free energy (*F*^‡^), as they require more extensive membrane deformation for short binder engagement. Solid lines show the mean of *N* = 3 independent simulations with different initial seeds. Gray dashed lines indicate the SEM; if they are not visible, the SEM is smaller than the line width. **I** The corresponding engagement rate constant shows that synapse formation is not kinetically enhanced for tall binders of sizes longer than 60 nm. For parameters used in the MC simulations, see Supplementary Table 1.

Thus, we propose that fluctuating local protein abundances and membrane flexibility are key features of phagocytic synapses and cell-cell contacts. Specifically, we present a simple physical scenario involving a transition state that requires deformation of the macrophage membrane—formation of a dimple—allowing short binder engagement to nucleate within a small area with low local abundance of bystander molecules (Fig. 5A). This bypasses the large energetic barrier of total bystander exclusion from the entire macrophage-target contact region prior to establishing strong favorable interactions between short binders, as also proposed for c-SMAC^38^.

For an interface containing only short binder pairs and bystanders, the energy required for dimple formation, ${F}_{{deform}}\propto \, \varDelta {h}^{{{\ddagger}} }\sqrt{{\kappa }_{{bend}}{w}_{{evac}}}$, depends on three factors: the deformation’s vertical scale $\varDelta {h}^{{{\ddagger}} }$, the membrane’s bending rigidity ${\kappa }_{{bend}}$, and the reversible work per unit area ${w}_{{evac}}={\rho }_{{bystander}}{k}_{B}T$ needed to evacuate bystanders from a membrane region (see Supplementary Notes, Section 1). From this theory, we can draw several conclusions about the activation energy: it increases with (i) higher bystander density, (ii) greater deformation vertical scale, and (iii) increased membrane bending rigidity.

To further verify the basic features of these estimates, we performed Monte Carlo simulations of a microscopic model of macrophage and target membranes that includes short binders and bystander molecules (see SI text, Section 2). From these simulations, we computed the energy profile of interface formation as a function of *r*, the intermembrane distance, for different densities of bystander molecules. We found that the free energy barrier ${F}^{{{\ddagger}} }$ indeed increases as the bystander density increases (Fig. 5B). The increase in this energy barrier causes a kinetic slowdown in short binder engagement (Fig. 5C), which is consistent with the experimental results in Fig. 1. Interestingly, varying the affinity of short-binder pairs does not affect this slowdown (Supplementary Fig. 6C), suggesting that affinity mainly stabilizes the final close-contact state with little effect on the barrier-crossing step.

### Monte Carlo simulations suggest that specific densities of tall ligands help overcome barriers created by bystander molecules

We then investigated the effect of adding tall binder molecules on the kinetics of short binder engagement. We consider a population of tall binders (like the PSEL-PGSL1 pair) that can bind across the macrophage-target interface with sufficient strength to generate a metastable intermediate state at a membrane separation greater than the bystander proteins’ height (Fig. 5D). Introducing such an intermediate, with free energy ${F}_{{{\mathrm{int}}}} < 0$ and without lowering the maximum free energy ${F}^{{{\ddagger}} }$ along a barrier-crossing pathway, generically slows barrier-traversing kinetics. To enhance the kinetics of short-binder engagement, as observed in experiments, tall binders must stabilize the transition state. The schematic progression in Fig. 5D illustrates how adhesion of a tall binder could do so. Here, membrane deformation begins from the intermediate membrane separation *h*, requiring a reversible work ${F}_{{deform}}^{{{\ddagger}} }\approx \alpha {\varDelta h}^{{{\ddagger}} }\sqrt{{{\kappa }_{{bend}}w}_{{evac}}}$ to initiate receptor engagement. The modified deformation scale ${\varDelta h}^{{{\ddagger}} }$ and evacuation free energy per unit area ${w}_{{evac}}$ are both *larger* than the corresponding values for bystander proteins alone, so that the cost of deformation is actually magnified by the presence of tall binders. Importantly, however, the free energy maximum ${F}^{{{\ddagger}} }={{F}_{{deform}}^{{{\ddagger}} }+F}_{{{\mathrm{int}}}}$, can be lower than in the absence of tall binders (see Supplementary notes, Section 1).

To study this effect, we performed a Monte Carlo simulation of membrane interfaces in the presence of tall ligands. The corresponding free energy profiles as a function of intermembrane space *r* show an intermediate minimum near the height of the tall ligand complex (Fig. 5E). Increasing the tall ligand density lowers the free energy of the intermediate state and decreases the free energy barrier ${F}^{{{\ddagger}} }$. The kinetic rate of short binder engagement (*k*_*short*_) shows three distinct regimes as the density of long binders increases (Fig. 5F). At low tall binder concentrations, stable intermediate states cannot form, so long binders have no effect on short binder engagement kinetic rate. Above a certain density threshold, tall ligand binding kinetically enhances synapse formation to reach uninhibited kinetic rates. This enhancement plateaus as concentrations become too high, at which point the intermediate state becomes overly stable, resulting in a collapse of the kinetic rates. The extent of the kinetically enhanced regime can be further modulated by varying the relative density of the tall binder pairs on both macrophage and target membranes (Supplementary Fig. 6AB). Increasing the short-binder affinity slightly widens the kinetic enhancement range (Supplementary Fig. 6D), suggesting that higher-affinity receptor engagement could further extend this regime. The three distinct regimes as a function of tall binder density observed in the simulations align with our experimental results showing that the phagocytic influence of P-selectin density is non-monotonic and has an optimum (Fig. 3).

Finally, we investigated how varying the length of tall binders would affect the transition-state free energy and the subsequent kinetic rate of short-binder engagement. We performed a Monte Carlo simulation with tall binders of different lengths by varying the height of the tall binder on the target membrane, keeping all other molecular heights constant, and computed the resulting free energy profiles (Fig. 5G, H). With increasing length of tall binders, more severe membrane deformation is required to reach the transition state, yielding higher free energy barriers ${F}^{{{\ddagger}} }$ but only slight changes in stability of the intermediate state. This results in a reduction in the kinetic rate of short-binder engagement (Fig. 5I), indicating that there may be an optimal height for tall binders that is only slightly taller than the bystander proteins.

## Discussion

The crowded environment of cell surfaces can hamper the formation of close cell-cell contacts. This is a particular challenge for macrophages, where antibodies targeting short antigens (<10 nm) are known to promote phagocytosis better than those against tall antigens (>10 nm) because close membrane contact is needed to exclude the inhibitory phosphatase CD45 and trigger phagocytosis^8^. How can short Fcγ receptors on macrophages overcome surface crowding and reach antibodies on short antigens that are hidden by surrounding taller bystander proteins? Here we demonstrate experimentally, theoretically, and through simulations that tall adhesive proteins at macrophage phagocytic synapses can promote antibody binding in the presence of bystander molecules that would otherwise inhibit phagocytosis.

Starting with antibody-coated target particles that are robustly phagocytosed, we show that addition of non-binding fibronectin domain repeat proteins can reduce phagocytosis by blocking Fcγ receptor engagement. Imaging of interface formation on supported lipid bilayers confirms that IgG-FcγR clustering is reduced and slowed as bystander protein density and height is increased. Surprisingly, we find that addition of another tall protein to the target particle—P-selectin, which binds the tall receptor PSGL-1 on macrophages—can restore phagocytic efficiency at low surface densities. This effect is only observed for a limited range of low P-selectin surface densities, as higher surface densities block phagocytosis. This suggests that while additional tall adhesive proteins will increase the barrier to close contact, certain surface densities of tall binders can enhance the kinetics of IgG-FcγR binding and subsequent phagocytosis. To further test this idea, we reconstituted membrane interface formation in the presence of bystander proteins using GUVs together with DNA-based short and long binders. Consistent with our macrophage findings, we observed that tall binders could restore bystander protein exclusion and short binder engagement.

Theory and computer simulations suggest a generic physical mechanism for the observed kinetic enhancement of close membrane contact, in which long adhesive molecules mitigate the reduced kinetics of short binder engagement caused by taller bystander molecules. Simply stabilizing an intermediate state where the membranes are held together—but not in close contact—is not sufficient to explain the observed change in kinetics. While this might increase the chances of surface encounters, the process would remain inefficient since the system must still overcome a barrier to escape this intermediate state and achieve close contact. We argue that the intermediate state must involve deformation of the macrophage and/or target surfaces, thereby allowing short adhesive molecules to bind while gradually excluding bystander proteins. Adding tall binders actually increases the cost of this deformation, but the transition state retains a large number of adhesive contacts. It is therefore likely that factors controlling membrane deformation play a key role in regulating kinetic enhancement, whether through the amplitude of passive thermal fluctuations of the membrane or through deformations generated by actin-based protrusions. However, it is difficult to directly isolate actin’s contribution to the kinetic enhancement mechanism, since inhibiting actin activity also abolishes phagocytosis. It would therefore be very interesting in future studies to characterize how reducing or amplifying these deformations affects the efficiency of kinetic enhancement.

We show that a net decrease in energy barrier height due to tall binders increases the rate of short-binder engagement at low concentration and/or binding strength of the tall binders. However, with extensive and/or strong adhesion, escaping the proximal intermediate state becomes the limiting factor. In this regime tall binders instead inhibit the kinetics of short binder engagement. In the case of macrophages, since the intermediate state stability increases with the cell’s large footprint, the shift from enhancement to inhibition occurs at relatively low levels of adhesion, consistent with our experimental measurements. Our computational model was developed to validate the mechanistic principles observed in macrophage phagocytosis.

This Monte Carlo model successfully captured the experimental trends observed in our macrophage experiments. Nevertheless, it relies on simplifying assumptions (e.g. rigid rods on a lattice) that limit its quantitative applicability for flexible binders. In particular, the behavior of the long DNA binder in Fig. 4 lies outside the scope of the current model because, once short binders engage, the long binder’s flexibility allows it to bend and coexist at the interface (Fig. 4E), whereas a rigid-rod model would predict exclusion. Because of their footprint, long binders that are not excluded from the interface could sterically hinder access to other molecules, thereby reducing the effective enrichment of short binders. Therefore, flexibility-induced long/short-binder coexistence complicates the interface-formation model. Future work will incorporate ligand flexibility and predict how differences in measurable flexibility^39^ may modulate kinetic enhancement.

Given that crowded membranes and multiple potential binders are often present at cell-cell interfaces, bystander-mediated blocking and kinetic enhancement by tall binders may be under-appreciated phenomena that occur in many natural systems. Efficient T-cell activation is known to require the binding of multiple co-receptors in addition to the TCR, including CD2, CD28, and the relatively tall integrin LFA-1, which binds to ICAM-1 on the target cell^40,41^. This leads us to speculate that a secondary role of some co-receptors may be to provide kinetic enhancement of TCR interface formation, as P-selectin does in our experiments on macrophage ADCP. Similarly, antibody-dependent cellular cytotoxicity (ADCC) by Natural Killer cells, known to be an important contributor to cancer cell clearance during immunotherapy^42^, relies on FcγR binding to IgG opsonized antigens on the target cell surface and is likely affected by bystander proteins.

Our observation of the kinetic enhancement of ADCP mediated by tall binding proteins could potentially be utilized to improve existing antibody-based immunotherapies. In cases where an antibody is targeted to a short tumor antigen, kinetic enhancement can be achieved by forming a temporary interface between long molecules. Molecules with the ability to bind surface proteins on two different cells, as has been previously reported with bispecific antibodies^43^ and bispecific T-cell Engagers (BiTE)^44^, could be used in combination with traditional immunotherapeutic antibodies that trigger ADCP and ADCC. We speculate that a “Big‑BiTE” therapeutic could be engineered to behave like a tall binder pair, leaving a gap only slightly larger than the average height of bystander proteins. In principle, such geometry could bias engagement toward relatively short immune receptors at cell–cell contact, thereby enhancing immune effector function.

We expect the need to mitigate kinetic frustrations imposed by bystander proteins to arise in many settings. Re-engineering transition states with long adhesive molecules is a simple strategy for doing so. Given the sensitivities to adhesion strength and distance we have observed, one can imagine not only rescuing efficient receptor engagement but also modulating it precisely in space and time, efficiently discriminating among receptor pairs according to their height. Realizing such control in engineered cell systems and exploring the implications in natural biological contexts are exciting directions for future work.

## Methods

### RAW 264.7 cell culture

RAW264.7 macrophage-like cells were obtained from the UC Berkeley Cell Culture Facility. The cells were cultured in RPMI medium (Corning) supplemented with penicillin-streptomycin (1%) and fetal bovine serum (10%). Cells were passaged at a ratio of 1:5 every two days. After 2 months of passaging, they were replaced with a new batch of low-passage cells to prevent phenotypic drift.

### Bone marrow-derived macrophage cell culture

Bone marrow–derived macrophages (BMDM) from male C57BL/6 (B6) mice were kindly provided by the Robey Lab (UC Berkeley). Mice were bred and maintained under pathogen-free conditions in accordance with guidelines approved by the Institutional Animal Care and Use Committees at the University of California, Berkeley. Wildtype (C57BL/6, Stock. No. 000664) mice were purchased from the Jackson Laboratory. For the collection of bone marrow, mice were euthanized with isoflurane followed by cervical dislocation. From dissected tibia and femur bones, bone marrow was collected by centrifugation (1000 × *g*, 15 seconds) and red blood cells were lysed using ACK lysis buffer. Bone marrow cells were subsequently differentiated into macrophages and cultured in bone marrow culture medium (DMEM, 10% FBS, 1% Penicillin-Streptomycin, and 1 ng/mL recombinant mouse CSF-1 (Gibco)) at 37 °C and 5% CO2 for 4 days. Adherent cells with 1−2 protrusions showing at low magnification were kept and frozen for further use. Cells were maintained in RPMI 1640 supplemented with 10% heat-inactivated FBS and 1% penicillin–streptomycin at 37 °C in 5% CO2. Experiments were performed using BMDMs within 24 h after thawing.

### Fibcon family protein expression and purification

Fibcon proteins (Fib1L, Fib3L, Fib5L) were expressed in Rosetta DE3 competent E. coli cells (EMD Millipore). After transformation with the plasmid, cells were grown at 37 °C until they reached an OD of 0.8, at which point they were induced with 0.3 mM IPTG and incubated overnight at 18 °C. Cells were harvested and resuspended in PBS with 0.5 mM TCEP and 10 mM imidazole, followed by cell lysis via sonication. The lysate was clarified by centrifugation at 20,000 g for 45 min. Protein was purified by loading onto a His-Trap HP column (GE Healthcare) and eluting with an imidazole gradient on an AKTA Pure (GE Healthcare) chromatography system. Protein peak fractions were further purified and buffer-exchanged into PBS via gel chromatography on a Superdex 200 column (GE Healthcare). TCEP (0.5 mM) was added to each protein solution to reduce disulfide bonds, and the proteins were flash-frozen and stored in small aliquots.

### Preparation of Supported Lipid Bilayers

Planar supported lipid bilayers (SLB) were prepared by incubating 50 µL SUV solution and 50 µL MOPS buffer (25 mM MOPS, 125 mM NaCl, pH 7.4) in a PDMS chamber with RCA-cleaned glass coverslips (REF 48393−230 24x40mm N 1.5 976 Avantor). After 20 min, the well was washed 5× with PBS to wash away excess SUVs while ensuring that the chamber did not dry out. Fibcon proteins and anti-biotin IgG were added to the well at respective concentrations of 50 nM and 125 ng/mL and incubated for 20 min. Finally, the well was washed 5× with PBS to remove excess Fibcon proteins and IgG.

### Reconstitution of target particles

Small Unilamellar Vesicles (SUV) were made by mixing lipids dissolved in chloroform, followed by desiccation, rehydration, and sonication. Lipid bilayers coating reconstituted target particles contained the following: 0.5% Biotin PE (Avanti, A87282), 0.4% 647 DOPE (milipore Sigma, 42247), 0.5%, 1.6%, 2.8% or 5% DGS-NTA(Ni) (Avanti, Cat: A89404), and the remainder POPC (Avanti, A80557). For measurements of Fibcon densities, 647 DOPE was left out so that it would not interfere with the fluorescence signal of the fluorescently labeled Fibcon proteins. During protein conjugation steps, Fibcon proteins were incubated with lipid-coated target particles at a concentration of 50 nM and anti-biotin IgG at a concentration of 125 ng/mL. For experiments in which P-selectin was displayed on target surfaces alongside Fibcon proteins and anti-biotin IgG, His-tagged mouse P-selectin (Sino Biological, 50737-M08H) and Fibcon were added at different ratios, while keeping the total protein concentration fixed at 50 nM.

### Measuring protein densities on target particles

Fib3L proteins were labeled with Alexa Fluor 647 at a single C-terminal cysteine labeling site using Alexa Fluor 647 C2 Maleimide (Thermo Fisher Scientific, Invitrogen, A20347). Reconstituted target particles were created using the same lipid compositions as used in the phagocytosis assays, but without any fluorescent 647 DOPE lipid. Just as in the phagocytosis assays, targets with different fractions of DGS-NTA(Ni) lipids were used to display different densities of Fibcon proteins. Specifically, targets with DGS-NTA(Ni) fractions of 0.5%, 1.6%, 2.8% and 5% were made. Fluorescent Fib3L proteins were used to coat the particles at the typical concentration of 50 nM, along with 125 ng/mL anti-biotin IgG. These target particles were analyzed for Fib3L fluorescence using flow cytometry, and the average fluorescence for each condition was recorded with at least 10,000 particles measured per condition. Using the same instrument settings, calibrated Alexa Fluor 647 MESF beads (Bangs Laboratories, Cat. #488 A) were measured to create a conversion between fluorescence intensity signal and absolute fluorophore counts (Supplementary Fig. 1). Fluorophore densities were determined by taking the total number of fluorophores measured on each condition of particles and dividing by the surface area of the 4 µm beads. We assumed the fluorophore density was equal to the Fibcon protein density since each Fibcon protein carried a single fluorophore.

### Assaying target particle binding

Reconstituted target particles were made in the same way described above (see “Reconstitution of target particles”) and in ref. ^20^. RAW264.7 cells were labeled with CellTracker Green CMFDA dye (ThermoFisher Scientific, Invitrogen, C7025) and washed twice with PBS, followed by final resuspension in cell medium. Cells were diluted to a concentration of 350 cells/µL, and 100 µL of cells were added to each tube. 100 µL of target particle suspension containing ~500,000 particles was added to each tube, and the tubes were incubated for 30 min at 4 °C to prevent phagocytosis and under constant rotation to prevent settling. After 30 min, each tube was run through a flow cytometer (ThermoFisher Attune NxT Acoustic Focusing Cytometer equipped with 405, 488, 561, and 637 nm lasers) and fluorescence data for at least 5000 macrophages were collected. All tubes were kept on ice prior to loading into the flow cytometer, and the entire run time was kept under 30 seconds to ensure that bound particles were not phagocytosed as the sample warmed to room temperature. A low flow rate was used to prevent unnecessary shearing of bound targets from cells. Results were quantified by plotting particle fluorescence (647-DOPE) vs. macrophage fluorescence (CellTracker green CMFDA). Particle fluorescence (647-DOPE) was excited using the 637 nm laser and detected in the RL1 channel using a 670/14 nm bandpass filter. Macrophage fluorescence was excited using the 488 nm laser and detected in the BL1 channel through a 530/30 nm bandpass filter. The fraction of macrophages with targets bound to their surface was determined by dividing the number of macrophages with targets bound to their surface (647-DOPE + , CMFDA + ) by the total number of macrophages (CMFDA + ). Thresholds for classifying events as target particles, macrophages alone, or macrophages bound to target particles are depicted in Fig. 3C.

### Assaying phagocytosis

All phagocytosis assays were performed in accordance with the steps outlined in our previously published protocol^20^. Briefly, reconstituted target particles were made by coating 4 µm diameter glass beads (Bangs Laboratories, Cat. #SS05002) with a fluorescently labeled fluid lipid bilayer. His-tagged proteins were coupled to the bilayer via interaction with DGS-NTA(Ni) lipids in the bilayer. Anti-biotin IgG (eBioscience, clone BK-1/39, Alexa Fluor 488 labeled) was targeted to biotinylated lipids in the bilayer. Reconstituted target particles were incubated with RAW 264.7 macrophage-like cells in a 96-well plate for 30 min at 37 °C, after which excess beads were washed from the wells, and CellTracker Green CMFDA dye was added to the wells to fluorescently label the cells. Phagocytosis was quantified by capturing fluorescence images of cells and targets using wide-field fluorescence microscopy (Nikon Ti-2 microscope, Nikon Sola illumination, Nikon SR Plan Apo 20X/0.75 objective). Fields of view were selected by randomly assigning non-overlapping positions within each well. Fields were excluded only for predefined QC failures (e.g., out of focus, saturation, or major artifacts/well contamination). Exposure time and illumination intensity were kept constant throughout the experiment. The images were then analyzed using an automated CellProfiler (Broad Institute V4.2.8) analysis pipeline. The phagocytic index for each condition was determined by measuring the average internalized target particle lipid fluorescence intensity per cell using the CellTracker Green CMFDA dye for masking. All experiments were repeated at least 3 times on 3 separate days. To obtain final phagocytic indices with error, each daily phagocytic index was normalized to the average across all conditions, yielding a normalized phagocytic index that accounts for day-to-day global variations in phagocytosis. Error bars represent standard deviation across all replicates of a given condition.

### TIRF imaging for IgG enrichment measurement

Live cell imaging was performed using a stage-top incubation chamber (Okolab) to ensure 37 °C temperatures and 5% CO2. For time-lapse image acquisitions, macrophages were dropped onto SLBs inside the incubation chamber and immediately imaged. Micro-manager software was used to automatically control the microscope (Nikon ECLIPSE Ti-2 inverted microscope, objectives: SR Plan Apo 20X/0.75, SR Plan Apo IR 60X/ 1.27 WI, Apo TIRF 60×1.49NA Oil objective (Nikon Instruments Inc.)) and capture both TIRF and RICM images every 30seconds for multiple fields of view. Fields of view were selected by randomly assigning non-overlapping positions within each well. For endpoint image acquisitions, macrophages were incubated on SLBs in the stage-top incubator for 30 min prior to imaging.

### IgG enrichment analysis

Images collected from TIRF imaging were analyzed using Fiji (V: 2.16.0/154p). Individual cells were identified in the RICM channel of each image. A mask around the boundary of each cell was drawn, and the mask was applied to the corresponding TIRF image. The average fluorescence intensity from Alexa Fluor 488-labeled anti-biotin IgG within each cell mask was measured, and the enrichment index (EI) was determined by dividing each value by the average background fluorescence intensity of the same field of view. Each of these experiments was repeated at least 3 times on 3 separate days. Data points from all individual experiments were combined for each condition to comprise the final data.

### Giant unilamellar vesicles formation

For all experiments, we used a consistent lipid mixture to form Giant Unilamellar Vesicles (GUV) membranes (POPC (Avanti, SKU:850457 C) 74.5%; PEG2K-PE (Avanti, SKU:880120 P) 0.5%; Cholesterol (Avanti, SKU:700000P-1G) 20%; DSG-NTA(Ni) (Avanti, Cat: A89404) 5%) when the blocker Fib5L should be anchored to the GUVs, and (POPC 79.5%; Peg2K-PE 0.5%; Cholesterol 20%) otherwise. To prepare GUVs, we spread 0.25 mg of the lipid mixture uniformly on indium tin oxide (ITO)-coated slides. After vacuum drying for >30 min to remove chloroform, we assembled a capacitor using two lipid-coated slides as electrodes separated by a 0.3-mm rubber isolator septum. We filled the chamber with ~200 μL of 350 mM sucrose solution (~350 mOsm). GUVs (10–100 μm diameter) were formed by electroformation, applying 1.5 V AC at 10 Hz for 1 hour to the capacitor, following established protocols^45^.

### Formation of DNA tetrahedra (short and long binder)

To assemble the DNA tetrahedron binder, we combined 0.5 µL of each oligonucleotide building block (Tet-B, Tet-C, Tet-D, and the variable strand) in PCR tubes, added 48 µL of 1× PBS, vortexed, and placed the mixture in a thermal cycler. The thermal cycling program ran for 90 min through the following steps: initial hold at 20 °C, heating to 90°C and holding for 10 min, cooling at -1.5 °C/minute until reaching 20°C, and finally holding at 4 °C. The final concentration of the origami tetrahedron in the solution is 1 µM (Supplementary Fig. 7). The binding domain of the synthetic origami binder has the same length (12 nucleotides) for both tall and short variants, but the short binder is designed with a stronger binding domain, having 80% GC content compared to the tall binder’s 25% GC content. See Supplementary Table 2 for the complete sequences used to assemble the origami binders.

### GUV functionalization with short and long DNA binders and/or Fib5L blocker molecules

The origami stock solutions are diluted in PBS 1X to obtain 100 µL of 500 nM solution in an Eppendorf tube containing 50 nM of Fib5L blockers. 5 µL of GUV solution (with 5% nickel lipids when Fib5L blocker needs to be anchored) are added to this solution, gently homogenized using a micropipette, then incubated on the rotisserie (Thermo Scientific, Model M90615, American Laboratory Trading) for 5 min. To eliminate excess origami (and/or Fib5L), the tube is placed vertically to allow GUV sedimentation. Then, 10 µL of supernatant are collected and mixed with 90 µL of PBS; this washing step is repeated twice. The purified solution is maintained on the rotisserie to prevent sedimentation of functionalized GUVs until use.

### Formation of GUV-GUV pairs

Once the surfaces of both complementary GUV types are decorated with the necessary origami binders and protein blockers, they are brought into contact by mixing 5 µL of each complementary GUV solution in 100 µL of PBS, then incubated for 10 min on a rotisserie to prevent sedimentation. The mixture is deposited on a coverslip previously passivated by incubation for 1 minute with 10% BSA, then rinsed 3 times with buffer (PBS), to prevent vesicle rupture.

### Imaging and measurement of enrichment index at GUV-GUV interface

The formed GUV-GUV pairs are then imaged in their equatorial plane on a Nikon Ti-2 microscope equipped with a spinning-disk unit (Yokogawa CSU-X1) through an SR plan Apo IR 60X/1.27 WI objective. The fluorescence intensity of the different molecules present (DNA origami binder and Fib5L blockers) is measured by a linescan and allows calculation of an enrichment index at the contact between vesicles (GUV-EI) by taking the ratio of fluorescence intensity at the contact normalized by that outside the contact^46^.

### Theory overview

The theoretical framework developed in this work models the interaction between macrophages and target particles, focusing on key “dimple” formation in the macrophage membrane during receptor engagement. The model quantifies the energetic cost of membrane deformation using Helfrich’s small-gradient approximation, accounting for both elastic energy and the thermodynamic cost of evacuating bystander proteins from the contact zone. The kinetics of receptor engagement are described using overdamped Langevin dynamics, with the separation vector r between a macrophage and a target particle treated as a reaction coordinate. The steady-state solution to the resulting Smoluchowski equation, with appropriate boundary conditions, yields phagocytosis rates relative to the diffusion-limited case. This framework particularly elucidates how tall adhesive proteins can mitigate the kinetic effects of bystander molecules through barrier reduction. See the Supplementary notes for a full description of the theory framework.

### Monte Carlo simulations overview

Simulations were performed to investigate microscopic fluctuations in bystander-hindered phagocytosis, using a model adapted from^47^. The model represents macrophage and target-membrane topography on a discrete lattice, with membrane-bound proteins treated as noninteracting rigid, rod-like molecules that remain oriented parallel to the lattice normal. Their orientation, therefore, fluctuates as the lattice deforms. The system consists of two two-dimensional lattices representing the macrophage and target membranes, with surface-anchored molecules – short binder and tall binder representing, respectively, FcγR/IgG or P-selectin/PSGL-1, and blocker molecules representing the macrophage and target bystander proteins – occupying sites subject to spatial and energetic constraints. The simulation employed a grand canonical ensemble approach, in which protein population fluctuations were integrated out, yielding a free-energy function governing membrane separation. Changes in membrane height were proposed at random lattice sites and accepted according to the Metropolis criterion, accounting for membrane elastic energy, protein-mediated free energy changes, and a biasing potential. Free energy profiles were computed along a membrane gap coordinate (Z) using umbrella sampling. The simulation was performed on a 0.7 μm² elastic sheet discretized into a 70 × 70 grid of 12 × 12 nm² patches. The system was equilibrated for 2 × 10⁵ Monte Carlo sweeps, before data were collected from an additional number of MC sweeps between 8 × 10⁵ and 2 × 10⁶. Refer to Supplementary Notes Section 2 for a full description of the Monte Carlo simulations.

### Antibodies

Biotin was bound by anti-biotin IgG (eBioscience, clone BK-1/39, Alexa Fluor 488 label, Catalog # 53-9895-82) with a 1:4000 dilution in PBS buffer. This antibody, used to opsonize glass beads, was purchased from commercial sources and used without further labeling or purification.

### Statistics & reproducibility

Results are reported as mean ± s.d. from independent replicates (biological or technical, as specified in the relevant figure legends) for a representative experiment, unless otherwise indicated. Differences between two groups were assessed using Student’s t-test, unless otherwise indicated. Analyses were carried out in Prism 11 Version 11.0.1 (99). All collected data points were included in the analysis. No statistical method was used to predetermine sample size. No data were excluded from the analysis. The experiments were not randomized. The Investigators were not blinded to allocation during experiments and outcome assessment. The strategy for field-of-view selection is described in the microscopy analysis sections of the Methods: “Assaying phagocytosis” and “TIRF imaging for IgG enrichment measurement”.

### Reporting summary

Further information on research design is available in the Nature Portfolio Reporting Summary linked to this article.

## Supplementary information

Supplementary Information

Transparent Peer Review file

Reporting Summary

## Source data

Source Data

## Data Availability

All data are included in the Supplementary Information or available from the authors, as are unique reagents used in this Article. The raw numbers for charts and graphs are available in the Source Data file whenever possible. Source data are provided with this paper.

## Code availability

The code used for the Monte Carlo simulations in this study is available at the following GitHub repository: https://github.com/fletchlab-git/Overcoming-Steric-Inhibition-with-Tall-Adhesions.
