# Supplementary material for: Tall adhesions overcome cell surface steric interference and rescue antibody-dependent phagocytosis in macrophages
Source: Nat Commun. 2026 Sep 28;17:11213. doi: 10.1038/s41467-026-78095-7 (PMC13720194; doi:10.1038/s41467-026-78095-7)
Supplement: Supplementary file 1 [file 41467_2026_78095_MOESM1_ESM.pdf]

Supplementary Information for

Tall adhesions overcome cell surface steric interference and rescue antibody-dependent phagocytosis in macrophages

Aymeric Chorlay[*1], Aaron M. Joffe[*1,2], Jared Huzar[3], Jaffar Hasnain[4], Caitlin E. Cornell[1], Phillip L. Geissler[4,5,8], and Daniel A. Fletcher[1,2,3,6,7#]

[1]Department of Bioengineering, University of California, Berkeley, CA 94720, USA

[2]UC Berkeley/UC San Francisco Graduate Group in Bioengineering, Berkeley, CA, USA

[3]Biophysics Graduate Group, University of California, Berkeley, Berkeley, California 94720, United States

[4]Department of Chemistry, University of California, Berkeley, Berkeley, CA, USA

[5]Chemical Sciences Division, Lawrence Berkeley National Laboratory, Berkeley, CA, USA

[6]Biological Systems and Engineering Division, Lawrence Berkeley National Laboratory, Berkeley, CA, USA

[7]Chan Zuckerberg Biohub, San Francisco, CA, USA

[8]Deceased author

[*] These authors contributed equally.

# Corresponding author: fletch@berkeley.edu

This PDF file includes:

Supplementary Tables 1-3
Supplementary Figs. 1-9

Supplementary Figures

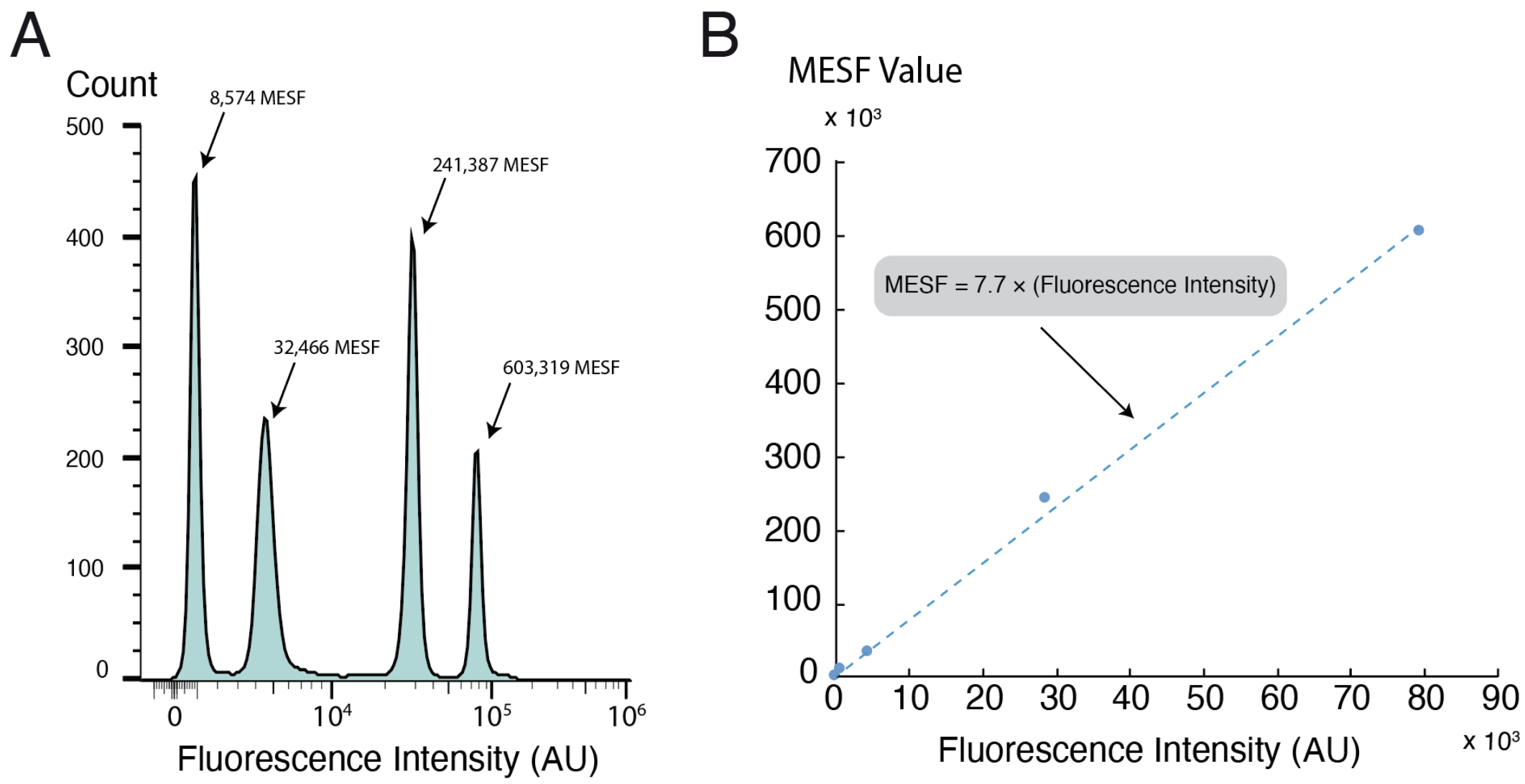

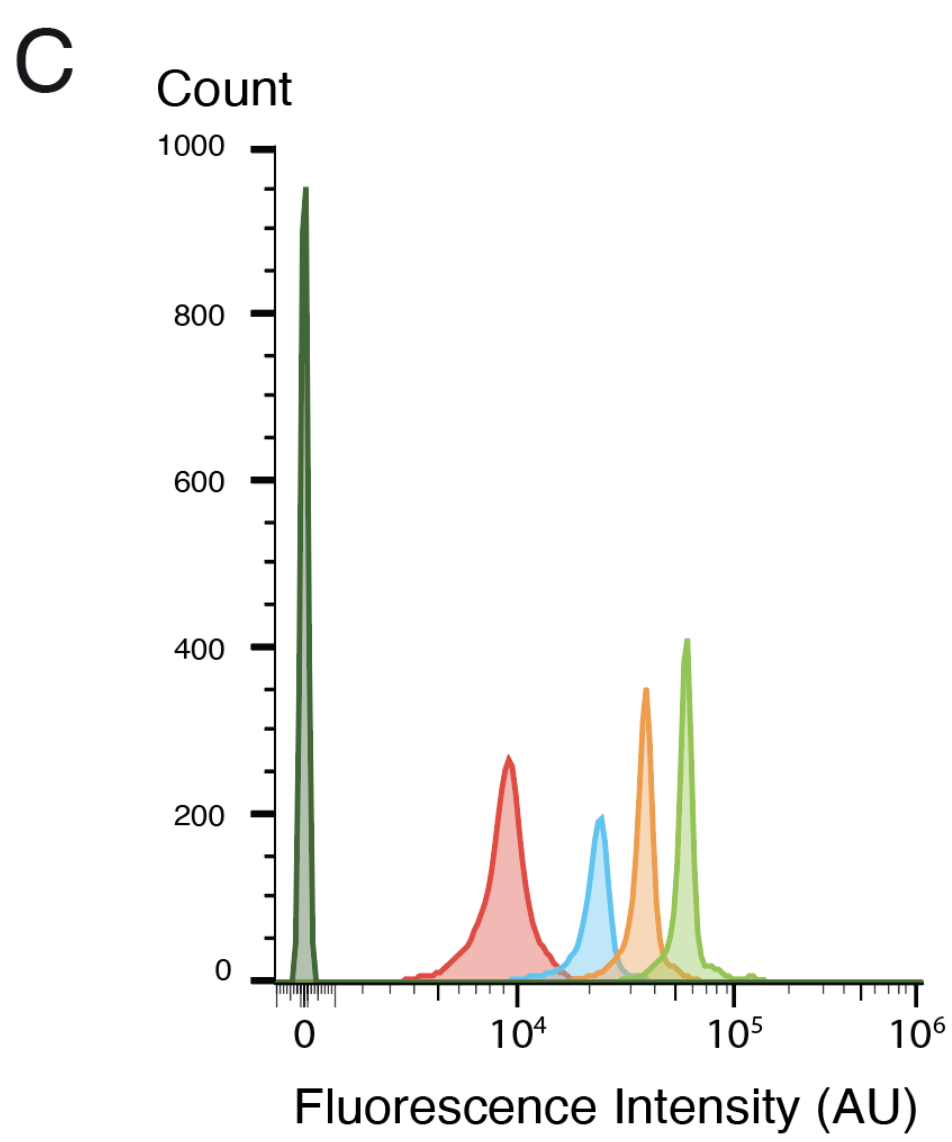

Condition	MESF	Fibcon Density [µm-2]
No Fibcon	0	0
0.5% Ni-NTA	70,207	1,396
1.6% Ni-NTA	159,069	3,164
2.7% Ni-NTA	265,814	5,288
5% Ni-NTA	400,432	7,966

Supplementary Fig. 1. Quantification of surface protein densities. A. Histogram of measured fluorescence intensities for calibrated Alexa Fluor 647 MESF beads. **B.** Calibration curve of MESF values vs. fluorescence intensity for calibrated MESF beads. The fit of the data (simple linear regression R^2= 0.9992) yields an equation for converting fluorescence intensity to MESF. **C.** Histogram of target particles displaying Fib3L that was fluorescently labeled with Alexa Fluor 647 using maleimide chemistry to react with a lone cysteine on the protein (yielding 1 fluorophore per Fib3L protein). Ni-NTA densities were varied to match those used in other experiments and the measured fluorescence intensity was converted to MESF and ultimately to an absolute Fibcon density by dividing by the surface area of the target (4 µm diameter).

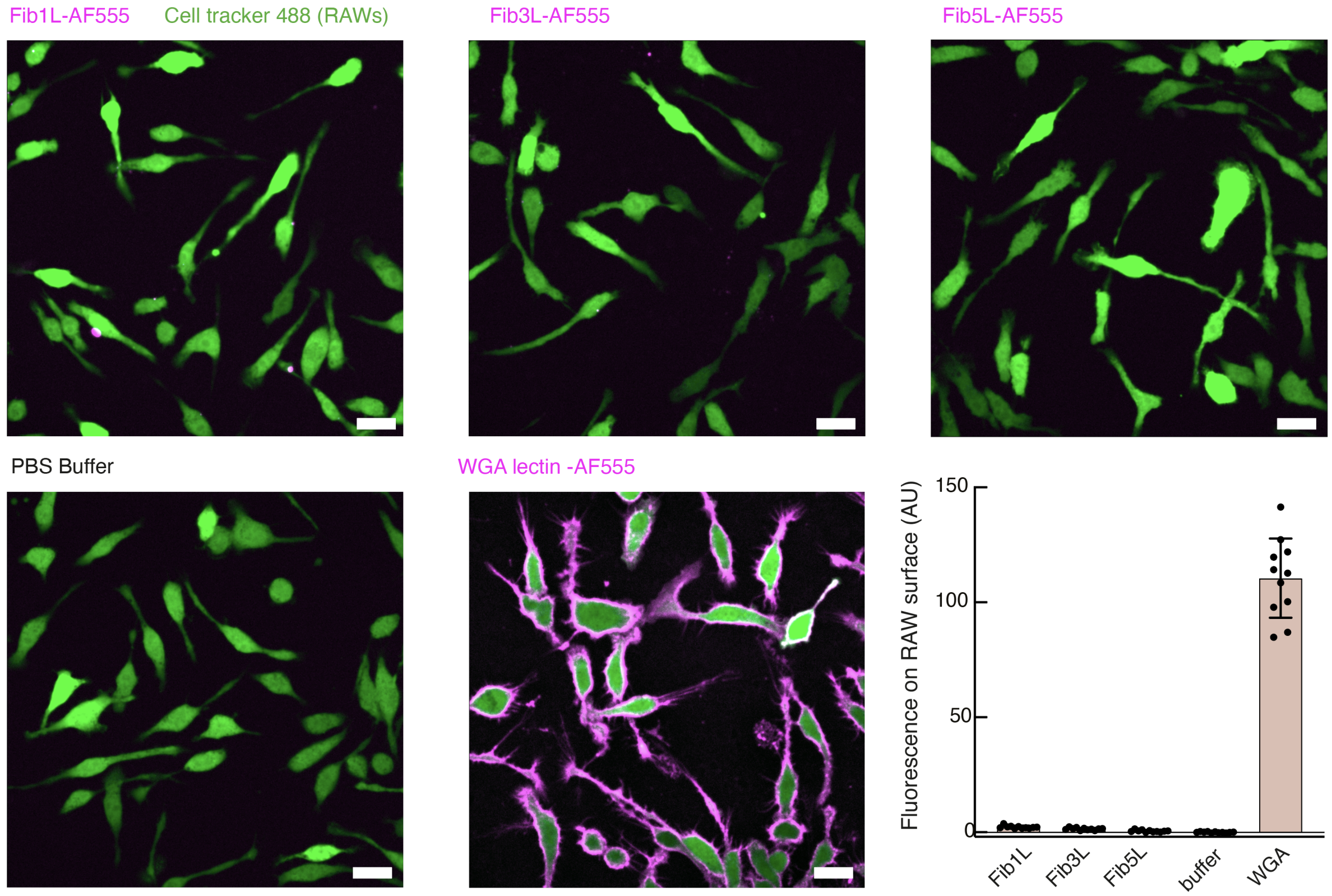

Supplementary Fig. 2. Minimal nonspecific binding of Fibcon constructs to RAW macrophages. Representative epifluorescence images of CellTracker 488–labeled RAW264.7 macrophages (green) incubated with AF555-labeled Fibcon constructs (magenta; Fib1L, Fib3L, Fib5L) or PBS buffer control. As a positive control for cell-surface labeling, cells were stained with AF555–wheat germ agglutinin (WGA). Right, quantification of AF555 fluorescence measured on the RAW surface (AU) shows negligible signal for Fibcon constructs and buffer compared to WGA (points: n=11 individual cells per conditions; bar: mean ± SD, A t-test yielded p-values <0.0001 (Fib1L vs. WGA), <0.0001 (Fib3L vs. WGA), <0.0001 (Fib5L vs. WGA), and <0.0001 (buffer vs. WGA); n=11 cells for each conditions. Scale bar is 20µm).

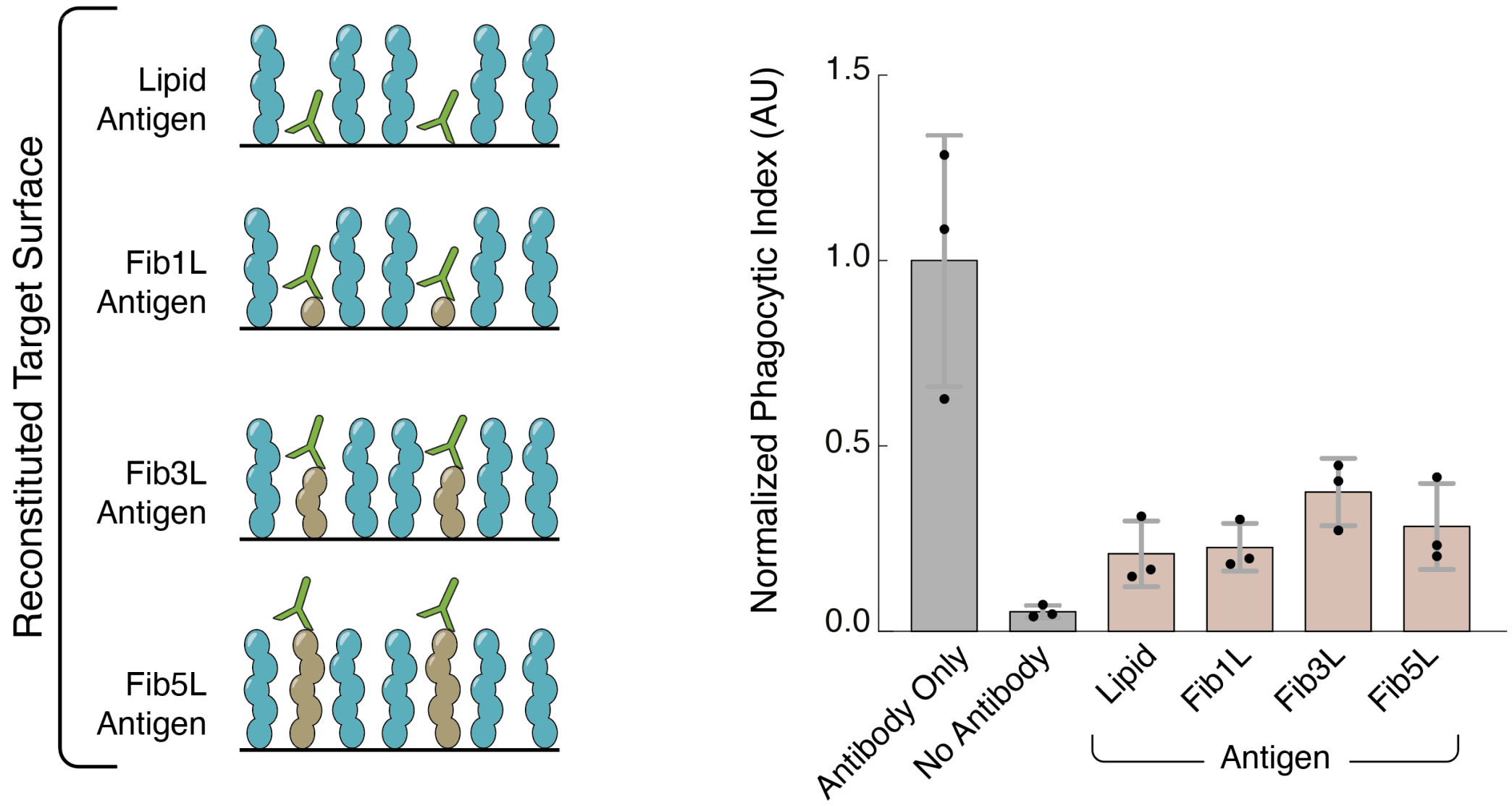

Supplementary Fig. 3. Reducing the height difference between bystander proteins and antibody-bound antigens increases phagocytosis. Phagocytosis was assayed for reconstituted targets displaying different antigen heights in the presence of Fib5L bystander proteins. Antigen densities were fixed at 200/μm^2 and bystander proteins were present at the highest possible density (8,000/μm^2). When compared to the positive control condition (Antibody Only), phagocytosis is significatly inhibited for all antigen sizes (t-test, p= 0.017 (Lipid), p=0.018 (Fib1L), p=0.037 (Fib3L), p=0.026 (Fib5L), maximum phagocytosis occurs for Fib3L antigen (≈10nm) (t-test, p= 0.08 (Lipid vs Fib3L). bar: mean ± SD for N=3 biological replicates of the same condition.

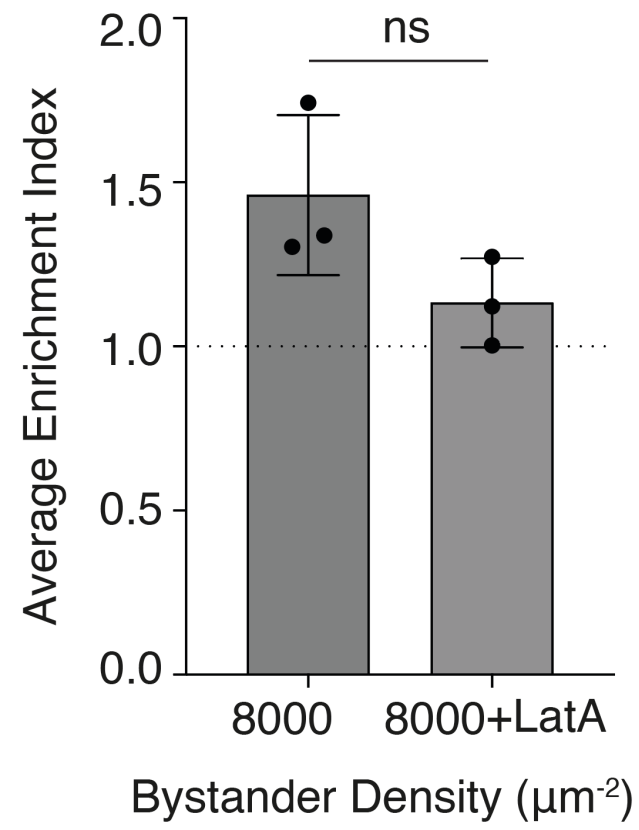

Supplementary Fig. 4. LatA does not significantly change the IgG enrichment index at 8,000/µm^2 Fib5L density. (A) At a Fib5L bystander density of 8,000/µm^2, the IgG enrichment index does not differ significantly between macrophages treated with LatA (an actin polymerization inhibitor) and untreated controls (t-test, $p = 0.11$). bar: mean ± SD. Each condition includes values from n>35 macrophages per day across N = 3 biological replicates.

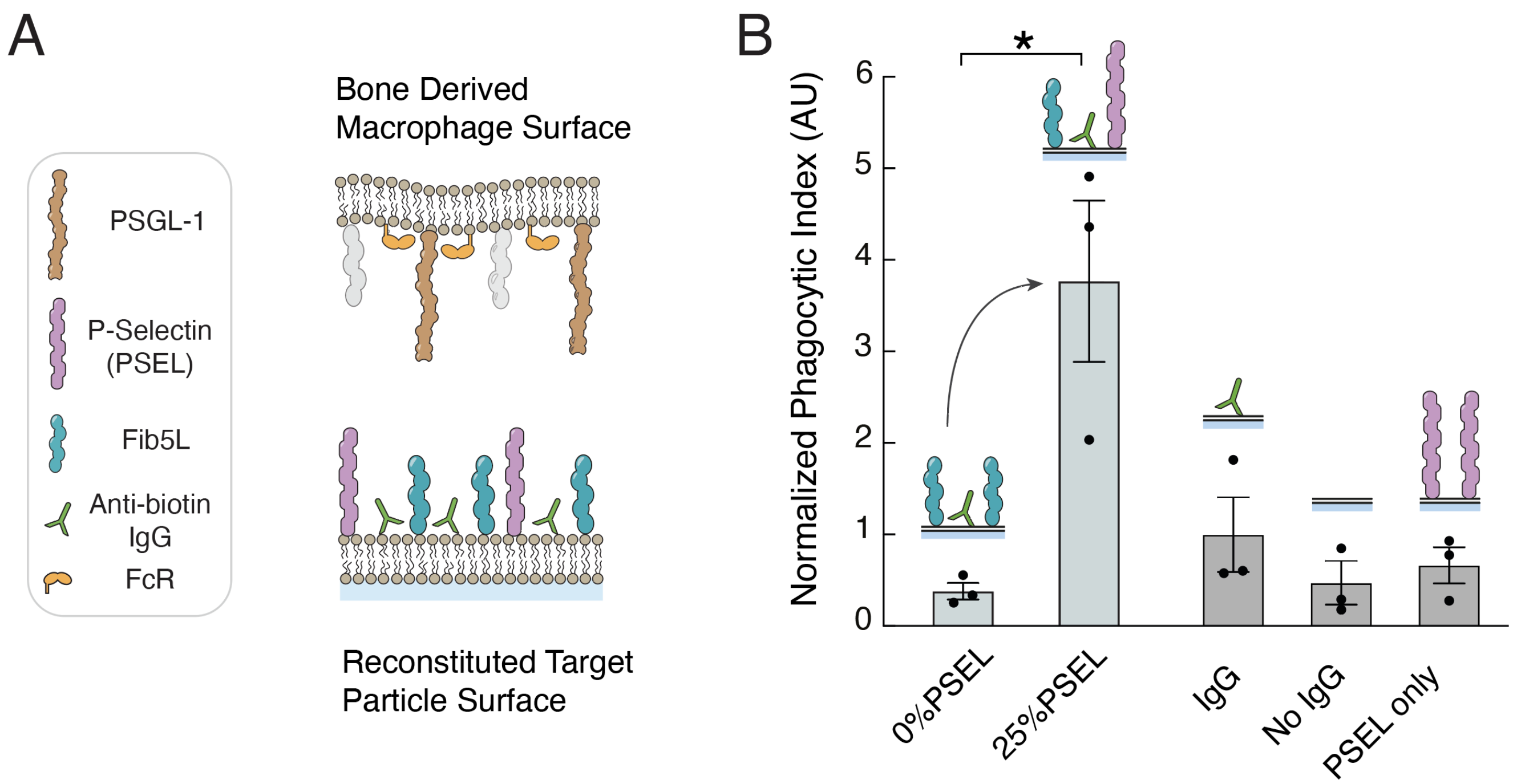

Supplementary Fig. 5. P-selectin rescues Fib5L-mediated inhibition of phagocytosis in bone-derived macrophages. (A) Schematic of the reconstituted phagocytosis assay in which bone-derived macrophages engage glass-bead targets coated with an SLB presenting anti-biotin IgG, Fib5L bystanders, and P-selectin (PSEL). **(B)** Normalized phagocytic index: Fib5L inhibits uptake on IgG-opsonized targets (0% PSEL), reducing phagocytosis to the No-IgG condition (t-test, $p = 0.74$). Adding PSEL restores phagocytosis (25% PSEL vs. 0% PSEL, t-test, $p = 0.018$). PSEL-only targets are not significantly different from No-IgG (t-test, $p = 0.57$). Points: biological replicates; bars: mean ± SD.

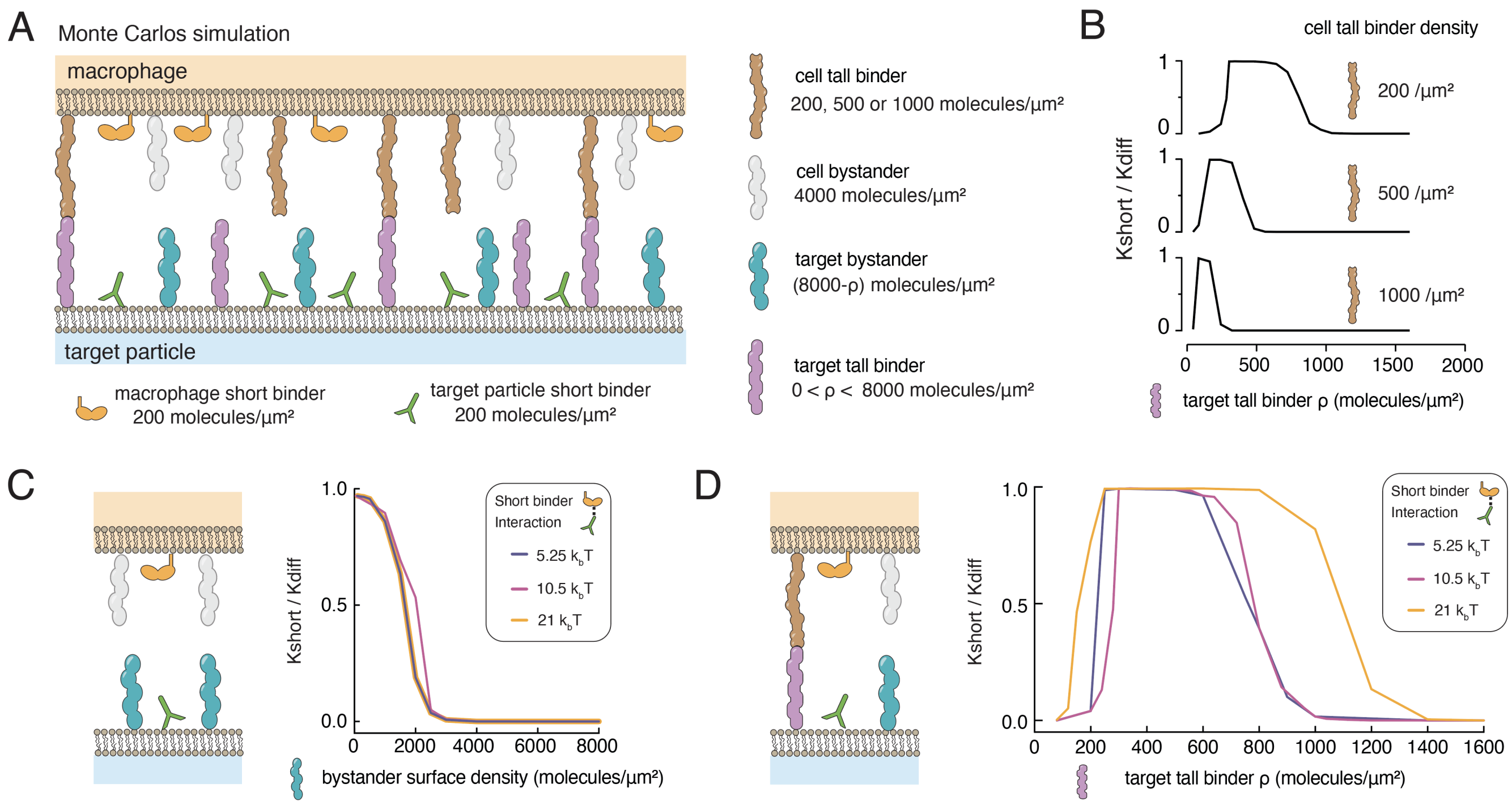

Supplementary Fig. 6. (A) Monte Carlo simulations of a model macrophage-target interface with bystander molecules and short binders mimicking the FcR-antibody pair. Tall binder molecules, consisting of cell and target components, mimic the PSEL-PSGL1 pair. The densities of short binders (both cell and target) were fixed at 200 molecules/µm^2, while cell-side bystanders were maintained at 4000 molecules/µm^2. The tall binder density was varied by independently adjusting two parameters: the cell tall binder density (200, 500, or 1000 molecules/µm^2) and the density (ρ) of target complementary tall binders (0 to 4000 molecules/µm^2). To maintain a constant total molecular density of 8000 molecules/µm^2 on the target surface, the target bystander density was adjusted accordingly. **(B)** Corresponding kinetic rate of short binder engagement as a function of target tall binder density (ρ) demonstrates density-dependent modulation of kinetic enhancement regimes across different cell tall binder densities. For the kinetic enhancement effect to start, a sufficient number of tall binder pairs must form so that the interaction energy (F_{int}) is large enough to lower the activation barrier (see SI text 1B; Steady state kinetics). When cell tall binders are present at low density, a high density of target tall binders is needed to reach sufficient probability of forming enough tall binder pairs, which explains why the enhancement effect ($k_{\text{short}}/k_{\text{diff}} \approx 1$) begins at high target tall binder densities (top curve, 200/µm^2). Conversely, for high cell tall binder densities (lower curve, 1000/µm^2), the critical number of pairs is reached at a lower density of target tall binders. A kinetic enhancement plateau ($k_{\text{short}}/k_{\text{diff}} \approx 1$) persists while the pair's density remains sufficient. However, above a critical value, too many tall binder pairs stabilize the intermediate state, maintaining the membranes at a distance (binder pair length) where membrane fluctuations become insufficient for short binder engagement. This causes the engagement kinetics to collapse ($k_{\text{short}}/k_{\text{diff}} \approx 0$). For low cell tall binder density (upper curve, 200/µm^2), a high concentration of target tall binders is required to form enough pairs to stabilize the interface at an excessive distance—explaining the wide plateau of kinetic enhancement. In contrast, at high cell tall binder density (lower curve, 1000/µm^2), this critical number of pairs is achieved at much lower target tall binder densities, resulting in a narrower kinetic enhancement zone. **(C)** Kinetic rate of short binder engagement as a function of target bystander density, shown for different values of short-binder interaction energy, demonstrates that the interaction energy does not affect the kinetics. **(D)** Kinetic rate of short binder engagement as a function of target tall binder density (ρ) for different short-binder interaction energies. Increasing the interaction energy has a modest effect, slightly broadening the kinetic-enhancement regime, but it does not fundamentally change the overall shape of the curves, suggesting a similar underlying mechanism.

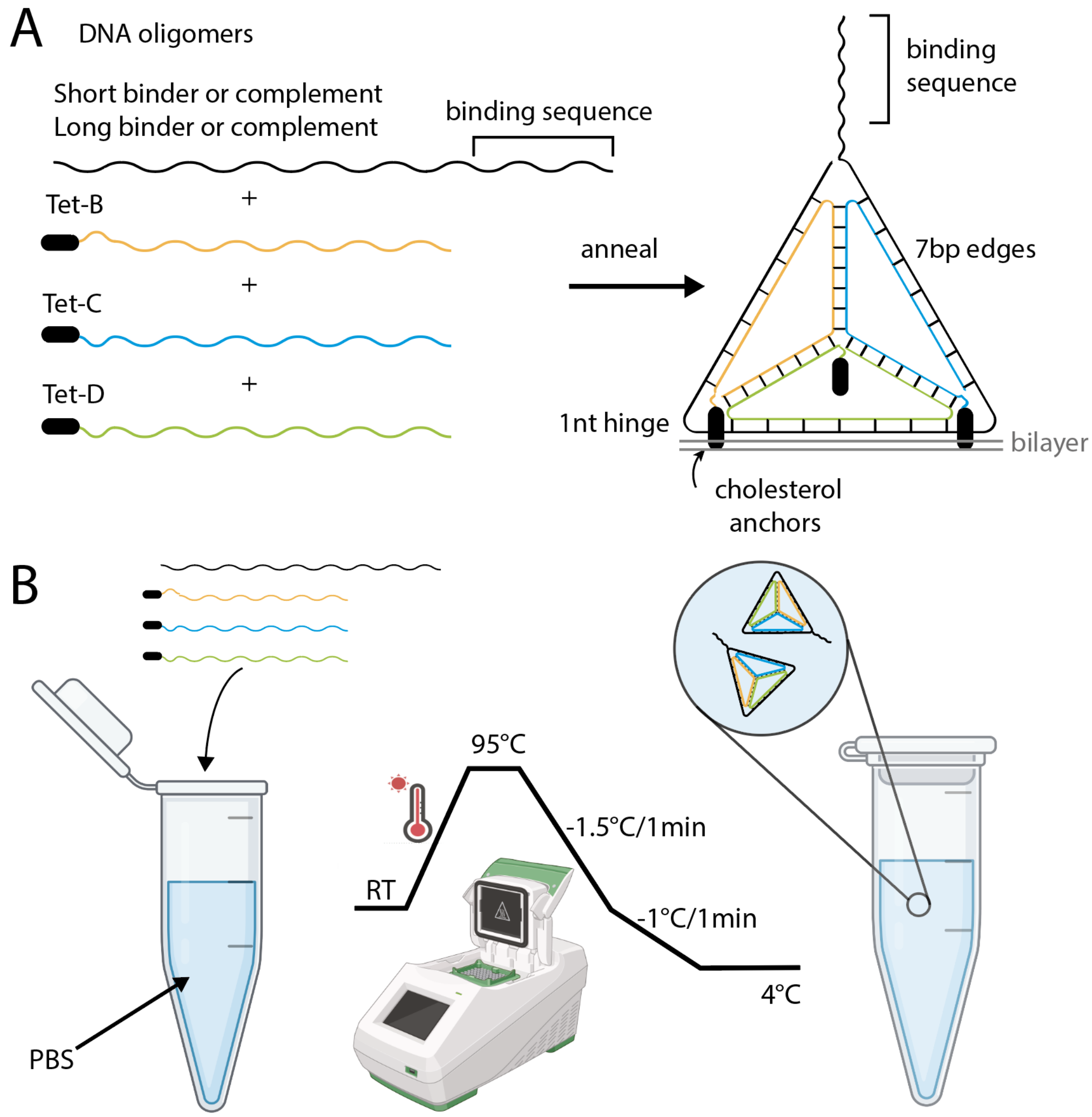

Supplementary Fig. 7. Formation of DNA Origami-Based Synthetic Binders Used in In Vitro Reconstitution (A) Tetrahedra were synthesized by mixing and annealing four DNA oligomers. Upon cooling, the strands formed a tetrahedral shape with 7-bp edges and one long single-stranded domain protruding from the vertex. **(B)** The individual DNA strands were mixed at equimolar ratios in PBS. They were heated to 95°C to remove any undesired secondary structure. Gradual cooling was then performed to favor the formation of the intended, most thermodynamically stable state—the tetrahedron. Created in BioRender. Huzar, J. (2026) https://BioRender.com/cxl2x8d.

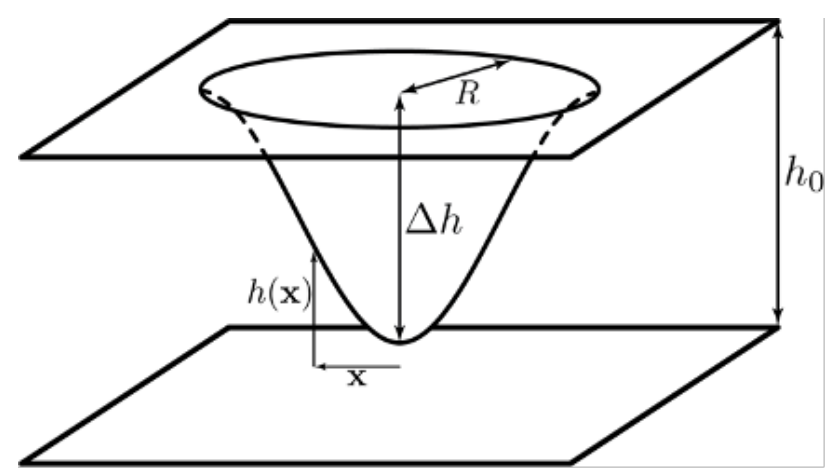

Supplementary Fig. 8. Dimple Thermodynamics Parameterization. Refers to the section Dimple Thermodynamics of the Theoretical modeling.

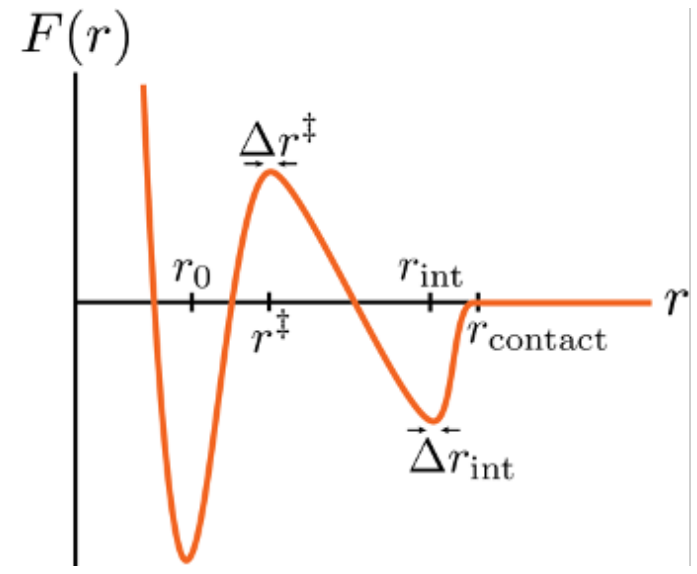

Supplementary Fig. 9. Free energy profile of membrane contacts. Refers to the section Steady state kinetics of the Theoretical modeling.

Supplementary Tables

	Parameter	Value
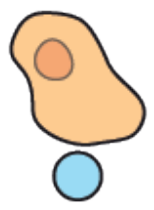	Cell membrane bending modulus	20 $k_B T$
	Target (Bead) supported bilayer	rigid
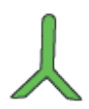	Target Short binder (mimic IgG)	
	Length	7.5 nm
	Density (molecules per µm²)	200 /μm^2
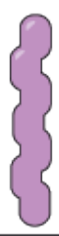	Target tall binder (mimic P-Selectin)	
	Length	30.0 nm
	*Density	0 - 1600 /μm^2
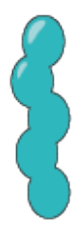	Target Bystander (mimic FIB5L)	
	Length	17.5 nm
	*Density	8000 /μm^2 − PSEL density
	*Density Bystander Only Case	50 - 10000 /μm^2
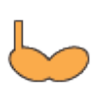	Cell short binder (mimic FcR)	
	Length	3.5 nm
	Density	200 /μm^2
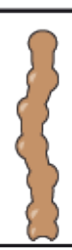	Cell tall binder (mimic PSGL)	
	*Length	15 - 50 nm
	Density	200 /μm^2
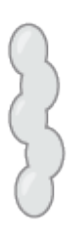	Cell Bystander	
	Length	17.5 nm
	Density	50 - 10000 /μm^2
	Cell + Target contact area	0.7 μm^2
	Molecular diameter	4 nm
	Note: Quantities labeled with * are the sampling parameters	

Supplementary Table 1. Monte Carlo Simulation Parameters. Parameters used to perform Monte Carlo simulations of cell-target contacts.

Names	Sequences	Description
Tet-B	CCGCGGTTGCAGCGCTCACGGCGTT/3CholTEG/	Invariable strand to assemble tetrahedron
Tet-C	ACCGCGGTGGTGGGCTCGGAGCCTT/3CholTEG/	Invariable strand to assemble tetrahedron
Tet-D	GGTCCGCTGCGCTGCTGGCTCCGTT/3CholTEG/	Invariable strand to assemble tetrahedron
Short Binder	/5RhoR-XN/TTTTTCGGCCCGTGCTGTTGCGGACCTGCCCACCTCGCCGTG	Variable sequence to form binding pairs
Short Binder Complement	CAGCACGGGCCGTTGCGGACCTGCCCACCTCGCCGTG	Variable sequence to form binding pairs
Long Binder	/5Alex488N/TTTTTATCATATGACTTTTTTTTTTTTGCGGACCTGCCCACCTCGCCGTG	Variable sequence to form binding pairs
Long Binder Complement	AAGTCATATGATTTTTTTTTTTGCGGACCTGCCCACCTCGCCGTG	Variable sequence to form binding pairs

Supplementary Table 2. DNA oligo sequences were used to assemble DNA origami–based synthetic binding molecules. The oligos were synthesized by IDT.

Hardware	Source	Identifier
5424 Microcentrifuge	Eppendorf	cat # 05-400-005
Lab rotisserie	Thermo scientific	Model M90615 American laboratory trading
Glass coverslip	Avantor	REF 48393-230 24x40mm N 1.5
Camera spinning disk	Teledyne Photometrics	Prime 95B sCMOS camera
Camera TIRF Wide-Field	Hamamatsu	Orca-fusion BT C15440
Microscope	Nikon	ECLIPSE Ti2 inverted microscope
Objectives	Nikon	VSR Plan Apo 20X/0.75 , SR plan Apo IR 60X/ 1.27 WI, Apo Tirf 60x 1.49NA Oil
Spinning disk	Yokogawa	CSU-X1 Spinning Disk Field Scanning Confocal System
Flow cytometer	Thermo Fisher Scientific	Attune NxT equipped with an autosampler and analyzed in FlowJo_v10.8.1

Supplementary Table 3. Hardware used in this study.

Supplementary Note

1. Theoretical modeling

Dimple Thermodynamics

The transition state we propose for bystander-hindered receptor engagement involves a localized deformation of the macrophage membrane that we refer to as a "dimple". Here we estimate the reversible work F_{deform} required to create such a dimple, beginning from an undeformed membrane at height h_0 that allows tall proteins to populate the entire interfacial contact zone. The transition state, sketched in Supplementary Fig. 8, remains undeformed except in a lateral region of radius R. The tip of this circularly symmetric dimple extends a distance Δh below h_0. We adopt a lateral coordinate system x whose origin coincides with the dimple tip, so that the height profile $h(x)$ depends only on the scalar $x = |x|$. In these terms, the geometry described above is specified by the conditions $h(x) = h_0$ and $h(0) = h_0 - \Delta h$.

The membrane's elastic energy E_{elastic}, in the small-gradient limit of Helfrich's model, can be written

$$E_{\text{elastic}} = \frac{1}{2}\kappa_{\text{bend}} \int dx (\nabla^2 h)^2 \qquad [1]$$

A finite elastic energy thus requires additional boundary conditions $h'(R) = h'(0) = 0$. Subject to these constraints, we utilize a smooth, one-parameter interpolation for the dimple profile where the functional $E_{\text{elastic}}[h(x)]$ is minimized by a height profile

$$h(x) = h_0 - \Delta h \left[1 - \left(1+\sqrt{2}\right)\left(\frac{x}{R}\right)^2 + \sqrt{2}\left(\frac{x}{R}\right)^{2+\sqrt{2}}\right] \qquad [2]$$

for which

$$E_{\text{elastic}} = 4\pi\left(1+\sqrt{2}\right)\kappa_{\text{bend}}\left(\frac{\Delta h}{R}\right)^2 \qquad [3]$$

The region of the membrane where $h(x) < h_0$ incurs an additional thermodynamic cost $\pi R^2 w_{\text{evac}}$, where w_{evac} is the reversible work per unit area required to evacuate proteins (and/or protein complexes) whose heights exceed the local membrane gap. In general, protrusion of the dimple would exclude progressively more protein species as $h(x)$ decreases; here, for simplicity, we consider only proteins/complexes of a single height h_0. For a target substrate displaying only IgG and Fibcons, h_0 represents the height of bystander proteins, and $w_{\text{evac}} = \rho_{\text{bystander}} k_B T$ originates in their translational entropy. For a tall adhesive protein like P-selectin, w_{evac} may instead be dominated by the disruption of favorable protein complexation across the membrane gap.

The total free energy of deformation, $E_{\text{elastic}} + \pi R^2 w_{\text{evac}}$, is minimum for a dimple width $R = \left(\frac{4(1+\sqrt{2})\kappa_{\text{bend}}\Delta h^2}{w_{\text{evac}}}\right)^{1/4}$, giving

$$F_{\text{deform}} = 4\pi\Delta h\sqrt{(1+\sqrt{2})\kappa_{\text{bend}} w_{\text{evac}}} \qquad [4]$$

Steady state kinetics

To predict the rate of membrane receptor engagement from computed free energy profiles $F(r)$, we consider overdamped Langevin dynamics of the separation vector r between a macrophage and a target particle, with $F(r)$ as an effective potential energy. In doing so, we assume that all other degrees of freedom relax rapidly by comparison.

For a tagged macrophage/target pair, the probability distribution $p(r,t)$ satisfies a Smoluchowski equation

$$\frac{\partial p}{\partial t} = -\nabla \cdot J \tag{5}$$

with flux $J = -De^{-\beta F}\nabla(e^{\beta F}p)$, where $\beta = (k_B T)^{-1}$ and D is the diffusion coefficient for their relative motion. The rate of phagocytosis (here short binder engagement) is determined from a steady-state solution to Eq. 5, with boundary conditions describing (i) irreversible receptor engagement when the distance $r = |r|$ reaches a minimum value r_0 (i.e., $p(r_0) = 0$), and (ii) asymptotic approach to a constant probability p at large r (i.e., $p(r) \to p$ as $r \to \infty$).

We focus on the rate of short binder engagement k_{short} relative to the diffusion-limited value k_{diff} that would be obtained in the absence of a free energy barrier,

$$\kappa = \frac{k_{\text{short}}}{k_{\text{diff}}} = -\hat{r} \cdot J(r_0)\frac{r_0 V}{D} \tag{6}$$

where $\hat{r}$ is a radial unit vector and V is the macroscopic sample volume. A standard Kramers analysis of this boundary value problem yields

$$\kappa = Vp(r)e^{\beta F(r)}\left(r_0 \int_{r_0}^{r} dr\, \frac{e^{\beta F(r)}}{r^2}\right)^{-1} \tag{7}$$

for all r, and in particular

$$\kappa = \frac{Vp}{r_0}\left(1 + r_0 \int_{r_0}^{r_{\text{contact}}} dr\, \frac{e^{\beta F(r)}}{r^2}\right)^{-1} \tag{8}$$

where r_{contact} is a contact distance beyond which $F(r) = 0$. (See Supplementary Fig.9) Note that r_0 and r_{contact} are both comparable to the macrophage's overall size (∼20 microns in diameter), while $r_{\text{contact}} - r_0$ is a membrane gap scale of roughly 10 nm.

Kinetic suppression from a deeply metastable intermediate manifests in this description as a depletion of p, with substantial population sequestered in the distance interval $r_{\text{int}} \pm\Delta\, r_{\text{int}}$. Here we account for the depletion roughly, by imagining macrophages to be stationary, establishing N_{macro} localized traps where a target particle tends to dwell on the macrophages surface at r_0. Normalization of the steady-state distribution $p(r)$ then gives

$$1 = N_{\text{macro}}\, r_0 \int_{r_0}^{r_{\text{contact}}} 4\pi r^2 p(r)\, dr + Vp \tag{9}$$

Combining Eqs. 7-9, we finally obtain

$$\kappa = (1 + b + c\rho_{macro} r_{contact}^3)^{-1} \tag{10}$$

where $\rho_{\text{macro}} = N_{\text{macro}}/V$.

$$b = r_0 \int_{r_0}^{r_{\text{contact}}} dr\, \frac{e^{\beta F(r)}}{r^2} \tag{11}$$

and

$$c = r_{\text{contact}}^{-2} \int_{r_0}^{r_{\text{contact}}} dr\, 4\pi r^2 e^{-\beta F(r)} \int_{r_0}^{r} dr\, \frac{e^{\beta F(r)}}{r^2} \tag{12}$$

Saddle point approximations for these integrals yield

$$k_{\text{short}} = \frac{k_{\text{diff}}}{\left(1 + \frac{\Delta r^{\ddagger}}{r_{\text{contact}}} e^{F^{\ddagger}/k_BT} + \alpha e^{(F^{\ddagger} - F_{\text{int}})/k_BT}\right)} \quad [13]$$

Here k_{diff} is the rate of diffusion-limited short binder engagement, in which receptor engagement is not impeded by a free energy barrier, Δr_{int} and $\Delta r^{\ddagger}$ are nm-scale membrane gap ranges corresponding to intermediate and transition states, and $r_{\text{contact}} \approx 1$μm is the distance between the center of a macrophage and the center of a target particle when they contact. The number density of macrophages $\rho_{\text{ macro}}$ is on the order of 10^{11} in our experiments, so $\alpha = 4\pi\rho_{\text{macro}} r_{\text{ contact}} \Delta r^{\ddagger} \Delta r_{\text{int}}$ is set to 1x10^{-11} to resemble experimental conditions. Eq. 13 makes clear that an intermediate state which does not lower $F^{\ddagger}$ cannot offer kinetic enhancement. For the free energy profiles we have computed, one term in the denominator of the right-hand side typically dominates over the others, establishing three distinct dynamical regimes: (i) Without a high barrier or a significantly stable intermediate ($F^{\ddagger}$, $\text{F}_{\text{int}} \approx 0$), kinetics is diffusion-limited. (ii) Absent a deeply metastable intermediate, a high barrier ($F^{\ddagger} \gg k_BT$) gives the simple transition state theory estimate $k_{\text{short}} \approx k_{\text{diff}}\ e^{-F^{\ddagger}/k_BT}$. In this regime tall adhesive proteins can substantially offset kinetic effects of bystanders by lowering $F^{\ddagger}$. (iii) An interfacial free energy minimum is so deep that $F^{\ddagger} - \text{F}_{\text{int}}$ exceeds even $k_BT|\ln\alpha|$ and will suppress barrier crossing, regardless of the value of $F^{\ddagger}$.

2. Monte Carlo Simulations

To examine and quantify the role of microscopic fluctuations in bystander-hindered phagocytosis, we performed Monte Carlo simulations of a model adapted from the work of Liposwky and coworkers [1,2]. It resolves topography of the macrophage membrane at a discrete lateral scale $\Delta x_{\text{membrane}}$ (the target membrane is taken to be completely rigid), and treats the populations and spatial arrangements of membrane-bound proteins as a (laterally) noninteracting lattice gas. Proteins can occupy sites on a pair of two-dimensional lattices, representing the macrophage (upper) and target (lower) membranes, subject to constraints that (i) no more than one protein can simultaneously occupy a given lattice site, and (ii) the local vertical separation between upper and lower membranes must exceed $h_{\text{upper}} + h_{\text{lower}}$ in order to accommodate proteins of height h_{upper} and h_{lower} in the upper and lower membranes. When an appropriate pair of proteins (FcγR/IgG or P-selectin/PGSL-1) are apposed across the membrane gap and separated vertically by less than a threshold distance l_{bind}, their interaction contributes a favorable energy of binding $-\epsilon^{(bind)}$.

For a fixed membrane shape, protein population fluctuations can be integrated out exactly in a grand canonical ensemble, yielding a free energy

$$F[h(x)] = \frac{\kappa}{2} \sum_x \left(\nabla^2 h\right)^2 + \sum_x U(h(x)) \quad [14]$$

that governs fluctuations in local membrane separation $h(x)$. Here, $\Delta x_{\text{protein}}$ is the lateral footprint of a protein (which we take to be the same for all proteins), ∇^2 is a discrete approximation to the 2-D Laplacian operator, and $U(h)$ is an effective potential due to the constraints described above. In terms of possible protein occupation states i (which may include protein(s) in the upper and/or lower membranes,

$$U(h) = -k_BT \ln\left(\frac{\Delta x_{\text{protein}}}{\Delta x_{\text{membrane}}}\right)^2 \ln\left(\sum_i \theta(h - h_i) z_i e^{\beta\epsilon_i^{(\text{bind})}} \theta(h_i + l_{\text{bind}} - h)\right) \quad [15]$$

where h_i is the summed height of proteins present, z_i is the product of their activities, $\epsilon^{(bind)}$ is their binding energy (which vanishes in all states except Fc γ R/IgG and P-selectin/PSGL-1), and $\theta(y)$ is the Heaviside step function. The activity of a protein in this description, expressed in terms of its surface density ρ is $\rho \Delta x_{\text{protein}}{}^2/(1 - \rho \Delta x_{\text{protein}}{}^2)$.

Our MC simulation consists of attempts to change $h(x)$ at a single, randomly chosen lattice site x. The change in height is also chosen at random from the interval [-dh, dh]. The proposed new height for the chosen lattice site's is accepted with the standard Metropolis probability $[1, e^{-\beta(\Delta F_{\text{membrane}}+\Delta U+\Delta F_{\text{bias}})}$, where $\Delta F_{\text{membrane}}$ is the associated change in the elastic energy of the membrane, ΔU is the change in free energy due to the proteins, and ΔF_{bias} is the change in a biasing potential designed to improve sampling of important but unlikely configurations.

Free energy profiles $F(r)$ were computed along a membrane gap coordinate $r = \min_n h(x)$ that parametrizes progress through the barrier-crossing pathways of Supplementary Fig. T2 and is well-suited to the kinetic description of Eq. 5. To obtain $F(r)$, we compute and integrate the mean force $\langle f(r)\rangle \approx 2k_{\text{bias}}\langle(r - r_0)\rangle$ from the average deviation of r away from the the value favored by a stiff harmonic bias potential $F_{bias} = k_{\text{bias}}(r - r_0)^2$.

A 0.7 µm^2 elastic sheet was discretized into a 70x70 square grid composed of 12x12 nm^2 patches. The values of the physical parameters used in the simulation are given in the Extanded Table 1. An umbrella sampling simulation protocol for r sampling was performed in a range of 15-80 nm. Each simulation was equilibrated for 2x10^5 Monte Carlo sweeps before data was collected from an additional number of MC sweeps between 8x10^5 and 2x10^6. Changes in height were drawn from the interval [-1.2, 1.2] nm, leading to a 60% acceptance rate.

References

[1] R Lipowsky and B Zielinska. "Binding and unbinding of lipid membranes: A Monte Carlo study". en. In: *Phys. Rev. Lett.* 62.13 (Mar. 1989), pp. 1572–1575.

[2] R Lipowsky. "Adhesion of membranes via anchored stickers". en. In: *Phys. Rev. Lett.* 77.8 (Aug. 1996), pp. 1652–1655.

[3] Jan Liphardt et al. "Equilibrium information from nonequilibrium measurements in an experimental test of Jarzynski's equality". en. In: *Science* 296.5574 (June 2002), pp. 1832–1835.

[4] Subhadip Raychaudhuri, Arup K Chakraborty, and Mehran Kardar. "Effective membrane model of the immunological synapse". en. In: *Phys. Rev. Lett.* 91.20 (Nov. 2003), p. 208101.

[5] Arup K Chakraborty and Andrej Kosmrlj. "Statistical mechanical concepts in immunology". en. In: *Annu. Rev. Phys. Chem.* 61.1 (2010), pp. 283–303.
