# Supplementary material for: Tall adhesions overcome cell surface steric interference and rescue antibody-dependent phagocytosis in macrophages
Source: Nat Commun. 2026 Sep 28;17:11213. doi: 10.1038/s41467-026-78095-7 (PMC13720194; doi:10.1038/s41467-026-78095-7)
Supplement: Supplementary file 2 [file 41467_2026_78095_MOESM2_ESM.pdf]

nature portfolio

Peer Review File

Tall adhesions overcome cell surface steric interference and rescue antibody-dependent phagocytosis in macrophages

Corresponding Author: Dr Daniel Fletcher

This file contains all reviewer reports in order by version, followed by all author rebuttals in order by version.

Version 0:

Reviewer comments:

Reviewer #1

(Remarks to the Author)
In this paper, authors investigate how non-binding "bystander" proteins on target cell surfaces sterically inhibit antibody-dependent cellular phagocytosis. They propose a solution: adding tall “binder” proteins (e.g., P-selectin) can partially rescue phagocytosis through what the authors call "kinetic enhancement." The work combines live cell experiments, a reconstituted GUV system and Monte Carlo simulations.
The main finding that tall adhesive molecules can rescue short binder engagement blocked by bystanders, but only within a certain density window is mechanistically interesting. The non-monotonic dependence of phagocytosis on tall binder density is also a novelty. The authors test their central claims using complementary methods such as macrophage phagocytosis assays, live cell imaging, a cell-free GUV reconstitution and computational modeling. The convergence across these systems strengthens the main argument considerably. Moreover, the quantitative experiments have been carried our rigorously.
Overall, the manuscript contributes important knowledge to the field and therefore I recommend the paper for publication. Some suggestions, in my opinion, that would improve the manuscript are below.
1. The mechanistic claim about kinetic vs. thermodynamic effects is underspecified. The term "kinetic enhancement" is central to the paper but is explained somewhat loosely. Is it inspired by “Kinetic segregation” (which is also not an intuitive terminology)? It should clearly stated that what the term implies.
2. Related to this terminology, actin cytoskeleton role should be discussed: The LatA result (Figure 2C) suggests active processes are important for overcoming steric barriers, which somewhat complicates the authors' argument that equilibrium thermodynamics are sufficient to explain kinetic enhancement. This deserves more explicit discussion and maybe more experiments. For this, Figure 3 seems like an excellent assay to test the role of actin cytoskeleton, and it would really strengthen the model shown in Fig 3A: are these membrane intrusions due to active forces, or passive membrane undulations?
3. Controls for P-selectin specificity: The paper demonstrates that P-selectin rescues phagocytosis but does not show whether this requires active PSGL-1 engagement or is simply a consequence of increased adhesion. Authors generalize the concept that any binders would enhance the synapse, but this should be strengthened. A key control could be a different binder and/or binding-deficient P-selectin mutant at equivalent surface densities for the cell assays. I think this matters for the theoretical interpretation.
4. The Monte Carlo model contains important simplifying assumptions that limit its quantitative relevance. The authors acknowledge this briefly but do not fully discuss the implications: Membrane proteins are modeled as rigid rods on a lattice which ignores flexibility. The authors note in the Discussion that binder flexibility is likely important, yet Figure 4E shows that the long DNA binder (which is highly flexible) remains at the interface rather than being excluded, a behavior the model cannot capture?
Minor Concerns
- Figure 3E positive control: The normalized phagocytic index for the IgG-only positive control appears to be ~2.5, which is notably higher than the same control in Figure 1C (~1.8). The source of this discrepancy across experiments should be addressed, as it affects interpretations such as "70% recovery."
- Statistical reporting: Generally, it would be a lot easier to see the data distribution if all data is shown instead of bar graphs. Moreover, t_enrich data in Figure 2E uses box plots, but the text reports medians. For the reported sample sizes a non-parametric test may be more appropriate than the Student's t-test given the visibly skewed distributions shown.
- Inhibitory role of the tall non-binders was shown for T cells, they might be worth checking (PMC10036606; PPR1112689)
- Some characters in the equations are lost.

Reviewer: Erdinc Sezgin

(Remarks on code availability)

Reviewer #2

(Remarks to the Author)
The manuscript by Joffe and colleagues addresses an interesting question. Understanding which factors regulate FcR-dependent phagocytosis of opsonized material is of great importance. As FcgRs are relatively small cell surface proteins one major lack of understanding is how access of IgG antibodies bound to target cells occurs in the presence of many other much larger cell surface proteins present on the same cell (here: the macrophage). The authors use experimental as well as in silico modelling approaches to tackle this question. In the current study the authors generate a model system with impaired phagocytosis of opsonized beads. This is achieved by incorporating Fib5L multimeric complexes on the target beads, which are coated with an artificial membrane and IgG antibodies. The authors demonstrate that they can overcome the impaired phagocytosis of these beads by introducing even larger cell surface proteins (here P-selectin) on the target structure, which seems unexpected. In the second part of the manuscript the authors use in silico modelling approaches to find a rationale to explain the results.
As I am not a biomathematician I will not comment on the biomathematical part of the study. With respect to the experimental approach I would like to mention that the ratio for the chosen experimental model system is unclear to me. Fib5L is a component of the extracellular matrix, a highly complex extracellular structure which is not present on cells or membranes but in tissues. More importantly Fib5L is not just a “large extracellular forest” protein but may actually actively interact with a variety of integrins on macrophages. Thus, not just the size of the protein but actually interactions with macrophage cell surface proteins may actively impair phagocytosis. Further along these lines allowing a different kind of adhesion via the P-selectin/PSGL1 axis may promote interactions favouring close membrane interactions. Thus, not passive biophysical effects but active cellular processes may be underlying the observed rescue of phagocytic activity. To start excluding such interactions several other proteins demonstrating the same effects should be used. Ideally proteins known to be biologically inert (not allowing active binding or triggering of cellular effector functions) in this experimental setup may help to demonstrate biophysical components of the phenotype. In summary the current experimental model system is not ideal to test the underlying hypothesis and as such the conclusions drawn by the authors.

(Remarks on code availability)
An addition reviewer understanding the biopyhsical background should be consulted.

Reviewer #3

(Remarks to the Author)
The manuscript from Fletcher and collaborators uses elegant model systems to define the impact of tall cell-surface bystander proteins on antibody-FcgR engagement and the formation of a phagocytic synapse. They first demonstrate that tall bystander proteins can interfere with antibody-mediated phagocytosis by blocking the formation of a phagocytic synapse. In contrast, if the tall bystander proteins are binding proteins with their cognate ligand, antibody-mediated phagocytosis can be recovered. The authors term this ‘kinetic enhancement’ and use a combination of cell-free model systems to demonstrate how and under what conditions tall binding proteins are able to modify cell membrane transition states to promote short antibody-FcR interaction and form a stable phagocytic synapse. This is highly innovative and exciting work that helps explains how short antibody-FcR interactions can mediate potent phagocytosis when surrounded by dense networks of taller cell surface proteins. The authors also propose strategies that may take advantage of kinetic enhancement to improve the phagocytic activity of antibody-based immunotherapies.

The work uses multiple model systems, with the goal of simplifying the complex environment of the cell surface and phagocytic synapse. This allows for tractable experiments and the development of computer simulations. A limitation of the study is that it does not address how the observed mechanism is impacted by the native diversity of Fc-FcR interactions. Specifically, the antibody used for all experiments is a murine IgG1. Although this is a common subclass, murine IgG2a has higher affinity for murine FcRs, and typically exemplifies more robust phagocytosis, as does IgG3, which has a longer and more flexible hinge region. Similarly, it is unclear which FcR drives phagocytosis on the RAW264.7 cell model used. These cells have been described to express FcgR1, FcgR2b, FcgR3, and FcgR4. Cell surface densities of each differ, as do their affinity for murine IgG1. Understanding if and how variation in antibody-receptor interactions impacts bystander blocking and kinetic enhancement would significantly strengthen the conclusions drawn and give the study a broader impact. The DNA origami system partially addresses this, but only interface formation is achieved with this model, and it is not clear how the strength of the DNA interactions compare to the Fc-FcR interactions. Additional data, modeling, or other evidence to support that the conclusions are generalizable to other Fc-FcR interactions would strengthen the conclusions.

(Remarks on code availability)

Reviewer #4

(Remarks to the Author)
Review of "Overcoming steric inhibition of antibody-dependent phagocytosis with tall adhesions"

Overall this is a nice paper that adds meaningfully to the field of phagocytosis and cell-cell interface formation. The central finding that tall adhesion proteins could rescue antibody-dependent phagocytosis inhibited by bystander proteins is novel and mostly well-motivated, and the combination of live cell experiments, a reconstituted GUV system, theory, and simulations is a strength. The illustrations of experiments are clear and helpful. However, there are critical missing controls regarding the Fibcon proteins and P-selectin signalling, a lack of proper data representation and statistics across multiple figures, and insufficient methods descriptions that must be addressed before the paper is suitable for publication.

Major Comments

1. Fibcon proteins as integrin ligands — critical control missing
Fibcon proteins contain fibronectin-like domains, which are known ligands for αMβ2 and αXβ2 integrins (CR3/CR4), receptors that are expressed at high levels on RAW macrophages and are well-established mediators of phagocytosis. No controls are presented to demonstrate that these receptors are not interacting with the Fibcon bystander proteins and thereby directly influencing the phagocytosis readout. This is a critical issue for interpreting all figures, since integrin-mediated phagocytosis differs substantially from Fc-mediated phagocytosis, including in its kinetics. If CR3/CR4 engagement is occurring, it could provide an alternative explanation for many of the observed effects. The authors should at minimum test phagocytosis of Fibcon-coated particles without IgG under integrin-blocking conditions, and/or use an integrin-blocking antibody in the main phagocytosis assay. This concern also connects to the question of Fibcon orientation raised below.

2. Fibcon protein conformation and orientation
The interpretation of bystander-mediated inhibition depends critically on the assumption that Fibcon proteins are standing upright on the bilayer surface and presenting their full extended height of 3.5, 10.5, or 17.5 nm. How is this confirmed? His-tag coupling to Ni-NTA lipids via a C-terminal tag constrains one end of the protein to the membrane, but does not prevent the protein from lying flat or adopting intermediate orientations, particularly at high surface densities. Random orientation would substantially reduce the effective steric barrier presented by the proteins, and would affect the quantitative interpretation of the density-dependent inhibition data. The authors should provide evidence for upright orientation, or at minimum discuss this as a limitation and consider its implications for the modeling, where rigid upright rods are assumed.

3. Confirmation of antibody presence at the microscopy interface
It should be explicitly confirmed that the anti-biotin IgG antibodies are present and functional at the interface in the TIRF experiments. While the fluorescence quantification implies this, a direct demonstration — for instance showing that antibody fluorescence is detected on the SLB prior to macrophage addition — would strengthen confidence in the experimental system, particularly for readers less familiar with supported lipid bilayer preparations.

4. Kinetic versus thermodynamic framing is inconsistent
The GUV experiments are presented as evidence that equilibrium thermodynamics is sufficient to explain the observed effects, yet throughout the paper the central phenomenon is described as "kinetic enhancement." This tension is never explicitly resolved. If the GUV system equilibrates and still shows bystander exclusion and short binder engagement in the presence of tall binders, this is informative about thermodynamic feasibility, but it does not by itself validate the kinetic mechanism proposed in Figure 5. The authors should clarify what the GUV experiments specifically demonstrate and how they relate to the kinetic framework, or revise the framing accordingly.

5. P-selectin signaling not controlled for
The authors state that P-selectin was chosen in part because it "binds to the macrophage without directly activating or inhibiting phagocytic signaling." This is asserted as a design justification but is not demonstrated. P-selectin binding to PSGL-1 activates downstream signaling cascades in leukocytes, and it is not obvious that RAW 264.7 cells are an exception. The authors should either cite direct evidence that P-selectin/PSGL-1 engagement does not modulate phagocytic signaling in this cell line, perform an appropriate control experiment, or acknowledge this as a meaningful limitation of the interpretation. This is especially important given the close connection to integrin signaling.

6. Latrunculin A experiment is underpowered and over-interpreted
The LatA result is not surprising in isolation, since actin polymerization is a well-established requirement for phagocytosis. More importantly, the effect size is very modest: the enrichment index drops from approximately 1.25 to 1.1 with LatA treatment, compared to the much larger drop from 7.1 to 1.25 caused by bystander addition alone. This experiment is presented only as an inset at a single bystander density, yet the authors draw relatively strong conclusions about the role of active cytoskeletal processes in overcoming the steric barrier. This is also in tension with the thermodynamic interpretation of the GUV data. Either a more systematic treatment across bystander densities is needed, or the conclusions drawn from this experiment should be substantially tempered.

7. All experiments use RAW 264.7 cells
Given the therapeutic implications discussed at length, particularly regarding cancer immunotherapy and the proposed "Big-BiTE" concept, validation of at least the key findings in primary macrophages would be expected. The degree to which RAW 264.7 cells faithfully recapitulate the relevant biology of tumor-associated macrophages is not discussed.

8. The "Big-BiTE" therapeutic proposal is speculative beyond what the data support
The discussion section proposing a "Big-BiTE" therapeutic goes considerably beyond what the current data can support. This should be clearly and explicitly flagged as speculative, and the discussion of its potential clinical utility should be

tempered accordingly.

Moderate Comments
9. Statistics and data presentation are insufficient across multiple figures
This is a pervasive issue that affects Figures 1, 2, 3, 4, and S2:
* Individual data points are not shown on any bar plots. Given the reported sample sizes, this should be straightforward to include, or violin plots should be substituted to convey the underlying distributions.
* Figures 1, 3, and S2 do not state the number of independent experiments or clarify whether error bars represent SD or SEM.
* Figure 2E uses pairwise t-tests for what is effectively a multiple comparison problem. ANOVA followed by an appropriate multiple comparison correction is required.
* Figure 3 reports no statistical tests despite claiming significant differences in the text.
* Figure S2 does not disclose which statistical test was used.
These issues must be corrected consistently across all figures.

10. Methods descriptions are insufficient
Several aspects of the methods need clarification:
* How was the phagocytic index determined from the fluorescence images? The automated CellProfiler pipeline is referenced but not described.
* How were fields of view selected for imaging? Some description of how human bias in FOV selection was minimized is needed.
* Which microscope, objective, and acquisition settings were used for the phagocytosis imaging?

11. Figure 1D: "Fib5L MPE" is undefined at first appearance
The label "Fib5L MPE" appears in Figure 1D without definition in either the figure caption or the surrounding text at that point. The term (membrane-proximal epitope) is only explained later, which creates confusion on first reading. It should be defined at first use.

12. Figure 2C enrichment index heterogeneity
The no-bystander condition in Figure 2C shows an extremely wide distribution of enrichment index values, with the box extending to approximately 35. This heterogeneity is never discussed. Are some macrophages non-responsive? Does cell-to-cell variability in FcγR expression or actin dynamics account for this spread? Some discussion of this point would help the reader interpret the data.

13. Conclusion from Figure S2 / lines 181-186 is not well supported
The conclusion that Fib3L antigen performs better than others in the presence of Fib5L bystanders is stated with more confidence than the data in Figure S2 support. The N is unclear, no SD is shown, and the statistical test used is not disclosed. This should be corrected in line with the general statistics comments above.

Minor Comments
14. Protein density comparison for P-selectin
The comparison between experimentally used P-selectin densities and those on activated platelets is presented with more confidence than the underlying data warrant. The platelet density is back-calculated from two indirect measurements and the uncertainty in this estimate should be acknowledged.

15. Equation display errors in the SI
There are formatting errors in how several equations are displayed, with extraneous boxes appearing in the text. These should be corrected in revision.

16. Modeling assumes rigid upright proteins
Related to comment 2 above, the Monte Carlo model treats all proteins as rigid upright rods. The authors briefly mention in the discussion that protein flexibility could affect the energetics of the intermediate state, but given that this assumption also underlies the experimental interpretation of Fibcon orientation, a more substantive discussion of how deformable or obliquely oriented proteins would change the predictions would be valuable.

(Remarks on code availability)
I didn't see a need to review the code.

Version 1:

Reviewer comments:

Reviewer #1

(Remarks to the Author)
Authors addressed my concerns. I recommend the publication of the manuscript.

Reviewer #2

(Remarks to the Author)
The reviewers have addressed most of my concerns. I would like to point out that the statement that only FcgRIII but not FcgRIIb is a phagocytic receptor needs to be corrected. Especially FcgRIIb on macrophages is known to be a very efficient receptor triggering receptor mediated endocytosis.

Apart from that I have no further concerns.

Reviewer #3

(Remarks to the Author)
The revised manuscript adequately addresses my prior concerns. This is an interesting and exciting study that uses exceptional experimental design to advance our understanding of physical interactions beyond antibody-FcR interactions that are involved in antibody-dependent phagocytosis.

Reviewer #4

(Remarks to the Author)
I commend the authors for a nice revision of this paper. I believe all of my major concerns have been addressed or clarified nicely.

Open Access This Peer Review File is licensed under a Creative Commons Attribution 4.0 International License, which permits use, sharing, adaptation, distribution and reproduction in any medium or format, as long as you give appropriate credit to the original author(s) and the source, provide a link to the Creative Commons license, and indicate if changes were made.
In cases where reviewers are anonymous, credit should be given to 'Anonymous Referee' and the source.
The images or other third party material in this Peer Review File are included in the article's Creative Commons license, unless indicated otherwise in a credit line to the material. If material is not included in the article's Creative Commons license and your intended use is not permitted by statutory regulation or exceeds the permitted use, you will need to obtain permission directly from the copyright holder.
To view a copy of this license, visit https://creativecommons.org/licenses/by/4.0/

Point-by-point "Response to Reviewers"

Response to Reviewer #1

In this paper, authors investigate how non-binding "bystander" proteins on target cell surfaces sterically inhibit antibody-dependent cellular phagocytosis. They propose a solution: adding tall "binder" proteins (e.g., P-selectin) can partially rescue phagocytosis through what the authors call "kinetic enhancement." The work combines live cell experiments, a reconstituted GUV system and Monte Carlo simulations.

The main finding that tall adhesive molecules can rescue short binder engagement blocked by bystanders, but only within a certain density window is mechanistically interesting. The non-monotonic dependence of phagocytosis on tall binder density is also a novelty. The authors test their central claims using complementary methods such as macrophage phagocytosis assays, live cell imaging, a cell-free GUV reconstitution and computational modeling. The convergence across these systems strengthens the main argument considerably. Moreover, the quantitative experiments have been carried our rigorously.

Overall, the manuscript contributes important knowledge to the field and therefore I recommend the paper for publication. Some suggestions, in my opinion, that would improve the manuscript are below.

Comment 1

The mechanistic claim about kinetic vs. thermodynamic effects is underspecified. The term "kinetic enhancement" is central to the paper but is explained somewhat loosely… It should clearly stated what the term implies.

Response:

We agree and thank the Reviewer for pointing this out. By *kinetic enhancement*, we mean increasing the rate of transition state barrier crossing in the presence of long binders compared to the rate without. In the context of cell-cell interfaces, the crowded cell surface proteins create an energetic barrier, and the long binders increase the rate of overcoming the crowding barrier so that FcγR–IgG engagement can occur.

Changes made in the manuscript:

- Improved definition of "kinetic enhancement" (Line 109-111)

Comment 2

Related to this terminology, actin cytoskeleton role should be discussed: The LatA result (Figure 2C) suggests active processes are important for overcoming steric barriers, which somewhat complicates the authors' argument that equilibrium thermodynamics are sufficient to explain kinetic enhancement. This deserves more explicit discussion and maybe more experiments. For this, Figure 3 seems like an excellent assay to test the role of actin cytoskeleton, and it would really strengthen the model shown in Fig 3A: are these membrane intrusions due to active forces, or passive membrane undulations?

Response:

We thank the Reviewer for raising this point. We re-checked the statistical analysis and corrected an error in our analysis. There is, in fact, no statistically significant difference between Fib5L (at 8000/μm2) and Fib5L (at 8000/μm2) + LatA. This result was expected because Fib5L at that surface concentration already imposes an almost complete kinetic barrier; therefore, disrupting actin polymerization with LatA, which could in principle help overcome this barrier, has no additional measurable effect under these conditions. To avoid confusion, we have moved the LatA data to Supplementary Information.

Nevertheless, we agree that active processes, particularly from the actin cytoskeleton, could further increase the rate of overcoming steric barriers and deserve a clearer discussion in the manuscript. We have revised the manuscript to explicitly clarify that (i) our cell-free GUV system demonstrates that kinetic enhancement can be achieved with long binders and without active cellular machinery but that (ii) actin-driven forces could further increase the kinetic enhancement by increasing membrane dynamics and/or pushing through crowded interfaces.

Finally, we attempted the phagocytosis assay shown in Figure 3 in the presence of LatA but observed no phagocytosis, as expected due to the requirement for actin polymerization during cup formation and engulfment [1]. This is not a direct test of whether actin-driven forces contribute to kinetic enhancement but rather a demonstration that inhibition of phagocytosis is due to changes in surface crowding and not dependent on the actin cytoskeleton. To acknowledge the potential role of active processes, we have expanded the discussion of membrane fluctuations (like those displayed in Figure 3A) to include active protrusive forces as well as passive membrane fluctuations. We emphasize however, that active protrusions alone are insufficient to overcome sufficiently large steric barriers, as evidenced by the need for long binders in our macrophage experiments (Figure 3E).

Changes made in the manuscript:

- Re-did the statistical analysis and moved the LatA data to the Supplementary Information.
- Revised the text in the section *"Results/Bystander proteins impede FcγR engagement and dynamics at phagocytic interfaces"* (Line 219-222).
- Noted that active forces could further modulate kinetic enhancement (Line 366-367).
- Expanded the discussion of actin and its possible role in the kinetic enhancement process (Discussion, Line 480-486)

Comment 3

Controls for P-selectin specificity: The paper demonstrates that P-selectin rescues phagocytosis but does not show whether this requires active PSGL-1 engagement or is simply a consequence of increased adhesion. Authors generalize the concept that any binders would enhance the synapse, but this should be strengthened. A key control could be a different binder and/or binding-deficient P-selectin mutant at equivalent surface densities for the cell assays. I think this matters for the theoretical interpretation.

Response:

The Reviewer is correct that P-selectin/PSGL-1 engagement could contribute beyond increased adhesion, for example through signaling, because P-selectin is known to bind immune cells and facilitate their interactions.

We know that P-selectin binds to our model macrophages, as its presence on target beads leads to significant binding compared to bare target beads, almost as much as antibody does (Figure 3D, far right condition). However, the presence of P-selectin alone does not induce phagocytosis (Figure 3E, far right conditions). Since phagocytosis only occurs in the presence of an opsonizing antibody, this suggests that P-selectin/PSGL-1 engagement is not signaling in a way that drives phagocytosis.

Implementing an additional orthogonal tall protein pair, as the Reviewer suggests, is an interesting direction that we are actively pursuing for a future publication on CAR-M cells. In this manuscript, we do include the DNA binders on model GUVs as on orthogonal tall binder that validates the conceptual framework and confirms that the combination of tall and short binders can drive close contact.

Changes made in the manuscript:

- We revised the text to better emphasize the controls we performed to rule out potential PSEL-related signaling. (Line 310-314)

Comment 4

The Monte Carlo model contains important simplifying assumptions that limit its quantitative relevance. The authors acknowledge this briefly but do not fully discuss the implications: Membrane proteins are modeled as rigid rods on a lattice which ignores flexibility. The authors note in the Discussion that binder flexibility is likely important, yet Figure 4E shows that the long DNA binder (which is highly flexible) remains at the interface rather than being excluded, a behavior the model cannot capture?

Response:

We agree that the Monte Carlo model makes simplifying assumptions (rigid rods on a lattice) that limit its ability to quantitatively model cell-cell interfaces, especially in the case of flexible binders. While the model qualitatively captures the role of tall binders, we agree it is important to make the limitations in the model more clear. We now explicitly state that the long DNA binder behavior in Figure 4E is not captured by the current model, since flexibility introduces additional degrees of freedom that can allow interfacial localization even when a rigid-rod model would predict exclusion. Similarly, we have expanded the Discussion to frame the model as capturing the qualitative transition-state barrier mechanism for short binders, while clarifying that flexible long binders would be useful to include in a more detailed model of cell-cell interface formation.

Changes made in the manuscript:

- Expanded model limitations and added explicit discussion of flexibility. Clarified interpretation of DNA binder observations relative to model scope. (Line 495-505)

Minor comments

- **Figure 3E positive control: The normalized phagocytic index for the IgG-only positive control appears to be ~2.5, which is notably higher than the same control in Figure 1C (~1.8). The source of this discrepancy across experiments should be addressed, as it affects interpretations such as "70% recovery."**

  Response:

  Thank you for raising this point. We have updated the figures so the arbitrary units match to avoid confusion.

- **Statistical reporting: Generally, it would be a lot easier to see the data distribution if all data is shown instead of bar graphs. Moreover, t_enrich data in Figure 2E uses box plots, but the text reports medians. For the reported sample sizes a non-parametric test may be more appropriate than the Student's t-test given the visibly skewed distributions shown.**

  Response:

  We agree and have updated the plots to better show distributions across different experimental days and re-evaluated the statistical testing. The text and the caption now reflect these changes.

- **Inhibitory role of the tall non-binders was shown for T cells, they might be worth checking (PMC10036606; PPR1112689)**

Response:

We thank the Reviewer for highlighting this interesting and relevant work. We have now included it as a reference in the text (Line 95).

- **Some characters in the equations are lost.**

  Response: Thank you for noting this. We corrected the equation rendering issues and verified equation display in the journal PDF workflow.

Response to Reviewer #2

The manuscript by Joffe and colleagues addresses an interesting question. Understanding which factors regulate FcR-dependent phagocytosis of opsonized material is of great importance. As FcgRs are relatively small cell surface proteins one major lack of understanding is how access of IgG antibodies bound to target cells occurs in the presence of many other much larger cell surface proteins present on the same cell (here: the macrophage). The authors use experimental as well as in silico modelling approaches to tackle this question. In the current study the authors generate a model system with impaired phagocytosis of opsonized beads. This is achieved by incorporating Fib5L multimeric complexes on the target beads, which are coated with an artificial membrane and IgG antibodies. The authors demonstrate that they can overcome the impaired phagocytosis of these beads by introducing even larger cell surface proteins (here P-selectin) on the target structure, which seems unexpected. In the second part of the manuscript the authors use in silico modelling approaches to find a rationale to explain the results.

As I am not a biomathematician I will not comment on the biomathematical part of the study.

Comment 1

With respect to the experimental approach I would like to mention that the ratio for the chosen experimental model system is unclear to me. Fib5L is a component of the extracellular matrix, a highly complex extracellular structure which is not present on cells or membranes but in tissues.

More importantly Fib5L is not just a “large extracellular forest” protein but may actually actively interact with a variety of integrins on macrophages. Thus, not just the size of the protein but actually interactions with macrophage cell surface proteins may actively impair phagocytosis.

We agree with the Reviewer that fibronectin is a key extracellular matrix protein and that its native form can interact with integrins. However, we do not use native fibronectin domains in this study. Rather, we used a previously published engineered protein composed of repeats of a hyperstable consensus FN-III domain ("Fibcon") [2] that does not contain a binding RGD motif. We used constructs of increasing length (Fib1L, Fib3L, Fib5L), defined by the number of repeated domains. In a previous study [3], we found that Fibcon repeats did not promote phagocytosis of target

particles (see Figure 2D, white bar plots). These constructs were purified from *E. coli* to avoid glycosylation, which is known to increase fibronectin binding affinity [4].

To confirm the lack of interaction, we measured adhesion between macrophages and Fib5L-coated beads. Fib5L alone did not increase adhesion (Figure 3D, compare the PSEL 0% bar to the No IgG bar), arguing against binding to macrophage integrins or other surface proteins. To provide additional evidence and test whether our Fibcon repeats interact with our cells, we incubated RAW cells with soluble fluorescently labeled Fib1L, Fib3L, or Fib5L for 10 min on ice, then washed and imaged the cells. We did not observe any detectable enrichment of the Fibcon repeats on macrophages, in contrast to the positive control WGA lectin, which strongly stained the cell surface (Response Figure 1, new Figure S2). Because of the lack of binding and purely steric role, we refer to the Fibcons as “bystander” proteins.

Furthermore, our main dataset supports a steric interpretation of the Fibcon proteins. Specifically, (i) crowding the target surface with Fibcon repeats decreases phagocytosis (Figure 1C), (ii) phagocytosis decreases as Fibcon size increases (Figure 1D), indicating that molecular height is a key determinant, and (iii) “raising” IgG by anchoring it at the end of progressively longer Fibcon constructs only partially restores phagocytosis (Figure S3).

Taken together, these results support the idea that the main effect of Fibcon in our system is steric in nature, rather than any adhesive interactions.

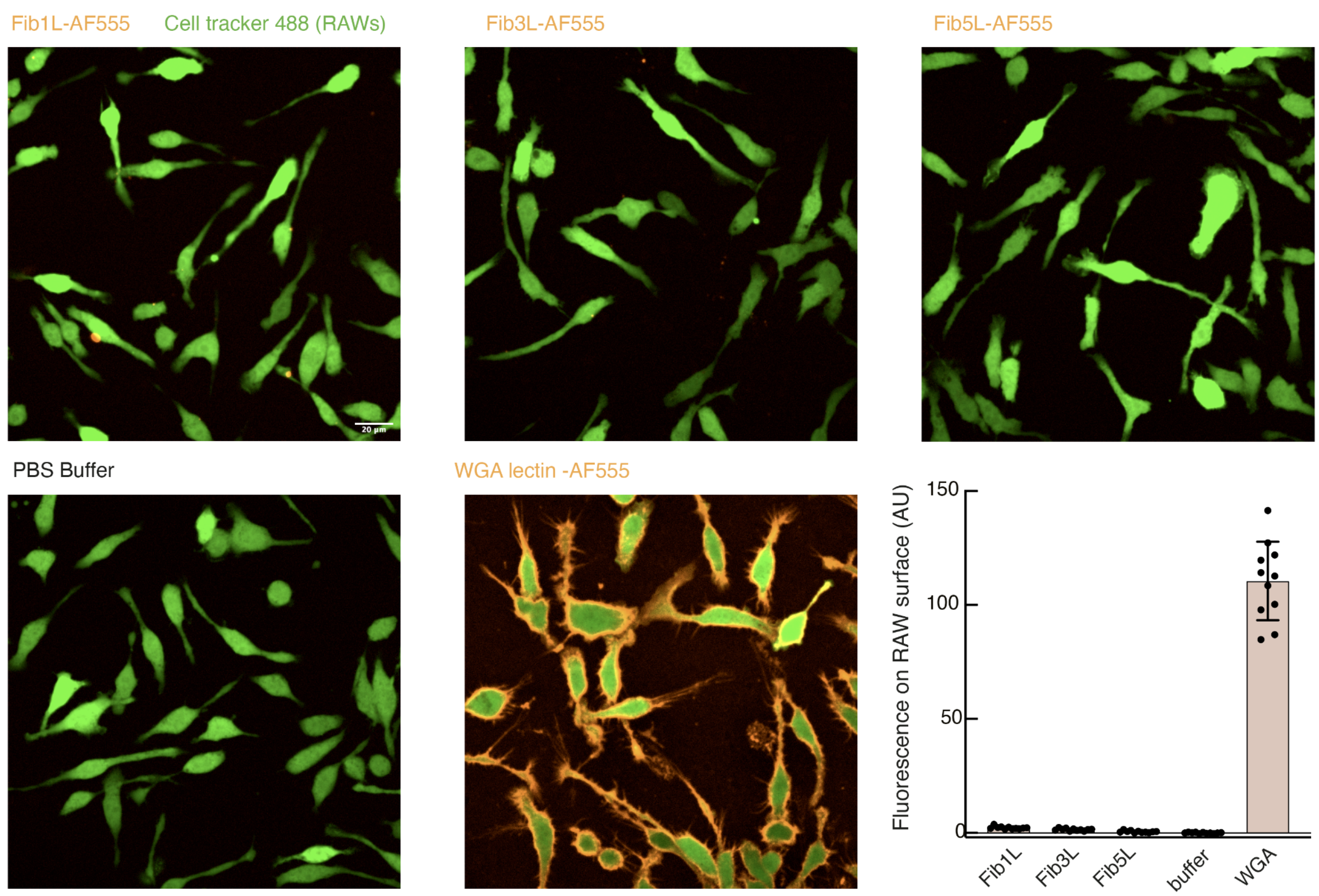

Response Figure 1: Characterization of Fibcon repeat interactions with the surface of macrophages. Representative epifluorescence images of CellTracker 488–labeled RAW264.7 macrophages (green) incubated with AF555-labeled Fibcon constructs (orange; Fib1L, Fib3L, Fib5L) or PBS buffer control. As a positive control for cell-surface labeling, cells were stained with AF555–wheat germ agglutinin (WGA). Right, quantification of AF555 fluorescence measured on the RAW surface (AU) shows negligible signal for Fibcon constructs and buffer compared to WGA (points: n=11 individual cells per conditions; bar: mean ± SD, Scale bar is 20μm).

Changes made in the manuscript:

- We clarified that fibronectin is an extracellular matrix protein, and we better emphasized our strategy of using engineered consensus FN-III repeats (Fibcon) designed to avoid interactions with RAW cell-surface molecules. (Line 150-153)
- We added a paragraph noting that the Fibcon constructs used in this study were previously tested in an identical experimental context and were found not to induce phagocytosis. (Line 153-154)
- We added a solution-binding control showing no detectable enrichment of any Fibcon repeats on RAW's surface (New Figure S2). (Line 155)

Comment 2

Further along these lines allowing a different kind of adhesion via the P-selectin/PSGL1 axis may promote interactions favouring close membrane interactions. Thus, not passive biophysical effects but active cellular processes may be underlying the observed rescue of phagocytic activity.

To start excluding such interactions several other proteins demonstrating the same effects should be used. Ideally proteins known to be biologically inert (not allowing active binding or triggering of cellular effector functions) in this experimental setup may help to demonstrate biophysical components of the phenotype

Response:

We agree that P-selectin/PSGL-1 engagement could trigger signaling, including integrin activation, and contribute to the rescue beyond passive adhesion [5]. This is an excellent point that we considered during the experimental design stage of this project. We therefore tested whether engagement of P-selectin/PSGL-1 alone is sufficient to promote phagocytosis in our assay. Our control experiments show that presenting P-selectin on targets in the absence of IgG increases macrophage–particle association (fraction of macrophages bound to particles; Figure 3D), but does not increase the phagocytic index (Figure 3E). This indicates that P-selectin/PSGL-1 functions primarily as an adhesion module in our assays and is not, by itself, a sufficiently pro-phagocytic stimulus by itself, at least under our experimental conditions.

Instead, the results are most consistent with a biophysical mechanism in which added adhesion increases the probability/extent of close membrane apposition (Figure 3), thereby restoring

productive FcγR–IgG engagement when IgG is present. This interpretation is further supported by our results using a passive, purely adhesive DNA-based system (Figure 4).

Regarding the use of other tall binders in cells, as discussed in our response to Reviewer 1, Comment 3, we are exploring this for a future study in the context of CAR-M cells. In the present paper, our goal is to establish a concept that we hope can be applied in broader immunological contexts.

Changes made in the manuscript:

- Added a section to the manuscript that highlights the control (Figure 3D,E) that rules out a PSEL pro-phagocytic stimulus. (Line 310-314)

Response to Reviewer #3

The manuscript from Fletcher and collaborators uses elegant model systems to define the impact of tall cell-surface bystander proteins on antibody-FcgR engagement and the formation of a phagocytic synapse. They first demonstrate that tall bystander proteins can interfere with antibody-mediated phagocytosis by blocking the formation of a phagocytic synapse. In contrast, if the tall bystander proteins are binding proteins with their cognate ligand, antibody-mediated phagocytosis can be recovered. The authors term this 'kinetic enhancement' and use a combination of cell-free model systems to demonstrate how and under what conditions tall binding proteins are able to modify cell membrane transition states to promote short antibody-FcR interaction and form a stable phagocytic synapse. This is highly innovative and exciting work that helps explains how short antibody-FcR interactions can mediate potent phagocytosis when surrounded by dense networks of taller cell surface proteins. The authors also propose strategies that may take advantage of kinetic enhancement to improve the phagocytic activity of antibody-based immunotherapies.
The work uses multiple model systems, with the goal of simplifying the complex environment of the cell surface and phagocytic synapse. This allows for tractable experiments and the development of computer simulations.

Comments and question

A limitation of the study is that it does not address how the observed mechanism is impacted by the native diversity of Fc-FcR interactions. Specifically, the antibody used for all experiments is a murine IgG1. Although this is a common subclass, murine IgG2a has higher affinity for murine FcRs, and typically exemplifies more robust phagocytosis, as does IgG3, which has a longer and more flexible hinge region. Similarly, it is unclear which FcR drives phagocytosis on the RAW264.7 cell model used. These cells have been described to express FcgR1, FcgR2b, FcgR3, and FcgR4. Cell surface densities of each differ, as do their affinity for murine IgG1. Understanding if and how variation in antibody-receptor interactions impacts bystander blocking and kinetic enhancement would significantly strengthen the conclusions drawn and give the study a broader impact. The DNA origami system partially addresses this, but only interface formation is achieved with this model, and it is not clear how the strength of the DNA interactions compare to the Fc-FcR interactions.

Additional data, modeling, or other evidence to support that the conclusions are generalizable to other Fc-FcR interactions would strengthen the conclusions.

Response:

We thank the Reviewer for raising this point. We agree that the manuscript did not explicitly discuss the diversity of antibody subclasses and FcγR expression in the murine model, nor how this diversity could influence our results. We acknowledge that this is an important point and should be explained in our manuscript.

In designing our experiments, we deliberately chose murine IgG1 to work in a simpler regime that minimizes the range of activating antibody–FcγR interactions. Murine IgG1 predominantly engages FcγRIIB and FcγRIII, and among these two receptors, FcγRIII is the only one that triggers phagocytosis [6]. We therefore expect that the phagocytosis observed in our RAW264.7 model is primarily driven by FcγRIII activation by the IgG1 antibodies used to opsonize our targets.

That said, we agree that determining how differences in the antibody–receptor interaction energy modulates bystander blocking and kinetic enhancement is a very interesting question. As noted by the Reviewer, our DNA-origami data only partially probes the effect of affinity modulation. In this system, the chosen DNA hybridization interactions are on the order of 30 K_bT, i.e., approximately two-fold stronger than values reported for the FcγRIII–IgG1 pair (about 15 K_bT) [7]. Our results therefore show that kinetic enhancement and bystander blocking can operate within this range of interaction energies.

To explore this question further, we leveraged our interface simulation framework to obtain new results addressing this question. Specifically, we simulated the effect of the FcγR–antibody affinity (the "short binder" in the simulation) on both bystander blocking and kinetic enhancement.

Our new simulation data show that increasing the short-binder interaction energy only slightly widens the kinetic-rate curves in the bystander + tall-binder case, while preserving the overall trend (Response Figure 2A and updated Figure S6D of the SI). The effect was negligible in the bystander only case (Response Figure 2B, Figure S6C). The weak dependence on short-binder strength suggests that changing short-binder affinity mainly stabilizes the final close-contact state, with little effect on the barrier-crossing step introduced by the bystander proteins and the tall binders. We interpret these results as evidence that crossing the steric barrier is the rate-limiting step.

Experimentally, future experiments could be carried out that use antibodies with higher-affinity FcγR engagement (for example, IgG2a, which should engage all FcγRs expressed by RAW264.7 cells) and specific FcγR-blocking antibodies to isolate and test each receptor individually. We will save these interesting experiments for a future publication.

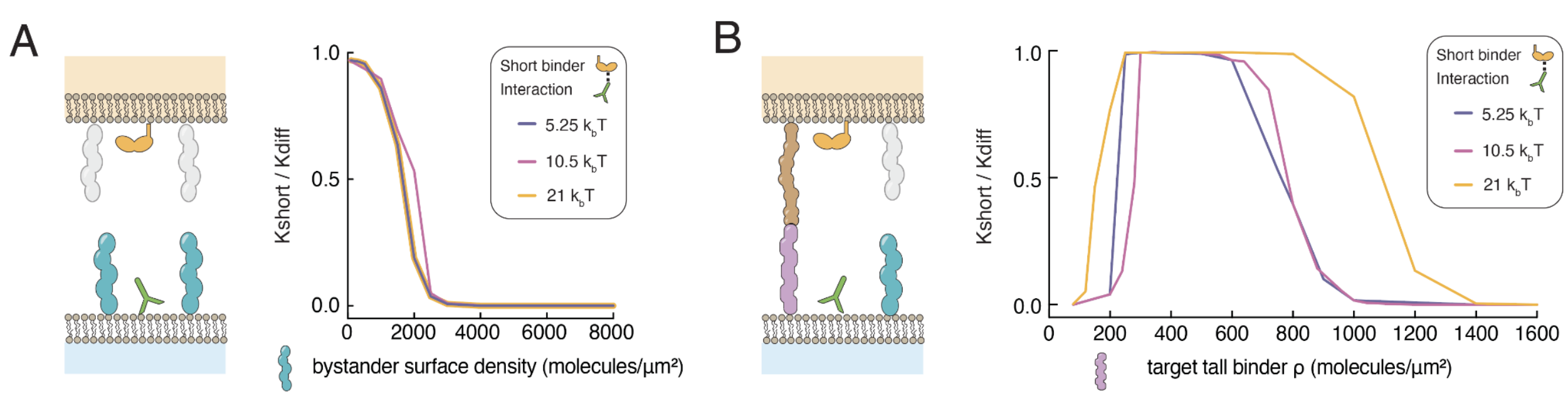

Response Figure 2: Assessing how short-binder affinity modulates kinetic enhancement using MD simulations. (A) Kinetic rate of short binder engagement as a function of target bystander density, shown for different values of short-binder interaction energy, demonstrates that the interaction energy does not affect the kinetics. **(B)** Kinetic rate of short binder engagement as a function of target tall binder density for different short-binder interaction energies. Increasing the interaction energy has a modest effect, slightly

broadening the kinetic-enhancement regime, but it does not fundamentally change the overall shape of the curves, suggesting a similar underlying mechanism.

Changes made in the manuscript:

- Added discussion of FcγR identity and expression in RAW264.7 cells. (Line 146-148)
- Add new simulation data clarifying the role of short binder (FcγR–antibody) affinity in bystander blocking and kinetic enhancement (Manuscript Line 399-401, Line 432-434, Figure S6).

Response to Reviewer #4

Overall this is a nice paper that adds meaningfully to the field of phagocytosis and cell-cell interface formation. The central finding that tall adhesion proteins could rescue antibody-dependent phagocytosis inhibited by bystander proteins is novel and mostly well-motivated, and the combination of live cell experiments, a reconstituted GUV system, theory, and simulations is a strength. The illustrations of experiments are clear and helpful. However, there are critical missing controls regarding the Fibcon proteins and P-selectin signalling, a lack of proper data representation and statistics across multiple figures, and insufficient methods descriptions that must be addressed before the paper is suitable for publication.

Major Comment 1

Fibcon proteins as integrin ligands — critical control missing

Fibcon proteins contain fibronectin-like domains, which are known ligands for αMβ2 and αXβ2 integrins (CR3/CR4), receptors that are expressed at high levels on RAW macrophages and are well-established mediators of phagocytosis. No controls are presented to demonstrate that these receptors are not interacting with the Fibcon bystander proteins and thereby directly influencing the phagocytosis readout.

This is a critical issue for interpreting all figures, since integrin-mediated phagocytosis differs substantially from Fc-mediated phagocytosis, including in its kinetics. If CR3/CR4 engagement is occurring, it could provide an alternative explanation for many of the observed effects.

The authors should at minimum test phagocytosis of Fibcon-coated particles without IgG under integrin-blocking conditions, and/or use an integrin-blocking antibody in the main phagocytosis assay. This concern also connects to the question of Fibcon orientation raised below.

Response:

The Reviewer raises an important point that was also raised by Reviewer #2. As we explained in response to Comment #1 from Reviewer #2, we do not use native fibronectin in this study. Instead, we used as a building block an engineered, a hyperstable consensus FN-III domain ("Fibcon") [2] that contains no RGD domain. We expressed the Fibcons in *E. coli* to avoid glycosylation, which is known to increase fibronectin binding affinity [4]. In a previous study [3], we found that Fibcon repeats did not promote phagocytosis of target particles (see [3], Figure 2D, white bar plots), suggesting that our Fibcon repeat constructs do not significantly interact with CR3/CR4 receptors.

Moreover, our experimental data show that they do not increase particle adhesion to macrophages (Figure 3D, compare PSEL 0% bar to No IgG bar), suggesting that Fib5L does not interact with macrophage surface proteins.

Nevertheless, to further confirm that Fibcon repeats do not interact with the RAW cell surface, we incubated cells with fluorescent Fib1L, Fib3L, or Fib5L and quantified their recruitment at the cell surface. None showed detectable enrichment on the macrophage surface, unlike the WGA lectin positive control (see Response Figure 1). This strongly argues against an interaction between our Fibcon constructs and RAWs integrins and subsequent integrin-mediated phagocytosis.

Changes made in the manuscript:

- We included an additional control experiment testing our Fibcon constructs binding to macrophages surface (New Figure S2).
- We added a paragraph noting that the Fibcon constructs used in this study were previously tested in an identical experimental context and were found not to induce phagocytosis. (Line 150-155).

Major Comment 2

Fibcon protein conformation and orientation

The interpretation of bystander-mediated inhibition depends critically on the assumption that Fibcon proteins are standing upright on the bilayer surface and presenting their full extended height of 3.5, 10.5, or 17.5 nm. How is this confirmed? His-tag coupling to Ni-NTA lipids via a C-terminal tag constrains one end of the protein to the membrane, but does not prevent the protein from lying flat or adopting intermediate orientations, particularly at high surface densities. Random orientation would substantially reduce the effective steric barrier presented by the proteins, and would affect the quantitative interpretation of the density-dependent inhibition data. The authors should provide evidence for upright orientation, or at minimum discuss this as a limitation and consider its implications for the modeling, where rigid upright rods are assumed.

Response:

To characterize protein size, we used the *fully-extended contour length* as a common metric, since it can be easily estimated from the domain architecture. But the Reviewer is right to point out that these membrane-anchored proteins are not rigid rods. As semiflexible polymers, they undergo thermal fluctuations while retaining an average upright orientation [8].

The main consequence of this flexibility is that the mean extension of a multi-domain protein normal to the membrane is expected to be smaller than the fully extended contour length, as we showed in previous work with Fibcons ([9], Figure 2A). This work shows that multi-domain Fibcons behave like worm-like chain polymers with a persistence length of ~13 nm. Their mean effective height increases monotonically with contour length, consistent with our observation that increased numbers of domains produce a stronger steric barrier (Figure 1D, left). We have added a discussion of this point in the revised manuscript to acknowledge that the mean effective height of the Fibcons is below the full-extended contour length.

As for our Monte Carlo model that assumes upright stiff molecules, we have also altered the text to acknowledge that effective protein heights are expected to be shorter than their fully-extended contour lengths. This will shift quantitative thresholds without changing the qualitative expectation that an increased effective steric barrier impedes close-contact formation for short binders.

Changes made in the manuscript:

- We clarified why we chose the fully extended contour length as a metric for protein height in the paper and discussed implications in the texte. Line 159
- We discussed the limitation of the stiff model that we used in our monte Carlo simulation. (Line 495-505)

Major Comment 3

Confirmation of antibody presence at the microscopy interface

It should be explicitly confirmed that the anti-biotin IgG antibodies are present and functional at the interface in the TIRF experiments. While the fluorescence quantification implies this, a direct demonstration — for instance showing that antibody fluorescence is detected on the SLB prior to macrophage addition — would strengthen confidence in the experimental system, particularly for readers less familiar with supported lipid bilayer preparations.

Response:

We agree that confirmation of antibody on the SLB before macrophages are added would be useful. In Response Figure 3, we show representative TIRF images confirming that fluorescent anti-biotin IgG is present on the SLB before and after macrophage addition. The image on the right, after macrophage addition, shows accumulation of anti-biotin IgG below the macrophages, relative to the background IgG on the membrane. Contrast in the images has been scaled to make the IgG on the membrane visible, leading to saturation of the antibody signal at the macrophage interface.

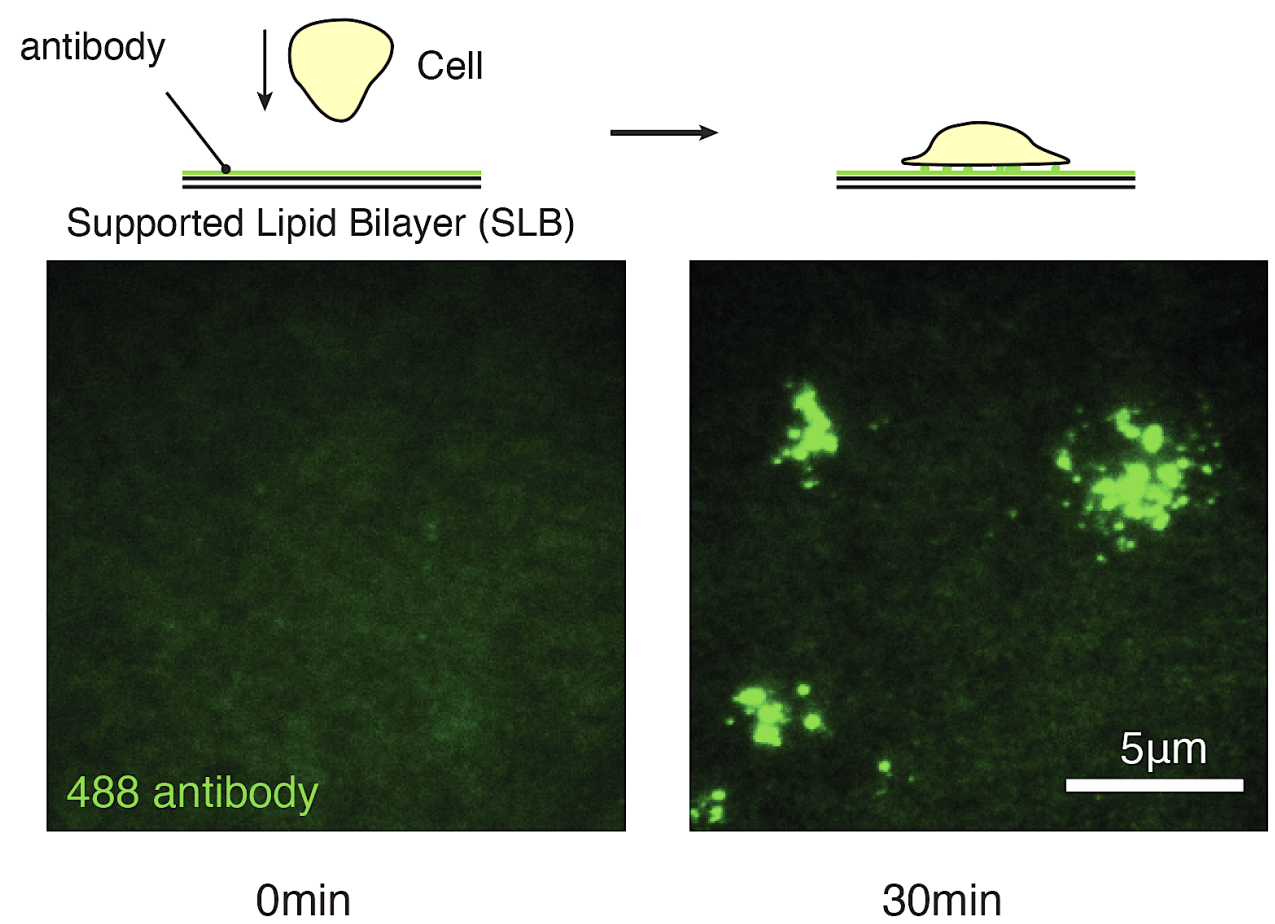

Response Figure 3: Representative TIRF images of the SLB decorated with Alexa Fluor 488 anti-biotin IgG1 before and after RAW macrophage spreading. Note that the 30 min image is saturated where macrophage enrich the antibody. Scale bar: 5 µm.

Major Comment 4

Kinetic versus thermodynamic framing is inconsistent.

The GUV experiments are presented as evidence that equilibrium thermodynamics is sufficient to explain the observed effects, yet throughout the paper the central phenomenon is described as "kinetic enhancement." This tension is never explicitly resolved. If the GUV system equilibrates and still shows bystander exclusion and short binder engagement in the presence of tall binders, this is informative about thermodynamic feasibility, but it does not by itself validate the kinetic mechanism proposed in Figure 5. The authors should clarify what the GUV experiments specifically demonstrate and how they relate to the kinetic framework, or revise the framing accordingly.

Response:

We thank the Reviewer and acknowledge that our wording blurred the kinetic and thermodynamic frameworks without clearly articulating their relationship.

In this manuscript, by “kinetic enhancement” we mean an increase in the rate (or probability over finite contact times) of reaching a close-contact state at the cell interface with the long binders compared to without. This close contact state is one that enables FcγR–IgG engagement in a crowded interface. Experimentally and theoretically, we find that close contact is not achieved when bystander proteins present a sufficiently large barrier, driving the rate of interface formation to nearly zero. In the presence of long binders, we find that the rate of close contact is significantly increased, despite the presence of the same bystander protein barrier, and we call that “kinetic

enhancement" of close contact formation. The proposed mechanism is inherently kinetic (dependent on finite timescales and barrier crossing) and dependent on the equilibrium and quasi-equilibrium states created by the binders.

With respect to the GUV experiments, our intent was to show that bystander exclusion and engagement of short receptors with the help of long binders is not dependent on active cellular forces (i.e., from actin-based protrusions). In the GUV context, short-binder engagement after 10 min (i.e., enrichment at the interface) was essentially absent in the presence of bystander proteins but was restored to short-binder-only levels when tall binders were introduced—an effect we also consider to be kinetic enhancement. In the revised manuscript, we clarify that the the GUV experiments establish a proof of principle for kinetic enhancement in a passive system, though with DNA binders instead of protein binders.

Changes made in the manuscript:

- Clarified definition of "kinetic enhancement" (Line 109-111).
- Revised the framing to remove ambiguity between "passive system" and "thermodynamic equilibrium" and to clarify how the GUV results relate to the kinetic framework. (Line 363-367)

Major Comment 5

P-selectin signaling not controlled for

The authors state that P-selectin was chosen in part because it "binds to the macrophage without directly activating or inhibiting phagocytic signaling." This is asserted as a design justification but is not demonstrated. P-selectin binding to PSGL-1 activates downstream signaling cascades in leukocytes, and it is not obvious that RAW 264.7 cells are an exception. The authors should either cite direct evidence that P-selectin/PSGL-1 engagement does not modulate phagocytic signaling in this cell line, perform an appropriate control experiment, or acknowledge this as a meaningful limitation of the interpretation. This is especially important given the close connection to integrin signaling.

Response:

We thank the Reviewer for raising this point that was also noted by Reviewer #2. As discussed in our response to Comment 2 of Reviewer #2, we cannot fully exclude downstream signaling from P-selectin/PSGL-1 engagement. However, our control experiments show that while P-selectin alone (no IgG) increases macrophage–particle association (Figure 3D), it does not increase phagocytosis (Figure 3E). This indicates that it acts primarily as an adhesion module and is not, by itself, a sufficient pro-phagocytic stimulus, at least under our experimental conditions. Accordingly, the rescue is most consistent with a biophysical mechanism in which added adhesion increases close membrane apposition and restores productive FcγR–IgG engagement when IgG is present.

Changes made in the manuscript:

- Added a section to the manuscript highlighting the Figure 3D–E controls showing that P-selectin alone is not a pro-phagocytic stimulus. (Line 310-314)

Major Comment 6

Latrunculin A experiment is underpowered and over-interpreted.

The LatA result is not surprising in isolation, since actin polymerization is a well-established requirement for phagocytosis. More importantly, the effect size is very modest: the enrichment index drops from approximately 1.25 to 1.1 with LatA treatment, compared to the much larger drop from 7.1 to 1.25 caused by bystander addition alone. This experiment is presented only as an inset at a single bystander density, yet the authors draw relatively strong conclusions about the role of active cytoskeletal processes in overcoming the steric barrier. This is also in tension with the thermodynamic interpretation of the GUV data. Either a more systematic treatment across bystander densities is needed, or the conclusions drawn from this experiment should be substantially tempered.

Response:

We thank the Reviewer for raising this point, which was also noted by Reviewer #1. As we wrote in response to Comment 2 of Reviewer #1, we re-checked our statistical analysis and realized that we had made an error in processing the LatA dataset. There is, in fact, no statistically significant difference between Fib5L 8000 and Fib5L 8000 + LatA. This result should have been expected since Fib5L already imposes an almost complete barrier, with almost no enrichment at 8000 molecules/μm^2. Under these conditions, inhibiting actin, which might otherwise help overcome the barrier by promoting protrusion formation, is expected to have no additional measurable effect. We have corrected the LatA statistical analysis and moved it to Supplementary Information, updating the text accordingly.

Changes made in the manuscript:

- Corrected the statistical analysis and moved the LatA data to the Supplementary Information (Now Figure S4).
- Updated the paragraph describing the LatA results to align with what the results support (Line 219-222).
- Expanded the discussion of actin and its possible role in the kinetic enhancement process (Discussion, Line 480-486)

Major Comment 7

All experiments use RAW 264.7 cells.

Given the therapeutic implications discussed at length, particularly regarding cancer immunotherapy and the proposed "Big-BiTE" concept, validation of at least the key findings in primary macrophages would be expected. The degree to which RAW 264.7 cells faithfully recapitulate the relevant biology of tumor-associated macrophages is not discussed

We agree with the Reviewer. To address the concern regarding the applicability of these findings to primary cells, we reproduced the key finding of the paper using bone marrow-derived macrophages. We showed that phagocytosis of Fib5L-decorated and opsonized targets is restored when PSEL is supplemented at the surface. The data are now presented in new supplementary Figure S5 and referenced in the text.

Changes made in the manuscript:

- Added new data demonstrating the kinetic enhancement concept in bone-derived macrophages (Line 315-321, Figure S5).

Major Comment 8

The “Big-BiTE” therapeutic proposal is speculative beyond what the data support.

The discussion section proposing a "Big-BiTE" therapeutic goes considerably beyond what the current data can support. This should be clearly and explicitly flagged as speculative, and the discussion of its potential clinical utility should be tempered accordingly.

Response:

We agree and have revised the Discussion to clearly label the “Big-BiTE” concept as speculative and to temper statements about clinical applicability. Our goal was simply to suggest a potential practical use for the fundamental concept we introduce in the manuscript, but we certainly want to be careful not to overstate it. We have updated the paragraph to emphasize a design principle (engineering interface transition states via tall adhesions) and state that translation would require extensive additional work.

Changes made in the manuscript:

- Rewrote the Discussion Big-BiTE paragraph to be explicitly speculative and more conservative (Line 523-526).

Moderate Comments

Comment 9

Statistics and data presentation are insufficient across multiple figures. This is a pervasive issue that affects Figures 1, 2, 3, 4, and S2:

- **Individual data points are not shown on any bar plots. Given the reported sample sizes, this should be straightforward to include, or violin plots should be substituted to convey the underlying distributions.**
- **Figures 1, 3, and S2 do not state the number of independent experiments or clarify whether error bars represent SD or SEM.**
- **Figure 2E uses pairwise t-tests for what is effectively a multiple comparison problem. ANOVA followed by an appropriate multiple comparison correction is required.**
- **Figure 3 reports no statistical tests despite claiming significant differences in the text.**
- **Figure S2 does not disclose which statistical test was used.**

These issues must be corrected consistently across all figures.

Response:

We agree and thank the Reviewer for pointing out these issues. In the revised manuscript and Supplementary Information, we have updated data presentation and statistical reporting to be consistent and transparent across all figures. Specifically, we have (i) shown individual data points corresponding to replicates, (ii) explicitly reported the number of independent experiments and the number of objects/cells analyzed, (iii) stated clearly whether error bars indicate SD, SEM, or confidence intervals, and (iv) ensured that the statistical tests reported match the displayed summary statistics.

Changes made in the manuscript:

- Updated plots to display distributions (individual points, SD) consistently.
- Added explicit reporting of N and clarified SD vs SEM.
- Updated statistical testing to include nul hypothesis comparison and added missing tests where needed.

Comment 10

Methods descriptions are insufficient. Several aspects of the methods need clarification:

- **How was the phagocytic index determined from the fluorescence images? The automated CellProfiler pipeline is referenced but not described.**
- **How were fields of view selected for imaging? Some description of how human bias in FOV selection was minimized is needed.**

- **Which microscope, objective, and acquisition settings were used for the phagocytosis imaging?**

Response:

We agree and have updated our Methods descriptions.

Changes made in the manuscript:

In the revised Methods we expanded the description of the image analysis and acquisition workflow:

- We provided a clearer description of how the phagocytic index was computed from fluorescence images, including the key segmentation/thresholding steps and how bead association/internalization was quantified (see *Assaying phagocytosis*).
- We described how fields of view were selected and what steps were taken to minimize selection bias (Line 870)
- We specified the microscope model, objective, illumination mode, camera, acquisition settings, and any relevant preprocessing steps.

- We added a hardware section listing the materials used (Line 974).

Comment 11

Figure 1D: "Fib5L MPE" is undefined at first appearance. The label "Fib5L MPE" appears in Figure 1D without definition in either the figure caption or the surrounding text at that point. The term (membrane-proximal epitope) is only explained later, which creates confusion on first reading. It should be defined at first use.

Response:

We agree and apologize for the lack of clarity. We will define "MPE" (membrane-proximal epitope) at its first appearance in the main text and/or in the Figure 1 caption, and we will ensure consistent usage throughout the manuscript.

Changes made in the manuscript:

- Defined "MPE" at first use in Figure 1D caption (Line 677-678), and corresponding Results section (Line 183-185).

Comment 12

Figure 2C enrichment index heterogeneity. The no-bystander condition in Figure 2C shows an extremely wide distribution of enrichment index values, with the box extending to approximately 35. This heterogeneity is never discussed. Are some macrophages non-responsive? Does cell-to-cell variability in FcγR expression or actin dynamics account for this spread? Some discussion of this point would help the reader interpret the data.

Response:

We thank the Reviewer for raising this point. We rechecked our data and found that the large spread in the 0% bystander condition was driven by a single experimental replicate with unusually high enrichment index values (22 ± 19). After re-examining the images, we noticed a substantial focus shift at the 30 min time point used to assess the enrichment index.

To address this issue, we re-ran the analysis using the frame acquired 30 seconds earlier, when the focus was still consistent. With this corrected time point, the enrichment index for the 0% bystander condition was 6 ± 4.

This Reviewer comment prompted us to improve how we present the data distribution and the replicate structure. We now plot the mean of triplicate enrichment index measurements as a function of Fib5L density on the SLB, and the updated analysis confirms that the enrichment index decreases significantly with increasing Fib5L density (Figure 2C, test against nul hypothesis). We made the same updates for Figure 2E, and we confirm that the time to enrichment increases significantly with increasing Fib5L density (test against nul hypothesis).

Changes made in the manuscript:

- Updated the data presentation in Figures 2C and 2E and captions
- Refined the statistical analysis.
- Updated the text to account for these changes.

Comment 13

Conclusion from Figure S2 / lines 181–186 is not well supported (N unclear, no SD, test not disclosed). The conclusion that Fib3L antigen performs better than others in the presence of Fib5L bystanders is stated with more confidence than the data in Figure S2 support. The N is unclear, no SD is shown, and the statistical test used is not disclosed. This should be corrected in line with the general statistics comments above.

Response:

We thank the Reviewer for noting this inadvertent omission. In the revision, we have corrected the presentation of Figure S2 (now Figure S3) to match the standards applied to the main figures. We have clearly stated the number of independent experiments and sample sizes, display variability (individual points and/or appropriate error bars), and disclose the statistical test used.

Changes made in the manuscript:

- Updated Figure S2 (now Figure S3) to show N and variability and added the statistical test description.
- Revised the corresponding conclusion to match the supported confidence level.

<u>Minor Comments</u>

Comment 14

Protein density comparison for P-selectin. The comparison between experimentally used P-selectin densities and those on activated platelets is presented with more confidence than the underlying data warrant. The platelet density is back-calculated from two indirect measurements and the uncertainty in this estimate should be acknowledged.

Response:

We agree. In the revised manuscript, we have qualified the comparison between our experimental P-selectin densities and estimated platelet surface densities. We have revised the text to ensure that it presents this comparison as an order-of-magnitude contextualization rather than a precise quantitative match.

Changes made in the manuscript:

- Revised text to avoid overconfident density comparisons (Line 295-298).

Comment 15

Equation display errors in the SI (extraneous boxes). There are formatting errors in how several equations are displayed, with extraneous boxes appearing in the text. These should be corrected in revision.

Response:

We agree and have corrected the SI formatting so that equations render properly (including after journal conversion/production). We will re-export and verify the SI PDF to ensure no missing symbols or spurious boxes remain.

Changes made in the manuscript:

- Corrected SI equation formatting and verified display in the revised SI PDF.

Comment 16

Modeling assumes rigid upright proteins. Related to comment 2 above, the Monte Carlo model treats all proteins as rigid upright rods. The authors briefly mention in the discussion that protein flexibility could affect the energetics of the intermediate state, but given that this assumption also underlies the experimental interpretation of Fibcon orientation, a more substantive discussion of how deformable or obliquely oriented proteins would change the predictions would be valuable.

Response:

We agree and have expanded the discussion of this modeling assumption, which was also noted by Reviewer #1 in Comment 4. In the revised Discussion, we explain how conformational heterogeneity could change effective steric height and alter quantitative thresholds for barrier formation and rescue. We clarify that rigid upright rods represent a simplifying limit, and we discuss qualitatively how introducing flexibility could (i) lower effective barriers in some regimes, (ii) broaden transition-state separations, or (iii) allow certain long flexible binders to remain at interfaces in ways the current model cannot capture.

Changes made in the manuscript:

- Expanded the discussion of the limitations and interpretation of the rigid-rod assumption (Line 495-505).
- Clarified in the Materials and methods section that the molecules in the Monte Carlo simulations are rod-like and remain oriented parallel to the lattice normal. Their orientation therefore fluctuates as the lattice deforms (Line 957-960).

References

[1] M. Diakonova, G. Bokoch, and J. A. Swanson, "Dynamics of cytoskeletal proteins during Fcgamma receptor-mediated phagocytosis in macrophages," *Mol. Biol. Cell*, vol. 13, no. 2, pp. 402–411, Feb. 2002.

[2] S. A. Jacobs, "Design of novel FN3 domains with high stability by a consensus sequence approach," *Protein Eng. Des. Sel.*, vol. 25, pp. 107–117, 2012.

[3] M. H. Bakalar, “Size-dependent segregation controls macrophage phagocytosis of antibody-opsonized targets,” *Cell*, vol. 174, pp. 131-142.e13, 2018.
[4] C.-T. Hsiao *et al.*, “Fibronectin in cell adhesion and migration via N-glycosylation,” *Oncotarget*, vol. 8, no. 41, pp. 70653–70668, Sept. 2017.
[5] R. Tinoco, D. C. Otero, A. A. Takahashi, and L. M. Bradley, “PSGL-1: A new player in the immune checkpoint landscape,” *Trends Immunol.*, vol. 38, no. 5, pp. 323–335, May 2017.
[6] P. Bruhns, “Properties of mouse and human IgG receptors and their contribution to disease models,” *Blood*, vol. 119, no. 24, pp. 5640–5649, June 2012.
[7] G. Dekkers *et al.*, “Affinity of human IgG subclasses to mouse Fc gamma receptors,” *MAbs*, vol. 9, no. 5, pp. 767–773, July 2017.
[8] A. Chorlay, S. Agarwal, L. Blackmon, A. Dextre, and D. A. Fletcher, “SurFlex microscopy: Measuring flexibility of surface-tethered biomolecules,” *Proc. Natl. Acad. Sci. U. S. A.*, vol. 122, no. 48, p. e2508828122, Dec. 2025.
[9] S. Son, S. C. Takatori, B. Belardi, M. Podolski, M. H. Bakalar, and D. A. Fletcher, “Molecular height measurement by cell surface optical profilometry (CSOP),” *Proc. Natl. Acad. Sci. U. S. A.*, vol. 117, no. 25, pp. 14209–14219, June 2020.

Point-by-point "Response to Reviewers"

(Manuscript accepted in principle)

Response to Reviewer #1

Reviewer #1 (Remarks to the Author):

Authors addressed my concerns. I recommend the publication of the manuscript.

Response:

We thank the reviewer for their feedback, which helped us improve the manuscript.

Response to Reviewer #2

The reviewers have addressed most of my concerns. I would like to point out that the statement that only FcgRIII but not FcgRIIb is a phagocytic receptor needs to be corrected. Especially FcgRIIb on macrophages is known to be a very efficient receptor triggering receptor mediated endocytosis. Apart from that I have no further concerns.

Response:

We thank the reviewer for pointing out this inaccuracy. We have corrected it on lines 183-185 of the main text. We are pleased that the reviewer considers their concerns to have been satisfactorily addressed.

Response to Reviewer #3

The revised manuscript adequately addresses my prior concerns. This is an interesting and exciting study that uses exceptional experimental design to advance our understanding of physical interactions beyond antibody-FcR interactions that are involved in antibody-dependent phagocytosis.

Response:

We thank the reviewer for their positive assessment of our revised manuscript.

Response to Reviewer #4

I commend the authors for a nice revision of this paper. I believe all of my major concerns have been addressed or clarified nicely.

Response:

We thank the reviewer for their encouraging assessment of our revised manuscript and are pleased that the reviewer considers the major concerns to have been satisfactorily addressed.
