# Supplementary material for: Tall adhesions overcome cell surface steric interference and rescue antibody-dependent phagocytosis in macrophages
Source: Nat Commun. 2026 Sep 28;17:11213. doi: 10.1038/s41467-026-78095-7 (PMC13720194; doi:10.1038/s41467-026-78095-7)
Supplement: Supplementary file 3 [file 41467_2026_78095_MOESM3_ESM.pdf]

nature portfolio

Corresponding author(s): Daniel A. Fletcher

Last updated by author(s): Jul 23, 2026

Reporting Summary

Nature Portfolio wishes to improve the reproducibility of the work that we publish. This form provides structure for consistency and transparency in reporting. For further information on Nature Portfolio policies, see our Editorial Policies and the Editorial Policy Checklist.

Statistics

For all statistical analyses, confirm that the following items are present in the figure legend, table legend, main text, or Methods section.

n/a	Confirmed	
☐	☒	The exact sample size (*n*) for each experimental group/condition, given as a discrete number and unit of measurement
☐	☒	A statement on whether measurements were taken from distinct samples or whether the same sample was measured repeatedly
☐	☒	The statistical test(s) used AND whether they are one- or two-sided<br>*Only common tests should be described solely by name; describe more complex techniques in the Methods section.*
☒	☐	A description of all covariates tested
☒	☐	A description of any assumptions or corrections, such as tests of normality and adjustment for multiple comparisons
☐	☒	A full description of the statistical parameters including central tendency (e.g. means) or other basic estimates (e.g. regression coefficient) AND variation (e.g. standard deviation) or associated estimates of uncertainty (e.g. confidence intervals)
☐	☒	For null hypothesis testing, the test statistic (e.g. *F*, *t*, *r*) with confidence intervals, effect sizes, degrees of freedom and *P* value noted<br>*Give P values as exact values whenever suitable.*
☒	☐	For Bayesian analysis, information on the choice of priors and Markov chain Monte Carlo settings
☒	☐	For hierarchical and complex designs, identification of the appropriate level for tests and full reporting of outcomes
☒	☐	Estimates of effect sizes (e.g. Cohen's *d*, Pearson's *r*), indicating how they were calculated

Our web collection on statistics for biologists contains articles on many of the points above.

Software and code

Policy information about availability of computer code

Data collection	To collect flow cytometry data for this manuscript, Attune Cytometric Software was used. To collect image data for this manuscript, Nikon Elements was used.
Data analysis	Microscopy image analysis was performed with Fiji (v2.16.0/154p). Flow cytometry FCS files were analyzed in FlowJo v10.8.1. Statistical analyses and graph plotting were performed with Prism v11.0.1 (99). Quantification from microscopy images was performed with CellProfiler (Broad Institute v4.2.8)

For manuscripts utilizing custom algorithms or software that are central to the research but not yet described in published literature, software must be made available to editors and reviewers. We strongly encourage code deposition in a community repository (e.g. GitHub). See the Nature Portfolio guidelines for submitting code & software for further information.

Data

Policy information about availability of data

All manuscripts must include a data availability statement. This statement should provide the following information, where applicable:

- Accession codes, unique identifiers, or web links for publicly available datasets
- A description of any restrictions on data availability
- For clinical datasets or third party data, please ensure that the statement adheres to our policy

All the source data that has been collected is available in the Source data file submitted to nature communication.

Research involving human participants, their data, or biological material

Policy information about studies with human participants or human data. See also policy information about sex, gender (identity/presentation), and sexual orientation and race, ethnicity and racism.

Reporting on sex and gender	*Use the terms sex (biological attribute) and gender (shaped by social and cultural circumstances) carefully in order to avoid confusing both terms. Indicate if findings apply to only one sex or gender; describe whether sex and gender were considered in study design; whether sex and/or gender was determined based on self-reporting or assigned and methods used.*<br>*Provide in the source data disaggregated sex and gender data, where this information has been collected, and if consent has been obtained for sharing of individual-level data; provide overall numbers in this Reporting Summary. Please state if this information has not been collected.*<br>*Report sex- and gender-based analyses where performed, justify reasons for lack of sex- and gender-based analysis.*
Reporting on race, ethnicity, or other socially relevant groupings	*Please specify the socially constructed or socially relevant categorization variable(s) used in your manuscript and explain why they were used. Please note that such variables should not be used as proxies for other socially constructed/relevant variables (for example, race or ethnicity should not be used as a proxy for socioeconomic status).*<br>*Provide clear definitions of the relevant terms used, how they were provided (by the participants/respondents, the researchers, or third parties), and the method(s) used to classify people into the different categories (e.g. self-report, census or administrative data, social media data, etc.)*<br>*Please provide details about how you controlled for confounding variables in your analyses.*
Population characteristics	*Describe the covariate-relevant population characteristics of the human research participants (e.g. age, genotypic information, past and current diagnosis and treatment categories). If you filled out the behavioural & social sciences study design questions and have nothing to add here, write "See above."*
Recruitment	*Describe how participants were recruited. Outline any potential self-selection bias or other biases that may be present and how these are likely to impact results.*
Ethics oversight	*Identify the organization(s) that approved the study protocol.*

Note that full information on the approval of the study protocol must also be provided in the manuscript.

Field-specific reporting

Please select the one below that is the best fit for your research. If you are not sure, read the appropriate sections before making your selection.

☒ Life sciences ☐ Behavioural & social sciences ☐ Ecological, evolutionary & environmental sciences

For a reference copy of the document with all sections, see nature.com/documents/nr-reporting-summary-flat.pdf

Life sciences study design

All studies must disclose on these points even when the disclosure is negative.

Sample size	No sample-size calculation was performed. For each experiment, three biological replicates were performed on three different days.
Data exclusions	No data was excluded from the manuscript.
Replication	All experiments were repeated at least three times on three different days to ensure reproducibility. Variation is reflected in the error bars present on figures in the manuscript and described in figure captions. All attempts at replication were successful.
Randomization	This is not relevant to our study as we were investigating cell behavior in cultured immortalized cell lines. Experiments done with bone marrow derived macrophages were performed on cells derived from one mouse.
Blinding	Investigators were not blinded during data acquisition and analysis. As the analytical methods employed require little-to-no subjective input from the investigators, this did not seem necessary.

Reporting for specific materials, systems and methods

We require information from authors about some types of materials, experimental systems and methods used in many studies. Here, indicate whether each material, system or method listed is relevant to your study. If you are not sure if a list item applies to your research, read the appropriate section before selecting a response.

Materials & experimental systems

n/a	Involved in the study
☐	☒ Antibodies
☐	☒ Eukaryotic cell lines
☒	☐ Palaeontology and archaeology
☒	☐ Animals and other organisms
☒	☐ Clinical data
☒	☐ Dual use research of concern
☒	☐ Plants

Methods

n/a	Involved in the study
☒	☐ ChIP-seq
☐	☒ Flow cytometry
☒	☐ MRI-based neuroimaging

Antibodies

Antibodies used

Antibodies used to opsonize glass beads were purchased from Santa Cruz Biotechnology and used without further labelling or purification. Biotin was bound by Anti-biotin IgG (eBioscience, clone BK-1/39, Alexa Fluor 488 label) with a 1:4000 dilution in PBS buffer.

Validation

Anti-biotin ntibodies were validated for use in Western Blots or Immunofluorescence. The manufacturer's website provides a list of citations for each antibody as well as details about the validation. We used the antibodies in this study without further purification or labeling.

Eukaryotic cell lines

Policy information about cell lines and Sex and Gender in Research

Cell line source(s)

1. RAW 264.7 cells: Mouse origin, male, acquired from the Barker Cell Culture Facility at UC Berkeley.
2. BMDM cells: from male Wildtype (C57BL/6, Stock. No. 000664) mice were a gift from the Robey Lab (UC Berkeley. The mice was originally purchased from the Jackson Laboratory.

Authentication

All cell lines from the Barker Cell Culture Facility at UC Berkeley are authenticated by the facility.

Mycoplasma contamination

All cell lines tested negative for mycoplasma contamination.

Commonly misidentified lines (See ICLAC register)

No cell lines commonly misidentified per ICLAC register were used

Plants

Seed stocks

Report on the source of all seed stocks or other plant material used. If applicable, state the seed stock centre and catalogue number. If plant specimens were collected from the field, describe the collection location, date and sampling procedures.

Novel plant genotypes

Describe the methods by which all novel plant genotypes were produced. This includes those generated by transgenic approaches, gene editing, chemical/radiation-based mutagenesis and hybridization. For transgenic lines, describe the transformation method, the number of independent lines analyzed and the generation upon which experiments were performed. For gene-edited lines, describe the editor used, the endogenous sequence targeted for editing, the targeting guide RNA sequence (if applicable) and how the editor was applied.

Authentication

Describe any authentication procedures for each seed stock used or novel genotype generated. Describe any experiments used to assess the effect of a mutation and, where applicable, how potential secondary effects (e.g. second site T-DNA insertions, mosiacism, off-target gene editing) were examined.

Flow Cytometry

Plots

Confirm that:

☒ The axis labels state the marker and fluorochrome used (e.g. CD4-FITC).

☒ The axis scales are clearly visible. Include numbers along axes only for bottom left plot of group (a 'group' is an analysis of identical markers).

☒ All plots are contour plots with outliers or pseudocolor plots.

☒ A numerical value for number of cells or percentage (with statistics) is provided.

Methodology

Sample preparation

RAW264.7 cells were labeled with CellTracker Green CMFDA and washed 2× with PBS, followed by final resuspension in cell medium. Cells were diluted to a concentration of 350 cells/µL, and 100 µL of cells were added to each tube. 100 µL of target particle suspension containing ~500,000 particles was added to each tube, and the tubes were incubated for 30 minutes at 4

	°C to prevent phagocytosis and under constant rotation to prevent settling. After 30 minutes, each tube was run through a flow cytometer (Attune NxT)
Instrument	The instrument used was an Attune NxT Flow Cytometer.
Software	Flow cytometry data was collected using the Attune Cytometric Software. Exported FCS files were then analyzed in FlowJo_v10.8.1.
Cell population abundance	Cells were only analyzed on the flow cytometer, they were not sorted and further experimented upon.
Gating strategy	To determine macrophaghes that were bound to target particles: macrophages were gated by high CellTracker Green CMFDA signal. Macrophages bound to target particles were gated based on the DOPE-Atto647 high signal. Figure 3C describes the gating strategy.

☒ Tick this box to confirm that a figure exemplifying the gating strategy is provided in the Supplementary Information.
